# A New PNA-FISH Probe Targeting *Fannyhessea vaginae*

**DOI:** 10.3389/fcimb.2021.779376

**Published:** 2021-11-18

**Authors:** Lúcia G. V. Sousa, Joana Castro, Angela França, Carina Almeida, Christina A. Muzny, Nuno Cerca

**Affiliations:** ^1^ Laboratory of Research in Biofilms Rosário Oliveira (LIBRO), Centre of Biological Engineering (CEB), Campus de Gualtar, University of Minho, Braga, Portugal; ^2^ INIAV, IP- National Institute for Agrarian and Veterinary Research, Vila do Conde, Portugal; ^3^ Division of Infectious Diseases, University of Alabama at Birmingham, Birmingham, AL, United States

**Keywords:** *Fannyhessea vaginae*, *Gardnerella vaginalis*, bacterial vaginosis, fluorescence *in situ* hybridization (FISH), peptide nucleic acid (PNA)

## Abstract

Bacterial vaginosis (BV) is the most common vaginal infection in women of reproductive age and has been associated with serious health complications, mainly in pregnant women. It is characterized by a decrease in the number of *Lactobacillus* species in the healthy vaginal microbiota and an overgrowth of strict and facultative anaerobic bacteria that develop a polymicrobial biofilm. Despite over 60 years of research investigating BV, its etiology is not fully understood. *Gardnerella* spp. is a crucial microorganism that contributes to the formation of the biofilm and the development of BV, but the role of other BV-associated bacteria is not clear. Nevertheless, *Fannyhessea vaginae* (previously known as *Atopobium vaginae*) is a highly specific species for BV, and co-colonization with *Gardnerella* is thought to be a very specific diagnostic marker. The diagnosis of BV still presents some limitations, since currently used methods often fail to accurately detect BV. This work aims to develop a novel peptide nucleic acid (PNA) probe targeting *F. vaginae*. This probe was further validated in a multiplex assay, which included a *Gardnerella-*specific PNA probe, as a possible method for diagnosis of BV, and was compared with quantification by qPCR. The new PNA probe showed excellent sensitivity and specificity and could discriminate *F. vaginae*-*Gardnerella* biofilms, confirming the potential to be used for the detection of BV-associated pathogens.

## Introduction

Bacterial vaginosis (BV) is the most common vaginal infection in women of reproductive age (Jung et al., 2017) affecting around 23% to 29% of women worldwide and it is associated with high healthcare costs (Peebles et al., 2019). BV is characterized by vaginal discharge and odor, an increase in the vaginal pH, as well as the presence of clue cells (Sobel, 2000; Livengood, 2009; Hay, 2014). BV has been associated with multiple health complications, including adverse birth outcomes (Svare et al., 2006; Isik et al., 2016). Microbiologically, BV is characterized by a decrease in commensal, protective lactobacilli and a dramatic increase in strict and facultative anaerobic bacteria which form a polymicrobial biofilm on the surface of the vaginal epithelial cells (Turovskiy et al., 2011; Rosca et al., 2020b).

The diagnosis of BV is usually performed using clinical criteria or microbiologically by interpretation of vaginal Gram-stains (Gutman et al., 2005). The most common method for BV diagnosis is Amsel’s criteria, which is based on criteria related to the clinical signs of BV. These criteria include (i) homogeneous vaginal discharge, (ii) vaginal pH greater than 4.5, (iii) the release of a fishy smell on the addition of 10% potassium hydroxide to a drop of vaginal discharge, and (iv) the presence of clue cells (Amsel et al., 1983). BV is considered present when at least three of the four Amsel’s criteria are detected (Turovskiy et al., 2011; Hay, 2014). An alternative method for the diagnosis of BV is the analysis of Gram-stains of vaginal fluid, proposed by Nugent *et al.*, (Nugent et al., 1991). According to the Nugent method, Gram-stain smears are classified by the presence of different bacterial morphotypes and scored on a 0 to 10 scale by the sum of the quantification (0 to 4+) of each morphotype (Nugent et al., 1991). A smear with a Nugent score of 0 to 3 is considered normal, a score of 4 to 6 is considered intermediate, and a score equal to or greater than 7 is considered positive for BV (Nugent et al., 1991). Due to the limitations of conventional methods of diagnosis, when comparing these two methods to one another, the Amsel criteria shows values of sensitivity between 37% and 70% and specificity between 94% and 99% (Schwebke et al., 1996; Sha et al., 2005; Modak et al., 2011). When evaluating the Nugent method using the Amsel criteria as reference, the values of sensitivity and specificity range from 78% to 94% and 67% to 94%, respectively, (Schwebke et al., 1996) and therefore, more reliable and accurate alternatives for the detection of bacteria associated with BV are needed to improve diagnosis (Africa, 2013; Redelinghuys et al., 2020).

*Gardnerella* spp. have been recognized as the most common bacteria present in BV and play an important role in the pathogenesis of BV (Muzny et al., 2019; Rosca et al., 2020b). Despite its high prevalence in cases of BV, *Gardnerella* is also found in women who do not have BV (Hickey and Forney, 2014). Importantly, *Gardnerella* spp. may exhibit several virulence factors that explain its pathogenic potential in women with BV (Janulaitiene et al., 2018), such as the ability to displace *Lactobacillus* adhered to vaginal epithelial cells (Castro et al., 2015), the high capacity to form biofilm (Harwich et al., 2010), greater cytotoxic activity by the production of vaginolysin (Patterson et al., 2010), which lyses vaginal epithelial cells (Gelber et al., 2008), and the presence of sialidase that leads to exfoliation of vaginal epithelial cells (Santiago et al., 2011; Lewis et al., 2013). These characteristics suggest that *Gardnerella* spp. is a crucial microorganism for the development of BV, which seems to adhere to the vaginal epithelial cells and initiate the formation of a biofilm to which other BVAB consequently attach and interact with each other, inducing the infection (Muzny et al., 2019). *Fannyhessea vaginae*, previously known as *Atopobium vaginae* (Nouioui et al., 2018), is highly specific for BV, as it is also found in most BV cases but is rarely present in the vaginal microbiota of healthy women (Bradshaw et al., 2006b). For this reason, *F. vaginae* is considered an indicator for abnormal vaginal microbiota and it is more specific to BV than *Gardnerella* spp. (Sehgal et al., 2021). The combination of *F. vaginae* and some *Gardnerella* spp. could be the best diagnostic method of BV (Menard et al., 2008). However, as a fastidious microorganism, *F. vaginae* is difficult to detect by culture-dependent methods (Trama et al., 2008). As such, new molecular approaches to identify *F. vaginae* in polymicrobial BV biofilms are necessary.

Molecular methods targeting nucleic acids are important approaches for BV diagnosis since they can identify multiple microorganisms associated with the infection (Coleman and Gaydos, 2018; Redelinghuys et al., 2020). Fluorescence *in situ* hybridization (FISH) is a molecular technique that uses probes specifically designed to target a microorganism of interest (Prudent and Raoult, 2019). Some advances in the development of FISH lead to an increase in the use of peptide nucleic acid (PNA) probes, which are polymeric neutral charged probes that bind to DNA or RNA without repulsion (Cerqueira et al., 2008). Peptide nucleic acid fluorescence *in situ* hybridization (PNA-FISH), which includes a PNA probe specifically designed to target a microorganism of interest, has been used as an alternative for the diagnosis of infections and detection of particular bacterial species (Almeida et al., 2011; Prudent and Raoult, 2019). The application of PNA-FISH methodology for the detection of bacteria related to BV has been proposed, however still with a limited number of targeted species. Previously, only one PNA probe targeting *Gardnerella vaginalis* (Machado et al., 2013) and three PNA probes targeting *F. vaginae* (Hardy et al., 2015) were designed and developed specifically for the study of BV. While the *Gardnerella* probe has been shown to have high sensitivity and specificity (Machado et al., 2013), the currently available *F. vaginae* probes have lower efficiency (Hardy et al., 2015). In this work, we aimed to develop a novel PNA probe targeting *F. vaginae*, in an attempt to improve BV diagnostic accuracy and further BV pathogenesis research. We also assessed whether the probe could be used in a multiplex assay, to discriminate species within a biofilm.

## Materials and Methods

### *In Silico* Design of *F. vaginae* PNA Probe

To identify potential oligonucleotides for the *F. vaginae* probe, we selected a set of sequences from 16S and 23S collections, with lengths >1200 bp or >1600 bp, respectively, available at Arb-Silva database (https://www.arb-silva.de/search/). Only sequences with a quality score >90 were considered. Each set contained sequences from *F. vaginae* strains, as well as species from the same genus and other genera closely related to the bacterium of interest. The sequences were then aligned using the Clustal Omega tool (https://www.ebi.ac.uk/Tools/msa/clustalo/). Regions for potential probes were searched, showing the same sequence in the species of interest and one or more mismatches in the sequences belonging to other species. Theoretical sensitivity and specificity of the PNA probes were determined, as previously described (Almeida et al., 2013). The probes were evaluated using the TestProbe tool (https://www.arb-silva.de/search/testprobe/) with no mismatches allowed. Sequences with the highest theoretical sensitivity and specificity, complementarity with a low number of non-interest sequences, GC content between 40% and 60%, high melting temperature (>50°C) (Almeida et al., 2011), and Gibbs free energy ranging from -13 kcal/mol to -20 kcal/mol (Yilmaz and Noguera, 2004) were selected as the best probes. The selected probe was then synthesized (Eurogentec, Seraing, Belgium) and the oligonucleotide N-terminus was linked to an Alexa Fluor molecule *via* a double 8-amino-3,6-dioxaoctanoic acid linker (*F. vaginae* probe: Alexa Fluor 488-OO-CGATGTGCGACTAAA).

### Bacterial Growth Conditions

*F. vaginae* ATCC BAA-55, *F. vaginae* CCUG 42099, *F. vaginae* CCUG 44116, and 21 other *F. vaginae* strains, previously isolated from cases of BV (De Backer et al., 2006; De Backer et al., 2010; Santiago et al., 2012), were used to determine *F. vaginae* probe analytical sensitivity. Forty different bacterial species associated with BV or with the vaginal microbiota, were used to determine *F. vaginae* probe analytical specificity. The strains were grown in Columbia Blood Agar Base (Oxoid, Basingstoke, UK) supplemented with 5% (v/v) of defibrinated horse blood (Oxoid) for 24 or 48 h, except for *Sneathia sanguinegens* which was maintained in chocolate agar supplemented with 10% (v/v) inactivated horse serum (Biowest, Nuaillé, France) for 48 h. *Actinomyces urogenitalis, Aerococcus christensenii, Bifidobacterium bifidum, Campylobacter ureolyticus, F. vaginae* strains, *Lactobacillus iners*, *Megasphaera micronuciformis*, *Mobiluncus curtisii*, *M. mulieris*, *Mycoplasma hominis*, *Peptostreptococcus anaerobius, Porphyromonas asaccharolytica, Prevotella bivia, Propionibacterium acnes, S. sanguinegens* and *Veillonella parvula* were kept at 37°C under anaerobic conditions (AnaeroGen Atmosphere Generation system, Oxoid). *Acinetobacter baumannii* was grown at 30°C and the remaining species were maintained at 37°C and 10% CO_2_.

### FISH Hybridization Procedure

For PNA-FISH experiments, a bacterial suspension was prepared in phosphate-buffered saline (PBS), in which we first adjusted it to an optical density (OD) at 620 nm ~ 0.1 and then we performed a 2-fold dilution. Afterward, 30 µL of the suspension was spread on epoxy coated microscope glass slides (Thermo Fisher Scientific, Lenexa, KS) and air-dried. Optimization experiments were performed to identify the optimal hybridization conditions which resulted in the best fluorescence signal. Variations in the hybridization temperature, ranging from 50°C-63°C, and time of 60 min and 90 min were tested using the strain *F. vaginae* ATCC BAA-55. At the optimized conditions, the cells were fixed with 100% (v/v) methanol (Thermo Fisher Scientific) for 15 min, 4% (w/v) paraformaldehyde (Thermo Fisher Scientific) for 10 min, followed by 50% (v/v) ethanol (Thermo Fisher Scientific) for 15 min, and allowed to dry. After, 20 µL of hybridization solution containing 10% (w/v) dextran sulfate (Sigma, Germany), 10 mM NaCl (Sigma), 30% (v/v) formamide (Thermo Fisher Scientific), 0.1% (w/v) sodium pyrophosphate (Thermo Fisher Scientific), 0.2% (w/v) polyvinylpyrrolidone (Sigma), 0.2% (w/v) Ficoll (Sigma), 5 mM disodium EDTA (Panreac, Spain), 0.1% (v/v) Triton X-100 (Thermo Fisher Scientific), 50 mM Tris–HCl (pH 7.5; Thermo Fisher Scientific), and 200 nM of PNA probe were applied to the slides and covered with a coverslip. The slides were placed in a moist and opaque container and incubated at the selected testing time/temperature. After that, the coverslips were removed, and the slides were immersed in the pre-warmed washing solution containing 5 mM Tris-base (Thermo Fisher Scientific), 15 mM NaCl (Sigma), and 1% (v/v) Triton-X (pH 10; Thermo Fisher Scientific) and incubated for 30 min at the same temperature as hybridization. Hybridization was performed at 56°C for 60 min. For the multiplex experiments in biofilms, a PNA probe specific for *Gardnerella* (Machado et al., 2013) was added to the hybridization solution at the same concentration of 200 nM.

### Microscopic Analysis

Microscopic visualization was performed using an Olympus BX51 epifluorescence microscope (Olympus, Lisbon, Portugal) equipped with a FITC filter (BP 470-490, FT500, LP 516 sensitive to the Alexa Fluor 488 molecule). Filters that do not detect the probe fluorescence were used as controls to confirm if the cells did not have auto-fluorescence. For every experiment, a negative control was performed with hybridization solution without a probe. The experiments were performed with at least two independent assays.

### Biofilm Formation and Confocal Laser Scanning Microscopy (CLSM)

Inoculums of *G. vaginalis* ATCC 14018 and *F. vaginae* ATCC BAA-55 were prepared in New York City III (NYCIII) broth (Rosca et al., 2020a) supplemented with 10% (v/v) inactivated horse serum and incubated for 24 h at 37°C under anaerobic conditions. After 24 h, the bacterial concentration was adjusted to 1 × 10^7^ CFU/mL in NYCIII broth and biofilms were formed on eight-well chamber slides (Thermo Fisher Scientific™ Nunc™ Lab-Tek™, Rochester, NY, USA) by inoculating each of the respective species for single biofilms and both species for dual-species biofilms, for a final volume of 400 µL. Biofilms were incubated at 37°C in anaerobic conditions. After 24 h, the medium was removed, and the biofilm was washed once with NaCl and air-dried. Subsequently, fixation was performed (as described above) and PNA-FISH was conducted at 60°C for 90 min using the *F. vaginae* and *G. vaginalis* (Machado et al., 2013) probes. CLSM images were acquired using an Olympus™ Fluo-View FV1000 (Olympus) confocal laser scanning microscope. The experiments were performed in duplicate.

### Bacterial Species Discrimination in Dual-Species Biofilms by PNA-FISH

The bacterial population within dual-species biofilms of *G. vaginalis* and *F. vaginae* was differentiated by PNA-FISH, as described previously (Castro et al., 2020). Briefly, non-adherent cells were removed by one gentle wash with PBS and, afterward, biofilms were scraped vigorously from the well. Then, 30 μL of each resuspended biofilm was spread on epoxy-coated microscope glass slides (Thermo Fisher Scientific) and the FISH procedure was performed as described above. Microscopic visualization was performed using filters capable of detecting the PNA Gard162 probe (BP 530-550, FT 570, LP 591 sensitive to the Alexa Fluor 594 molecule) and the PNA FvagPNA651probe (BP 470-490, FT500, LP 516 sensitive to the Alexa Fluor 488 molecule). Twenty fields were randomly acquired in each sample. The number of bacteria was counted using *ImageJ* software (Rasband, 1997), applying automated counts and specific thresholds as indicated in a previous protocol (Labno). Biofilm assays were repeated three times on separate days.

### Bacterial Species Discrimination in Dual-Species Biofilms by qPCR

Genomic DNA (gDNA) was extracted from the dual-species biofilms, as well as from pure cultures of the 2 species under study (for the qPCR calibration curves), using the DNeasy Ultraclean microbial kit (Qiagen), following the manufacturer instructions, with minor adaptations. In brief, bacterial samples were centrifuged at 14000 rpm for 5 min, the supernatant carefully removed, and the pellet frozen at -20°C, overnight. This step increased the DNA yield up to 2-fold. The cells were lysed in a BeadBug 6 Microtube Homogenizer (Benchmark Scientific, NJ, USA) using 2×3 cycles of 30 s at 4350 rpm, and samples were kept on ice between the 2 cycles. To assess the efficiency and variability of gDNA extraction between samples, 10 µL of luciferase cDNA, obtained as described before (Magalhães et al., 2019), were added to each sample, before transferring the lysate to the spin column. gDNA was eluted in 50 µL of DNase-free water. To determine the bacterial load, a calibration curve was generated with gDNA isolated from pure bacterial cultures with concentrations ranging from 1 × 10^9^ CFU/mL to 5 × 10^6^ CFU/mL. The primers used to quantify *G. vaginalis* (Fw CCTCATGCAAAATGTGATGC; Rv CCAAAACAGAAGCACGGAAT; amplifying the locus GAVG_1017, obtained from GenBank: AP012332.1) or *F. vaginae* (Fw CCTCATGCAAAATGTGATGC; Rv CCAAAACAGAAGCACGGAAT; amplifying the locus I6G91_00565, obtained from GenBank: CP065631.1) were designed with CLC genomics workbench version 21 (QIAGEN). Primer specificity was specifically designed to differentiate these two species in this controlled *in vitro* study and was first confirmed using Primer-BLAST and then experimentally determined by qPCR. All samples, including the standard curves, were diluted 10× in DNase-free water and then 2 µL of these solutions were mixed with 8 µL of reaction buffer containing 5 µL of Xpert Fast SYBR (Grisp, Porto, Portugal), 1 µL of primers mixture (at 10 µM) and 2 µL of water. All samples were analyzed in triplicates. Non-template controls were performed to evaluate reagent contamination. To assess the efficiency of gDNA extraction, and to calibrate data between qPCR runs, a control was used by adding 2 µL of cDNA luciferase to each qPCR plate. qPCR runs were performed in a CFX96™ (Bio-Rad, CA, USA) with the following cycle parameters: 95°C for 3 min, and 40 cycles of 95°C for 5 s and 60°C for 20 s. Melt analysis was performed to ensure the absence of unspecific products and primer-dimers. PCR amplification efficiency was determined from the slope of a standard curve and efficiencies of 82% for *G. vaginalis* primers and 79% for *F. vaginae* were obtained. Bacterial load in each sample was interpolated from the averaged standard curves. This experiment was repeated three times on separate days.

## Results

### Design and *In Silico* Analysis of the *F. vaginae* PNA Probe

The alignment of the two sets of rRNA sequences gathered for the 16S and 23S rRNA evaluation revealed that the large subunit sequences presented conserved regions of potential interest (i.e. consistent among the *F. vaginae* sequences and with mismatches in the non-*F. vaginae* sequences) (**Supplementary Figure 1**). The candidate target region for probe design was then selected based on the number of target strains, the position of the mismatches in the close related strains used for the alignment, % of GC, melting temperature, and free energy. The probe was named FvagPNA651 considering the target starting position in the 23S rRNA (*E. coli* numbering).

*In silico* evaluation of *F. vaginae* probe performance was done using the TestProbe tool that searches for targets of the probe in an online database of rRNA sequences. A total of 157859 sequences, from the REF sequence collection, large subunit, 23S database (Arb-Silva) were analyzed. From those sequences, only six sequences corresponded to *F. vaginae* strains. The determination of sensitivity and specificity was done using the equations previously described by Almeida et al. (Almeida et al., 2013). The analysis of the probe resulted in a theoretical value of sensitivity of 100% and specificity of 99.9%; but it should be considered that the sensitivity value was estimated based on the very low available number of *F. vaginae* target sequences. The values of melting temperature and Gibbs free energy were also calculated *in silico*, resulting in 61.16°C and -17.71 kcal/mol, respectively.

### Optimization of Experimental Conditions of FISH Procedure

PNA-FISH procedure can be affected by several factors that will influence the fluorescence signal of the probe. Factors such as pH, dextran sulfate, probe concentration (Rocha et al., 2016), fixation and permeabilization steps (Rocha et al., 2018), as well as the time and temperature of hybridization, are important in the outcome of the FISH method. As such, we first performed pilot experiments testing different times (60 and 90 min) and temperatures (50 to 63°C) of hybridization to obtain the best signal of the probe. The optimization assays (**Supplementary Table 1**) resulted in an optimal signal-to-noise ratio at a temperature of 56°C and 60 min, which was the selected temperature for the determination of the probe analytical sensitivity and specificity.

### Determination of *F. vaginae* Probe Analytical Sensitivity and Specificity

**Figure 1:**
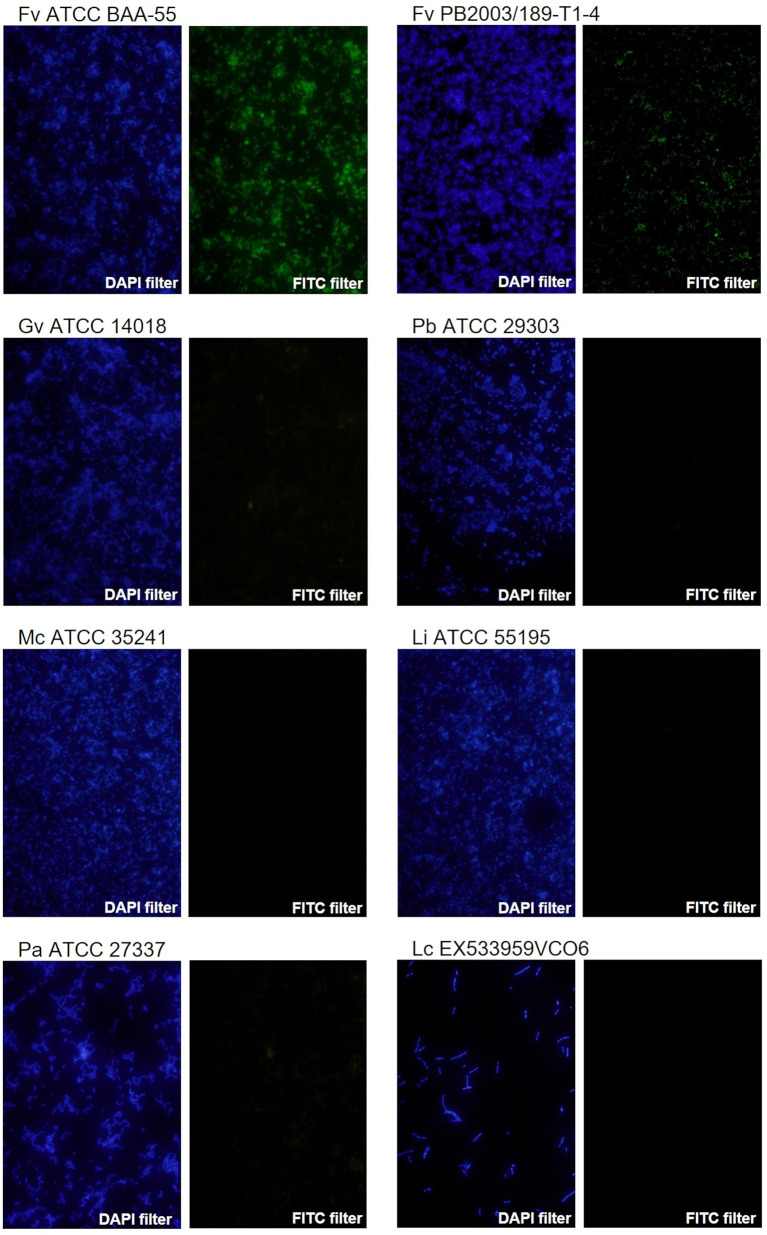
Examples of fluorescence microscopy results using the novel PNA probe with *F. vaginae* strains and other BV-related species. *F. vaginae* ATCC BAA-55 (good hybridization), *F. vaginae* PB2003/189-T1-4 (poor hybridization), *G. vaginalis* ATCC 14018, *P. bivia* ATCC 29303, *M. curtisii* ATCC 35241, *L. iners* ATCC 55195, *P. anaerobius* ATCC 27337 and *L. crispatus* EX533959VCO6 (absence of hybridization). For each strain/species an image of DAPI staining (DAPI filter) and the correspondent signal of the probe (FITC filter) is shown. The images were acquired with a magnification of 400×.

*F. vaginae* probe analytical sensitivity was determined using 24 different isolates of *F. vaginae*. As described in **Table 1**, the results of hybridization were qualitatively classified into four levels: absence (-), poor (+), moderate (++), and good (+++) hybridization. All tested *F. vaginae* isolates showed hybridization with the probe, although with different efficiencies, resulting in a value of analytical sensitivity of 100%. For the determination of analytical specificity, 40 different bacterial species associated with BV or with vaginal microbiota were used. Under the tested conditions, no hybridization was detected, which result in an analytical specificity of 100% (**Table 2**). **Figure 1** presents examples of the hybridization results for *F. vaginae* strains with either good or poor hybridization, as well as for some of the most common BV-associated bacteria, as well as *L. crispatus* which is typically associated with optimal vaginal microbiota. Results from the other tested species are available in **Supplementary Figure 2**.

**Table 1:** Hybridization of *F. vaginae* probe with different strains of *F. vaginae* for determination of analytical sensitivity.

Strain	Reference	Hybridization result
*Fannyhessea vaginae*	ACS-043-V-Col2	++
*Fannyhessea vaginae*	ATCC BAA-55	+++
*Fannyhessea vaginae*	BVS064	++
*Fannyhessea vaginae*	BVS065	++
*Fannyhessea vaginae*	BVS067	++
*Fannyhessea vaginae*	BVS069	++
*Fannyhessea vaginae*	CCUG 42099	+++
*Fannyhessea vaginae*	CCUG 44116	++
*Fannyhessea vaginae*	FB010-06	+++
*Fannyhessea vaginae*	FB101-3C	++
*Fannyhessea vaginae*	FB106b	++
*Fannyhessea vaginae*	FB106B	++
*Fannyhessea vaginae*	FB106C	++
*Fannyhessea vaginae*	FB130-CNAB-2aD	++
*Fannyhessea vaginae*	FB145-BA-14A	+++
*Fannyhessea vaginae*	FB158-CNA-2C	+++
*Fannyhessea vaginae*	FB160-CNAB-7	++
*Fannyhessea vaginae*	FB160-CNAB-7A	++
*Fannyhessea vaginae*	PB2003/009-T1-4	++
*Fannyhessea vaginae*	PB2003/017-T1-2	++
*Fannyhessea vaginae*	PB2003/189-T1-4	+
*Fannyhessea vaginae*	VMF0907COL23	+++
*Fannyhessea vaginae*	VMF0914COL13	++
*Fannyhessea vaginae*	VMF0914COL43	++

Hybridization results were evaluated qualitatively according to the classification: (-) Absence of hybridization; (+) Poor hybridization; (++) Moderate hybridization; (+++) Good hybridization.

**Table 2:** Hybridization of *F. vaginae* probe with different species for determination of analytical specificity.

Species	Reference	Hybridization result
*Acinetobacter baumanii*	CCUG 59798	–
*Actinomyces neuii*	UM067	-*
*Actinomyces urogenitalis*	CCUG 44038	–
*Aerococcus christensenii*	CCUG 28826	-*
*Bacillus firmus*	UM034	–
*Bifidobacterium bifidum*	CCUG 59492	-*
*Brevibacterium ravenspurgense*	CCUG 42923	-*
*Campylobacter ureolyticus*	CCUG 44295	-*
*Corynebacterium tuscaniense*	UM137	–
*Enterococcus faecalis*	UM035	-*
*Escherichia coli*	UM056	–
*Gardnerella leopoldii*	UM034	-*
*Gardnerella piotii*	UM035	-*
*Gardnerella swidsinskii*	UM094	-*
*Gardnerella vaginalis*	ATCC 14018	-*
*Gemella haemolysans*	UM034	-*
*Lactobacillus crispatus*	EX533959VCO6	–
*Lactobacillus gasseri*	ATCC 9857	-*
*Lactobacillus iners*	ATCC 55195	–
*Lactobacillus rhamnosus*	CECT 288	-*
*Lactobacillus vaginalis*	UM062	–
*Megasphaera micronuciformis*	CCUG 45952T	-*
*Mobiluncus curtisii*	ATCC 35241	–
*Mobiluncus mulieris*	ATCC 35239	-*
*Mycoplasma hominis*	UM054	-*
*Neisseria gonorrhoeae*	CCUG 13281	-*
*Nosocomiicoccus ampullae*	UM121	-*
*Peptostreptococcus anaerobius*	ATCC 27337	-*
*Porphyromonas asaccharolytica*	CCUG 7834T	–
*Prevotella bivia*	ATCC 29303	–
*Propionibacterium acnes*	UM034	-*
*Shigella* spp.	UM137	–
*Sneathia sanguinegens*	CCUG 66076	-*
*Staphylococcus epidermidis*	UM066	-*
*Staphylococcus haemolyticus*	UM066	–
*Staphylococcus hominis*	UM224	-*
*Staphylococcus saprophyticus*	UM121	–
*Staphylococcus simulans*	UM059	–
*Streptococcus agalactiae*	UM035	–
*Veillonella parvula*	CCUG 59474	-*

Hybridization results were evaluated qualitatively according to the classification: (-) Absence of hybridization; (+) Poor hybridization; (++) Moderate hybridization; (+++) Good hybridization. *These species showed some autofluorescence signal detected in the FITC filter.

### Detection of *G. vaginalis* and *F. vaginae* in Dual-Species Biofilms

**Figure 2:**
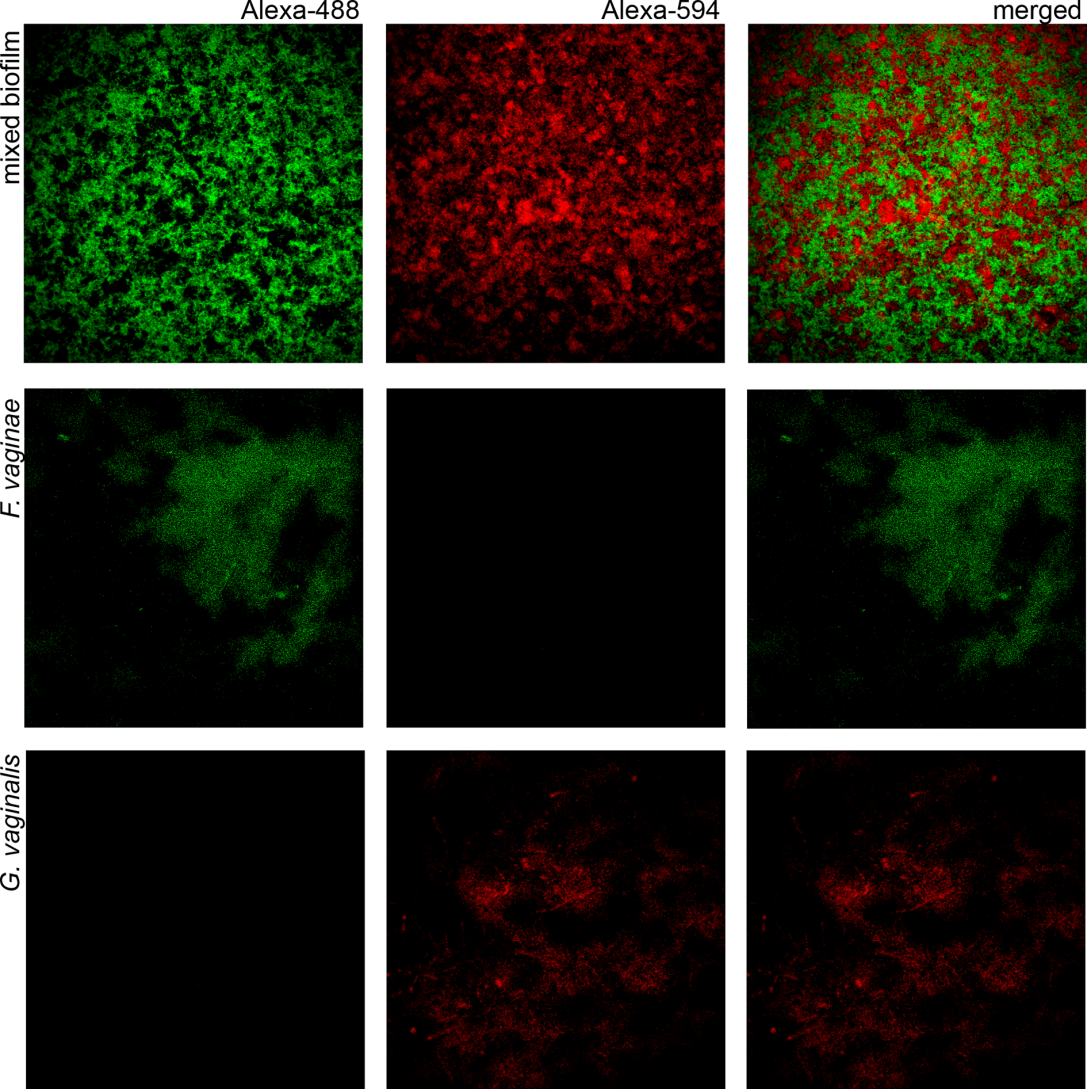
Confocal Laser Scanning Microscopy images of single- and dual-species biofilms hybridization with Gard162 and FvagPNA651 probes. Gard162 probe detected *G. vaginalis* 24 h single-species biofilm and *F. vaginae* probe detected *F. vaginae* 24 h single-species biofilm. The probes were able to detect *G. vaginalis* (red) and *F. vaginae* (green) on 24 h dual-species biofilm. The images were acquired using 40× objective.

Since BV development has been strongly associated with *Gardnerella* and *F. vaginae* biofilms (Swidsinski et al., 2005; Hardy et al., 2016), we also tested if the probe could be used in a multiplex assay, to discriminate species within a biofilm. A mixed biofilm of *G. vaginalis* and *F. vaginae* was grown and the presence of both species was assessed using our novel probe and a previously developed *Gardnerella* probe (Machado et al., 2013). As shown in **Figure 2**, both probes were able to detect the respective species in mono- and dual-species biofilms. We further assessed if this method could be used to estimate the abundance of *F. vaginae* in this complex biofilm structure. PNA-FISH image quantification resulted in *F. vaginae* abundance of 50.0 ± 7.8% but when the same biofilms were quantified by qPCR, *F. vaginae* abundance was slightly lower, around 39.1 ± 3.8%.

## Discussion

In clinical settings, BV is most commonly diagnosed using the highly subjective Amsel’s criteria (Forsum et al., 2005). Conversely, laboratory diagnosis is often based on microscopic observation of vaginal fluid specimens which are Gram-stained, to determine the Nugent Score (Nugent et al., 1991). Both methods are neither highly specific nor sensitive (Forsum et al., 2005) and the concordance between the two methods varies between 80% to 90% (Livengood, 2009). Due to antibiotic resistance in BV and its impact on BV recurrence (Bradshaw et al., 2006a; Sobel et al., 2019), the necessity of developing more reliable diagnostic methods have emerged (Cartwright et al., 2012; Hilbert et al., 2016; Gaydos et al., 2017; Schwebke et al., 2020). Herein, we designed a new PNA probe specific for *F. vaginae* that can be used in combination with a *Gardnerella* probe for a highly accurate BV diagnosis in laboratory settings. The theoretical evaluation of the new probe showed a sensitivity and specificity of 100% and 99.9%, respectively. This excellent performance was confirmed experimentally using 24 strains of *F. vaginae* and 40 other culturable species associated with the vaginal microbiota. Furthermore, our probe was also efficient in discriminating species in a multispecies biofilm.

The study of BV biofilms using FISH methodology has been widely developed since the first study conducted by Swidsinskii and colleagues that showed the presence of a biofilm in the vaginal epithelium, composed of *Gardnerella* spp. and *F. vaginae*, using DNA-probes specific for these species (Swidsinski et al., 2005). Despite being more affordable, DNA-FISH probes often present some low permeability efficiency and affinity, resulting in weaker fluorescence signals. PNA probes overcome some of the disadvantages of DNA probes (Singh et al., 2020). PNA probes are synthetic nucleic acids analogs, where the negatively charged backbone characteristic of DNA structure is replaced by an uncharged polyamide backbone, formed by repetitive units of N-(2-aminoethyl) glycine (Stender et al., 2002; Singh et al., 2020). The synthetic backbone and consequently the lack of electrostatic repulsion, provides PNA probes unique hybridization characteristics such as improved thermal stability, allowing a stronger binding, higher specificity to complementary sequences and more rapid hybridization kinetics comparing to the traditional DNA probes (Perry-O’Keefe et al., 2001; Stender et al., 2002; Shakeel et al., 2006). PNA probes also hybridize under low salt concentrations which is ideal for targeting nucleic acids with a high degree of secondary structures, as the absence of salts destabilizes the secondary structures (Perry-O’Keefe et al., 2001; Stender et al., 2002; Singh et al., 2020). The relative hydrophobic character of PNA probes allows the easy diffusion of the probe through the hydrophobic cell wall of fixed bacteria and yeasts (Stender et al., 2014). Furthermore, the unnatural backbone provides PNA probes resistance to the degradation by enzymes, such as nucleases and proteases (Stender et al., 2002; Singh et al., 2020). All these characteristics have given PNA a remarkable advantage over the use of DNA probes (Singh et al., 2020), and nowadays they are widely used in FISH methodology as means to improve its efficiency. The *F. vaginae* PNA-probe (AtoITM1) previously developed by Hardy and colleagues showed a sensitivity of 66.7% and specificity of 89.4%. However, their probe efficiency determination was not assessed against a large panel of pure cultures clinical isolates, as in this study, but instead by comparing PNA-FISH data with PCR data obtained by analyzing vaginal samples (Hardy et al., 2015), and as such, a direct comparison of probes efficacy is not possible.

One disadvantage of PNA-FISH detection, as compared with qPCR, is related to the sampling process and the heterogeneity of biofilms (Lopes et al., 2018). Furthermore, PNA-FISH does not allow high-throughput analysis, becoming more time consuming. Herein, we analyzed 20 images per biofilm. Although this is a significant number of images, it only represents a fraction of the biofilm. Conversely, qPCR data is obtained by homogenizing the whole biofilm and is more likely to be quantitatively accurate.

Overall, this work demonstrates an improved alternative for the detection of *F. vaginae* in BV biofilms, with very high specificity and sensitivity. Taking into consideration that *F. vaginae* and *Gardnerella* co-culture has been considered the most specific marker for BV diagnosis (Menard et al., 2008), our multiplex approach might be a robust alternative for an accurate BV diagnosis, however, this needs to be determined in the future, by using clinical samples of women with BV. Furthermore, since this method is based on PNA-FISH methodology, it will also significantly contribute to other research studies that aim to study *in situ* BV biofilm structure, a unique advantage that non-FISH molecular methods lack.

## Data Availability Statement

The original contributions presented in the study are included in the article/**Supplementary Material**. Further inquiries can be directed to the corresponding author.

## Author Contributions

CA, CM, and NC designed the experiments. LS and CA performed the *in silico* design of the PNA probe. LS performed the PNA-FISH hybridization experiments. LS and NC performed the biofilm/CLSM experiments. JC and AF performed the PNA-FISH/qPCR comparison experiments. LS and NC drafted the manuscript. All authors contributed to the article and approved the submitted version.

## Funding

This work was funded by the National Institute of Allergy and Infectious Diseases (R01AI146065-01A1). It was also partially funded by the Portuguese Foundation for Science and Technology (FCT), under the scope of the strategic funding of unit (UIDB/04469/2020).

## Conflict of Interest

CM has received research grant support from Lupin Pharmaceuticals, is a consultant for Lupin Pharmaceuticals and BioFire Diagnostics, and has received honoraria from Elsevier, Abbott Molecular, Cepheid, Becton Dickinson, Roche Diagnostics, and Lupin. She is currently on the scientific advisory board for Roche and PhagoMed.

The remaining authors declare that the research was conducted in the absence of any commercial or financial relationships that could be construed as a potential conflict of interest.

## Publisher’s Note

All claims expressed in this article are solely those of the authors and do not necessarily represent those of their affiliated organizations, or those of the publisher, the editors and the reviewers. Any product that may be evaluated in this article, or claim that may be made by its manufacturer, is not guaranteed or endorsed by the publisher.
