# Supplementary material for: A New PNA-FISH Probe Targeting Fannyhessea vaginae
Source: Front Cell Infect Microbiol. 2021 Nov 18;11:779376. doi: 10.3389/fcimb.2021.779376 (PMC8637528; doi:10.3389/fcimb.2021.779376)

Supplementary Material

Supplementary Table 1. Results of hybridization of *F. vaginae* PNA probe with the strain *F. vaginae* ATCC BAA-55 for the different temperatures and times of hybridization tested for optimization

Temperature (˚C)	Time (min)	Hybridization results
**50**	60	++
	90	++
**53**	60	++
	90	++
**56**	60	+++
	90	++
**58**	60	++
	90	++
**60**	60	++
	90	+
**63**	60	+
	90	+

Hybridization results were evaluated qualitatively according to the classification: (-) Absence of hybridization; (+) Poor hybridization; (++) Moderate hybridization; (+++) Good hybridization.

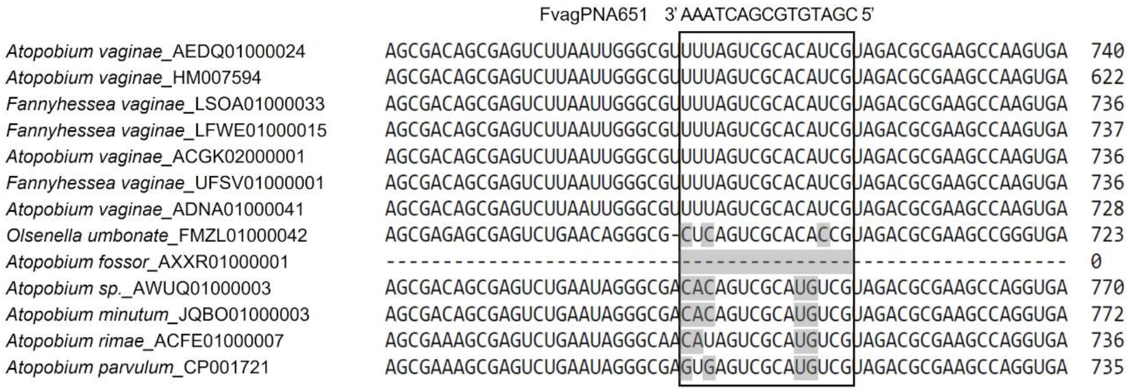

Supplementary Figure 1. Example of alignment of 23S rRNA sequences from species of interest and non-interest from Arb-Silva database. The indicated region corresponds to the sequence of the new *F. vaginae* PNA probe and mismatches with the non-interest sequences are highlighted. The complementary sequence of the probe is shown above.

Supplementary Figure 2. The following data presents the fluorescence microscopy results of *F. vaginae* PNA probe hybridization with all the tested species for sensitivity (*F. vaginae* strains) and specificity (other BV-related species) determination. The images were acquired using DAPI filter (top image, blue) and FITC filter (bottom image, green) sensitive to the Alexa fluor 488, with a magnification of 400x.

Fannyhessea vaginae **ACS-043-V-Col2**

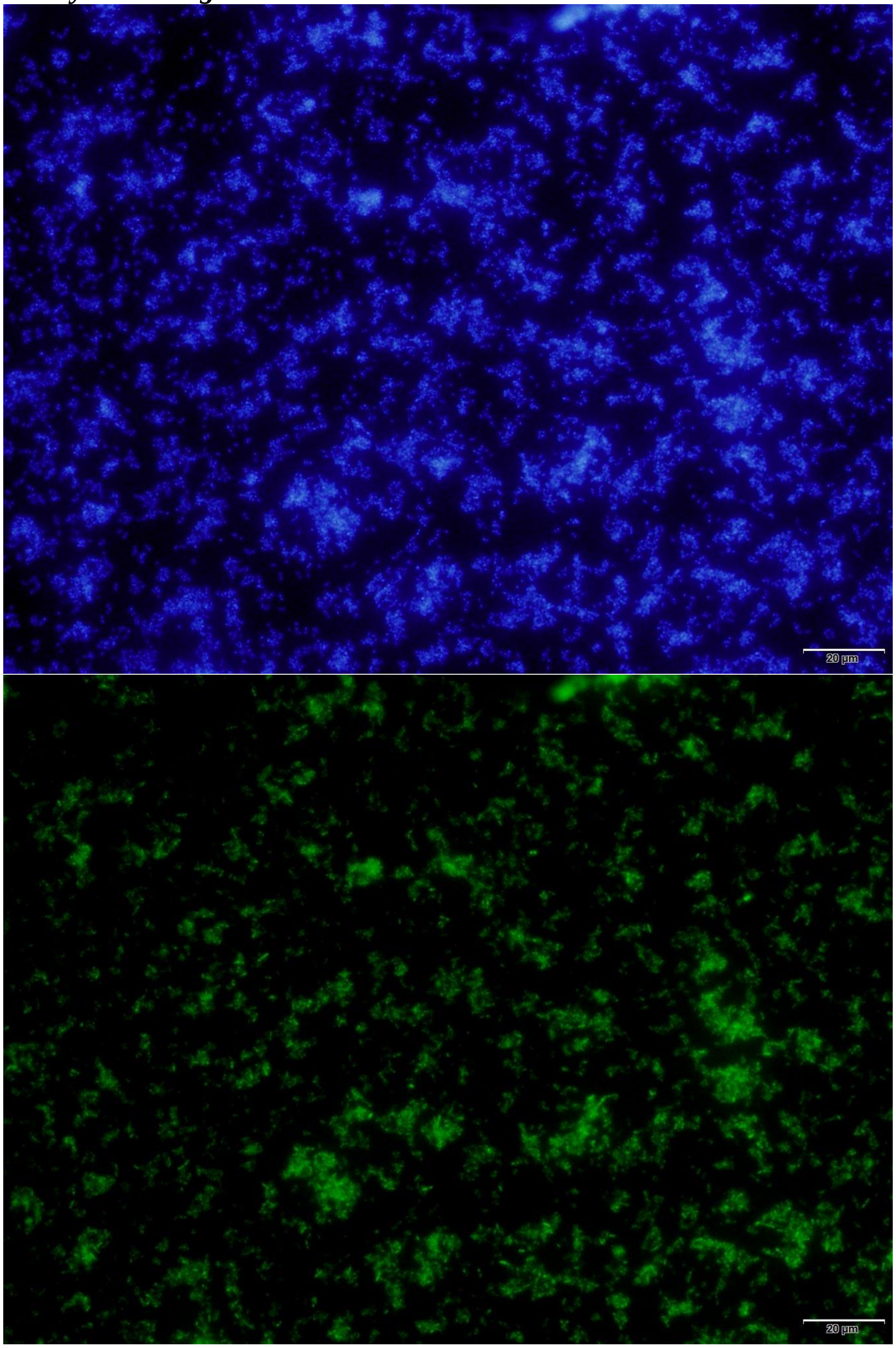

Fannyhessea vaginae **BVS064**

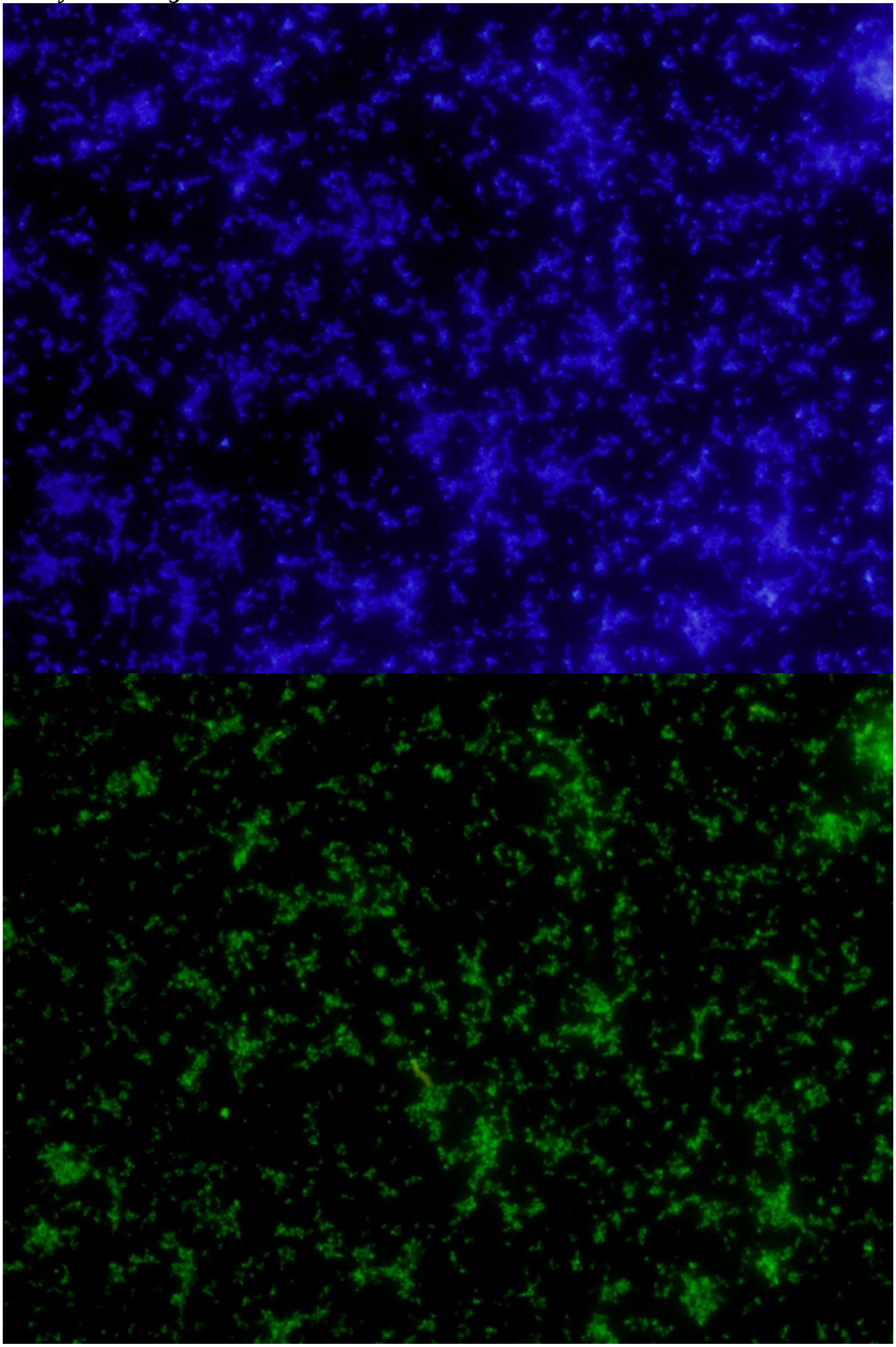

***Fannyhessea vaginae* BVS065**

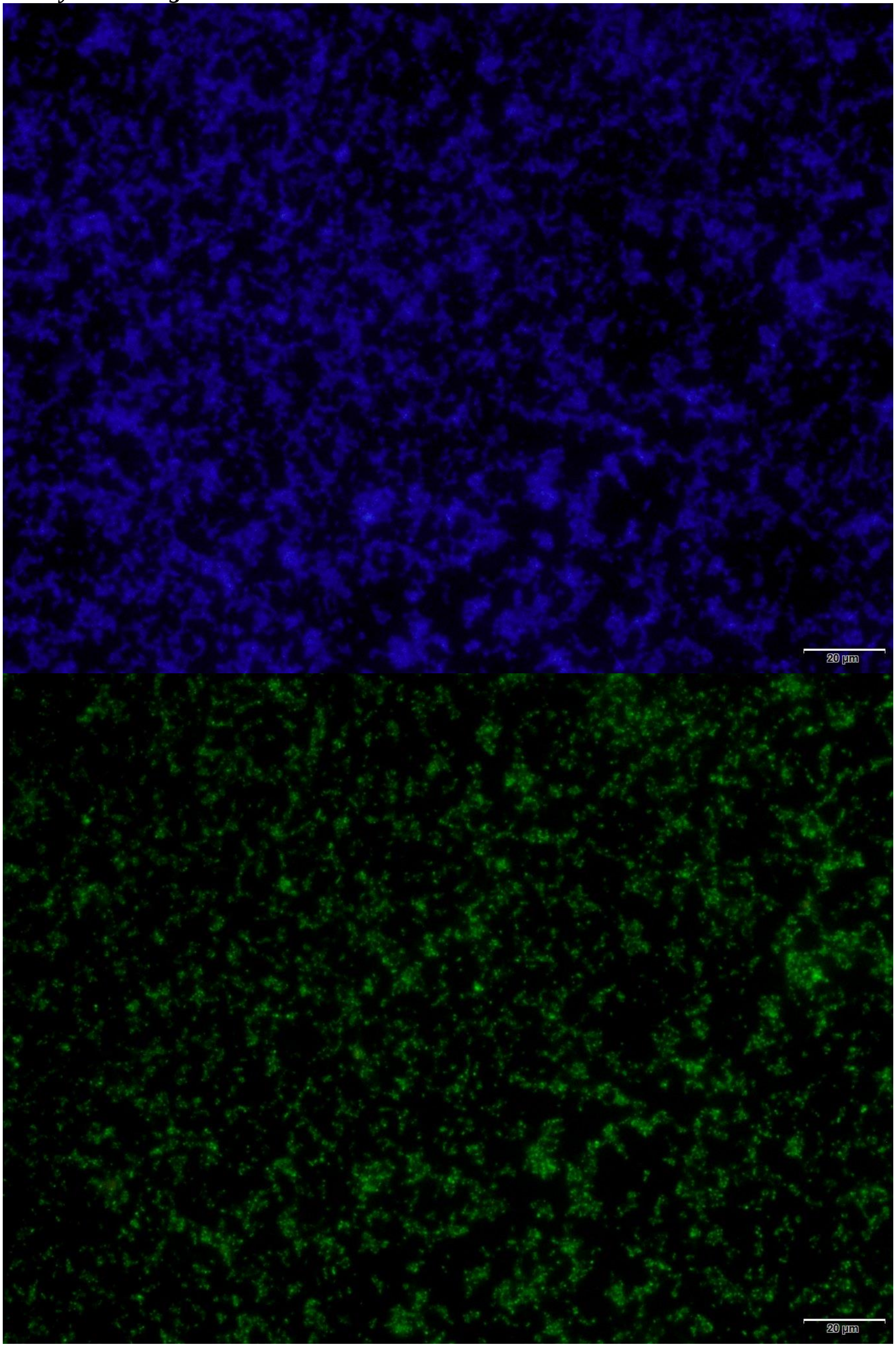

***Fannyhessea vaginae* BVS067**

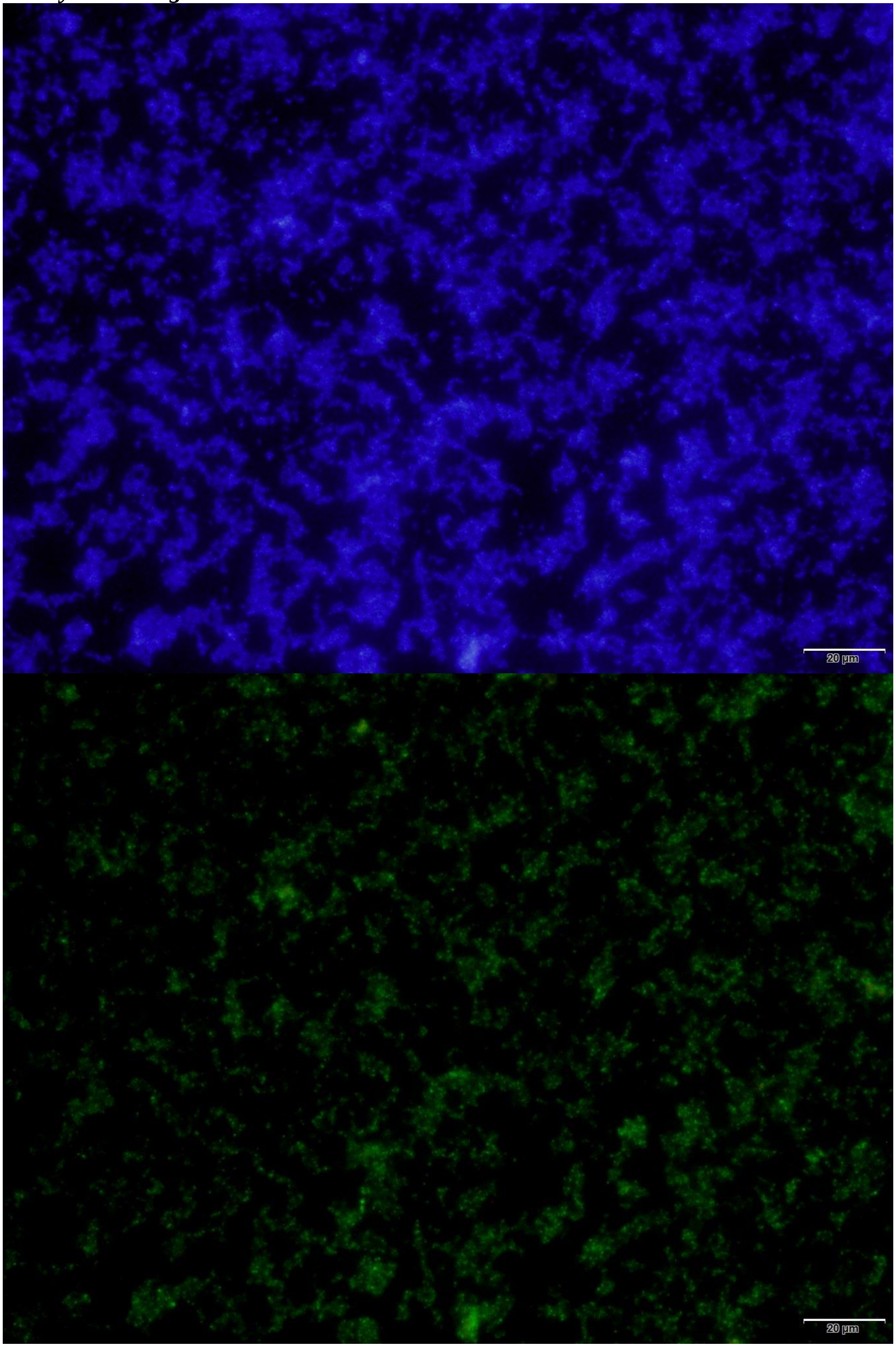

***Fannyhessea vaginae* BVS069**

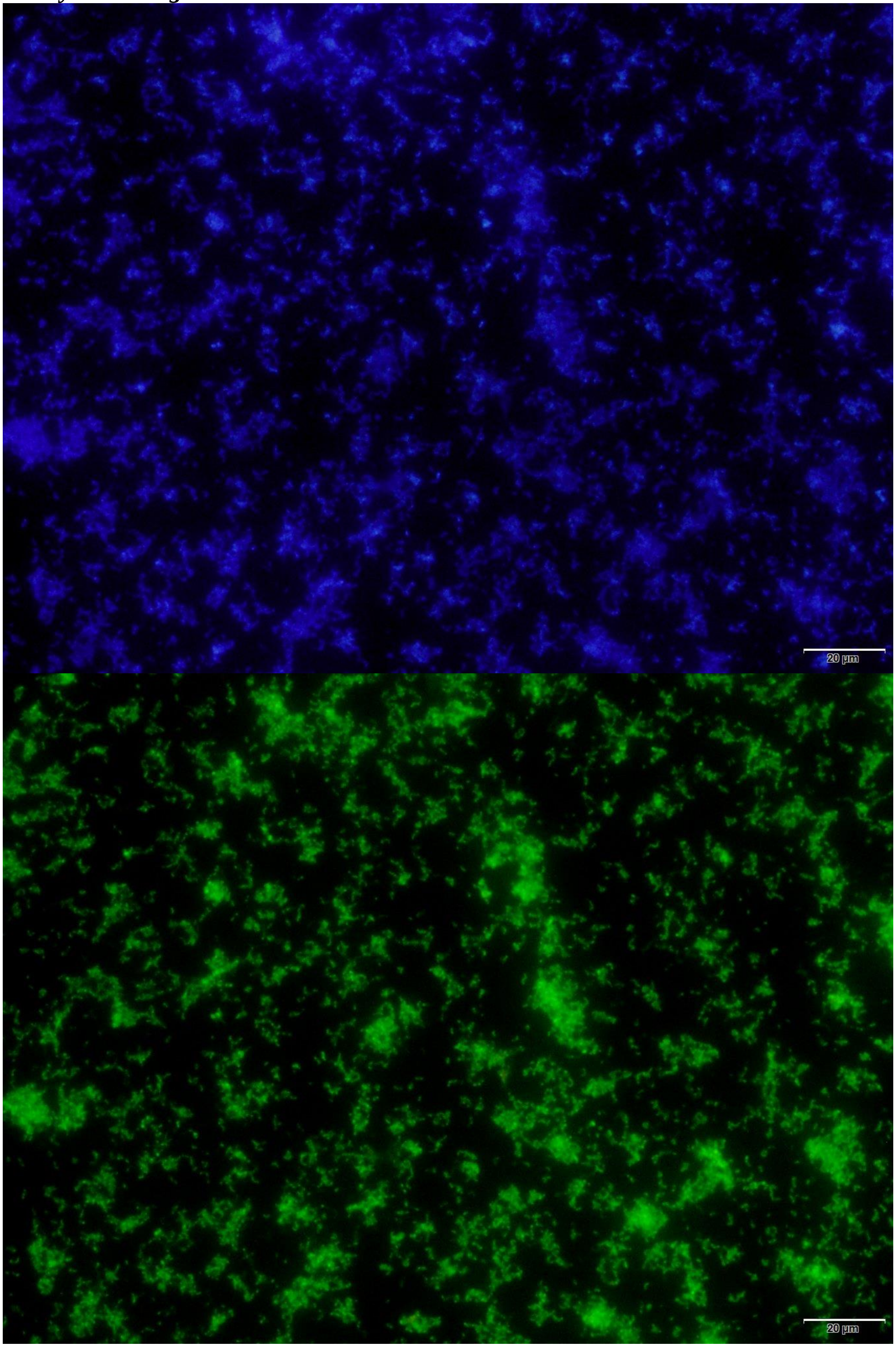

Fannyhessea vaginae **CCUG 42099**

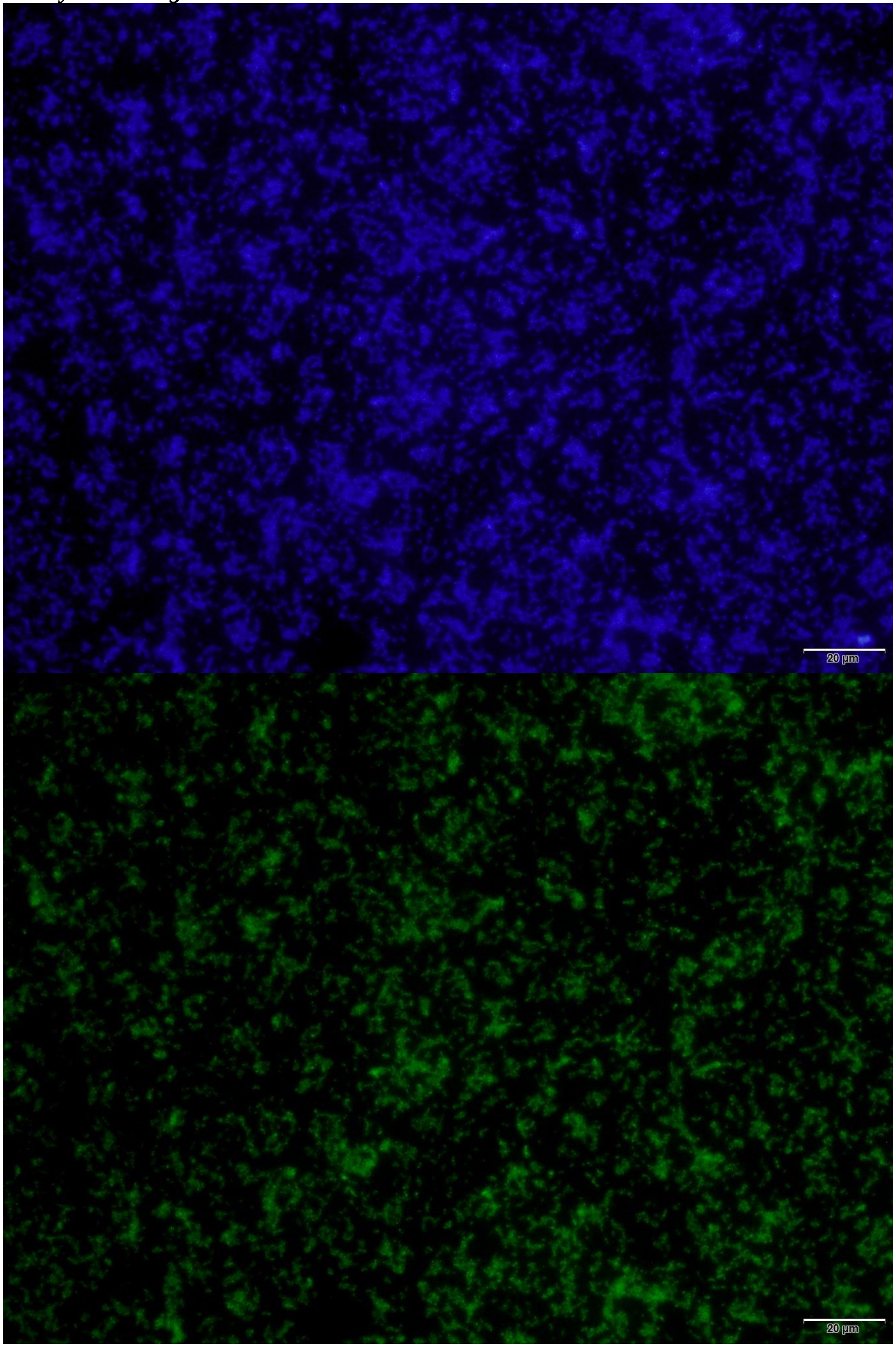

***Fannyhessea vaginae* CCUG 44116**

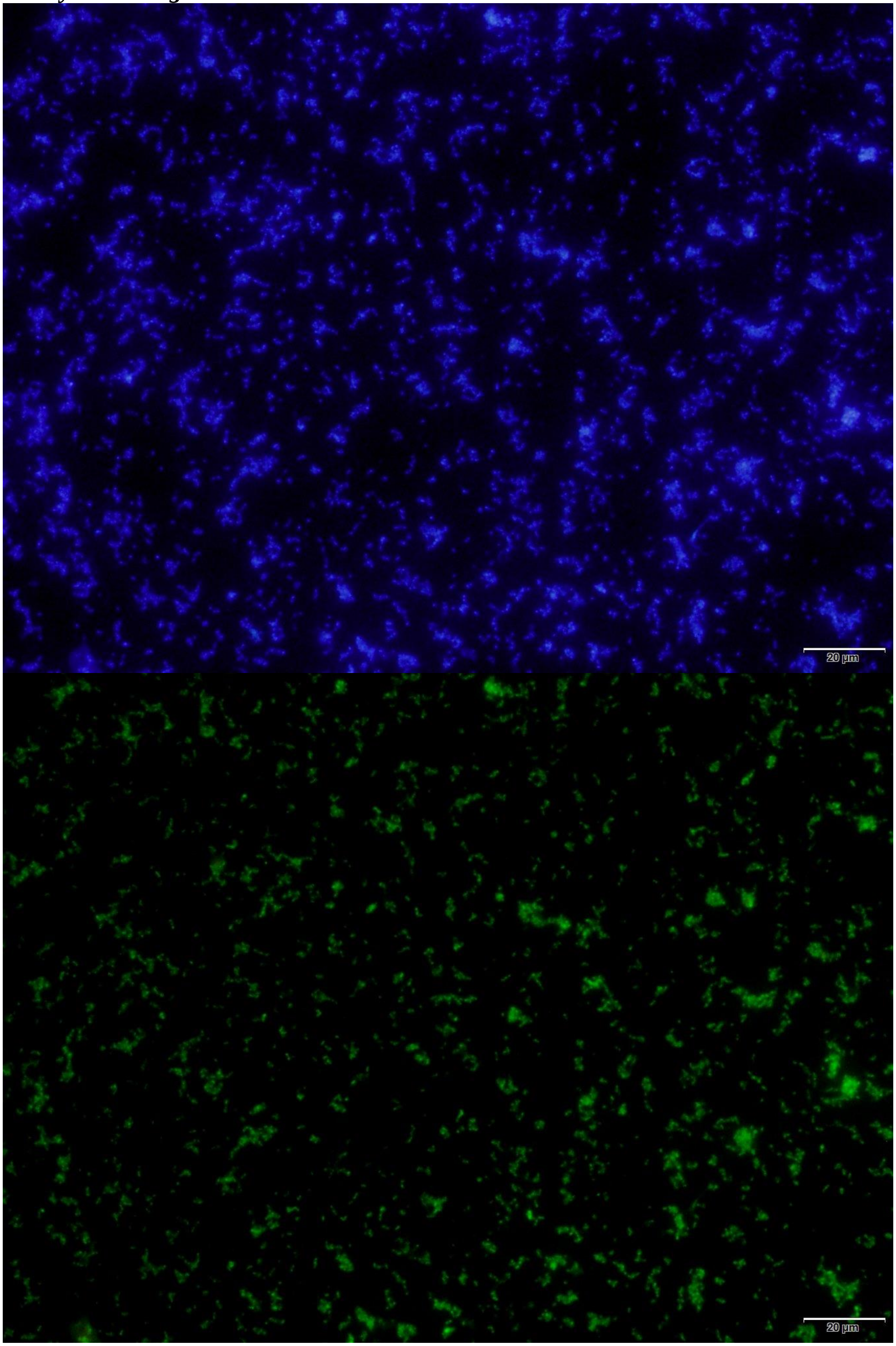

***Fannyhessea vaginae* FB010-06**

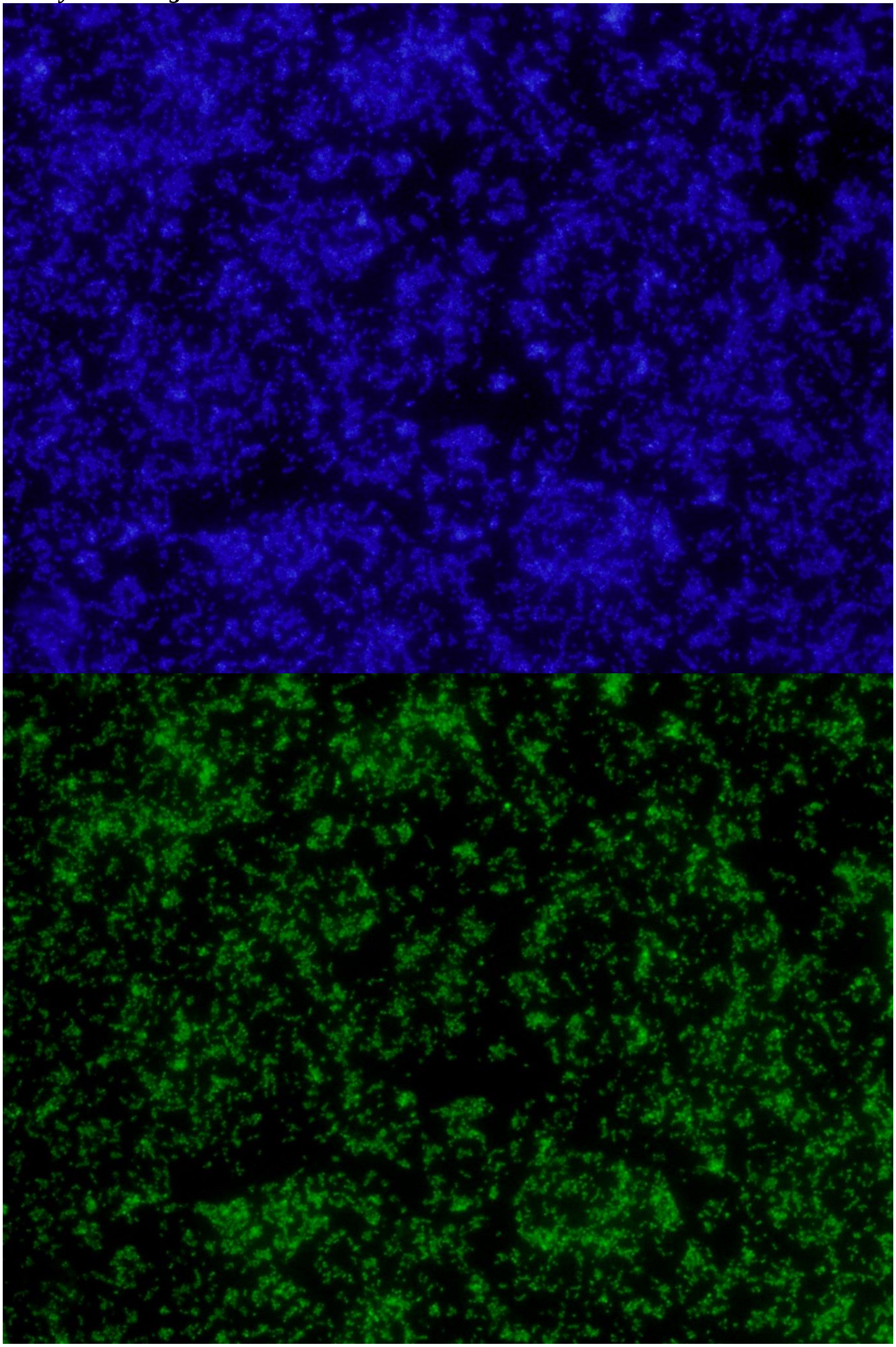

***Fannyhessea vaginae* FB101-3C**

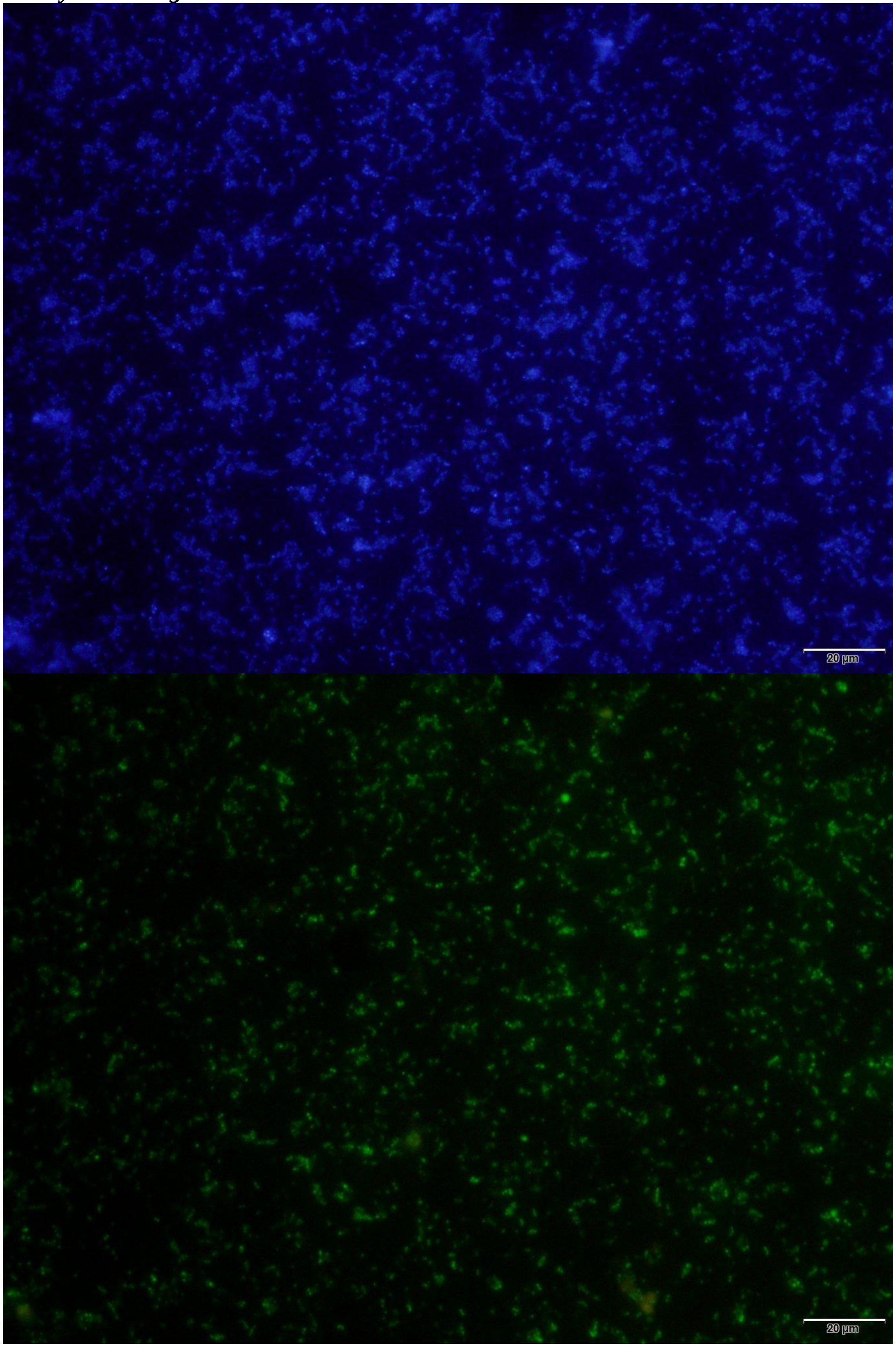

***Fannyhessea vaginae* FB106b**

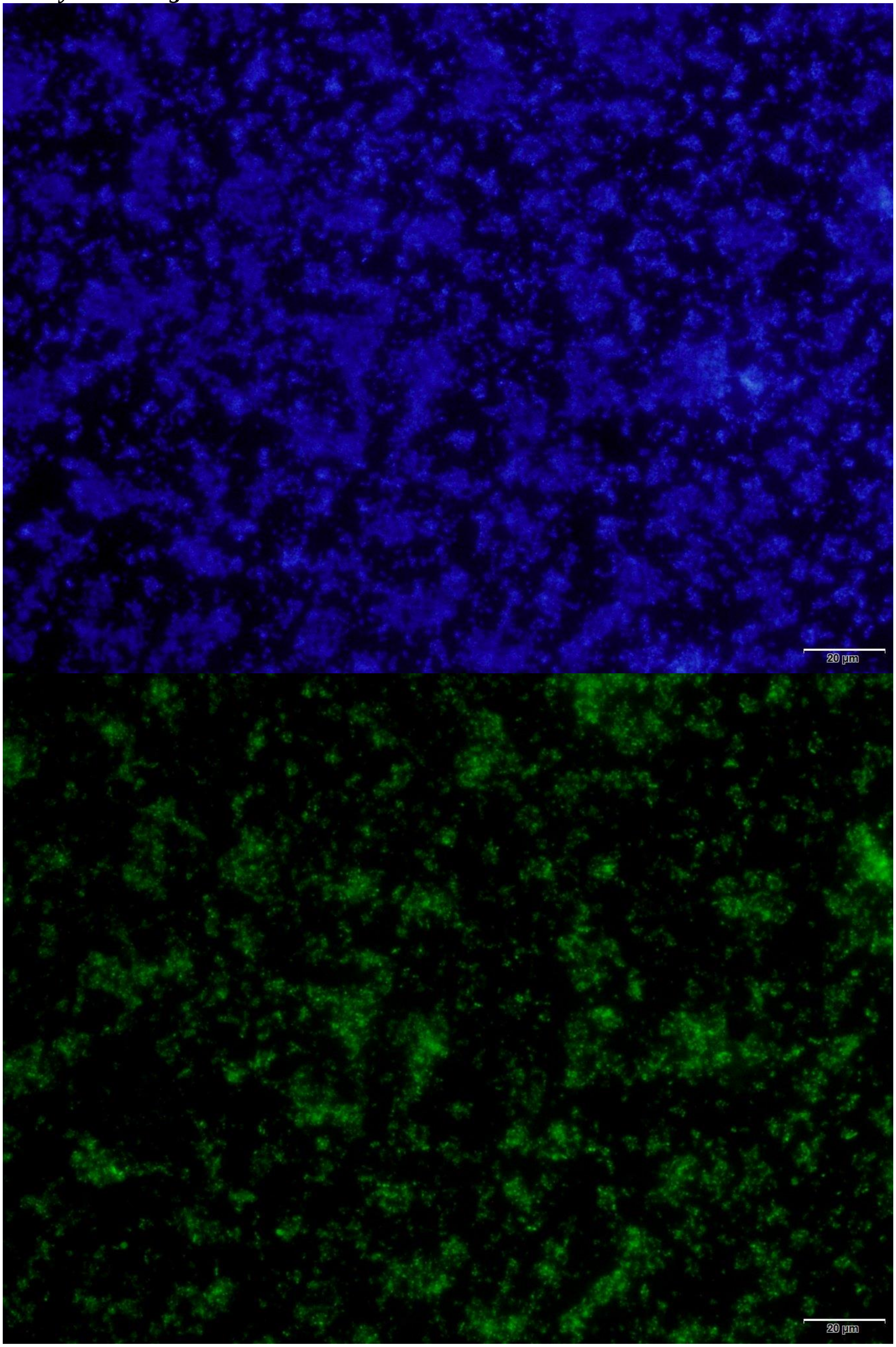

***Fannyhessea vaginae* FB106B**

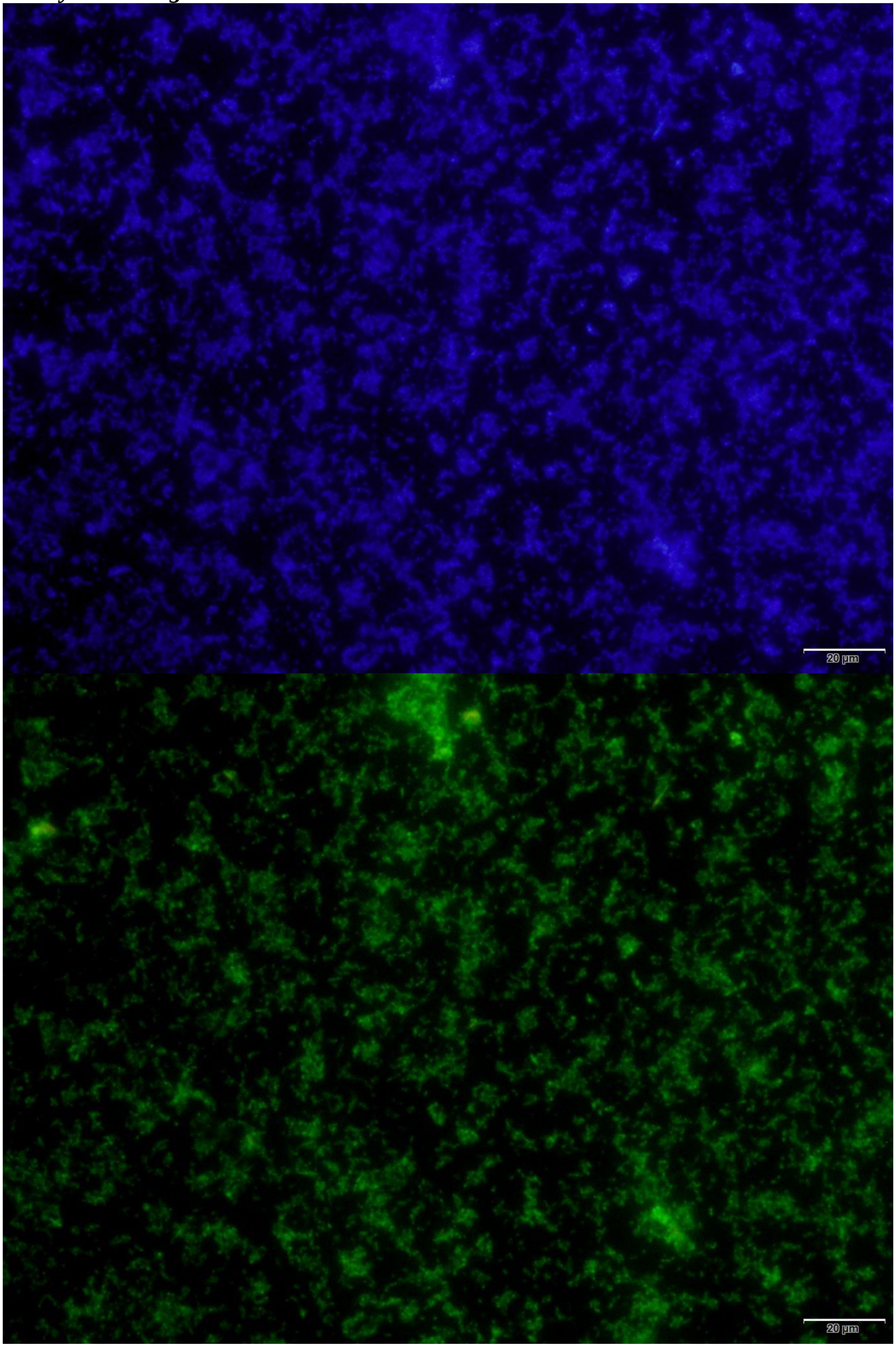

***Fannyhessea vaginae* FB106C**

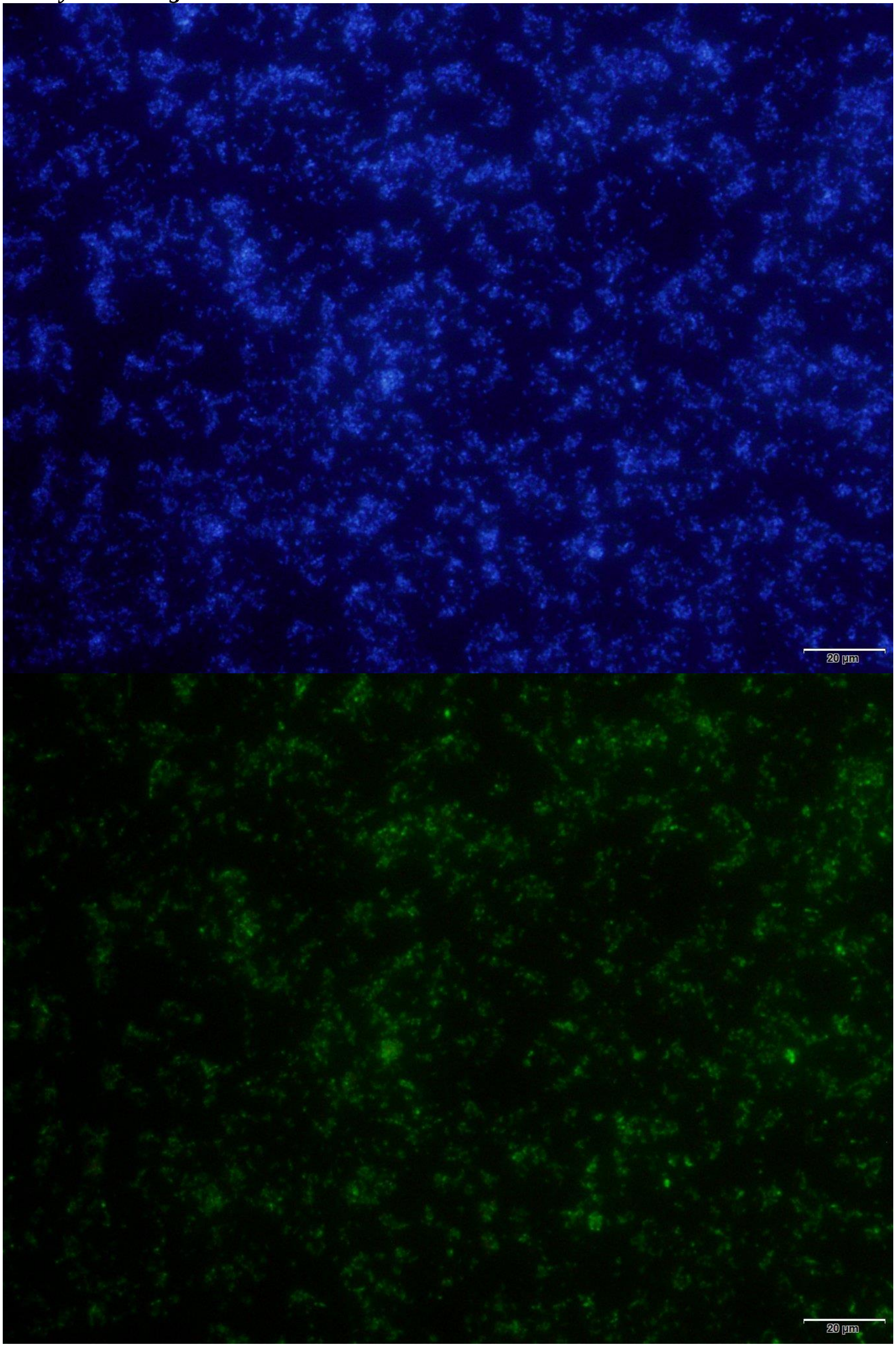

***Fannyhessea vaginae* FB130-CNAB-2aD**

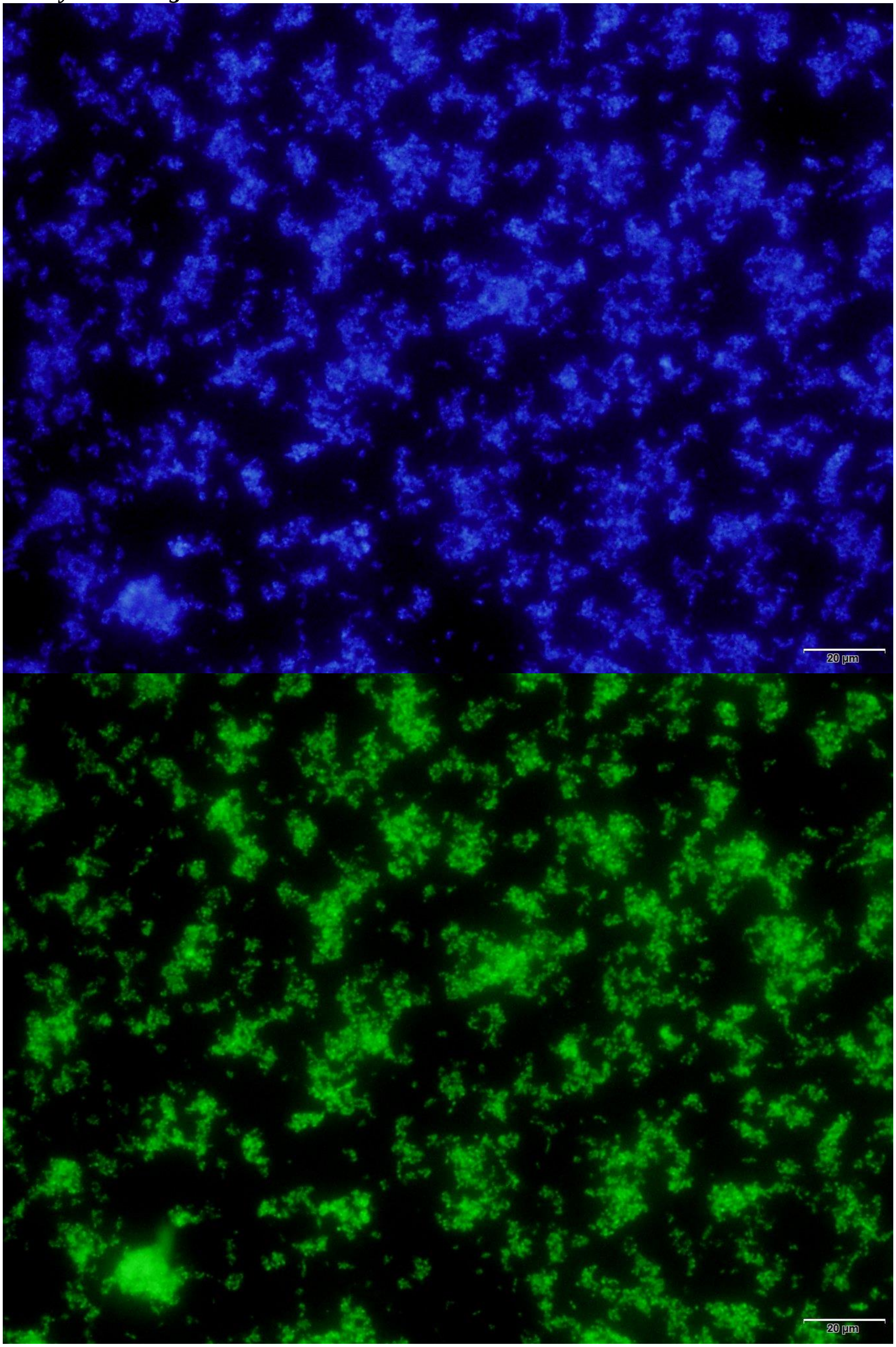

***Fannyhessea vaginae* FB145-BA-14A**

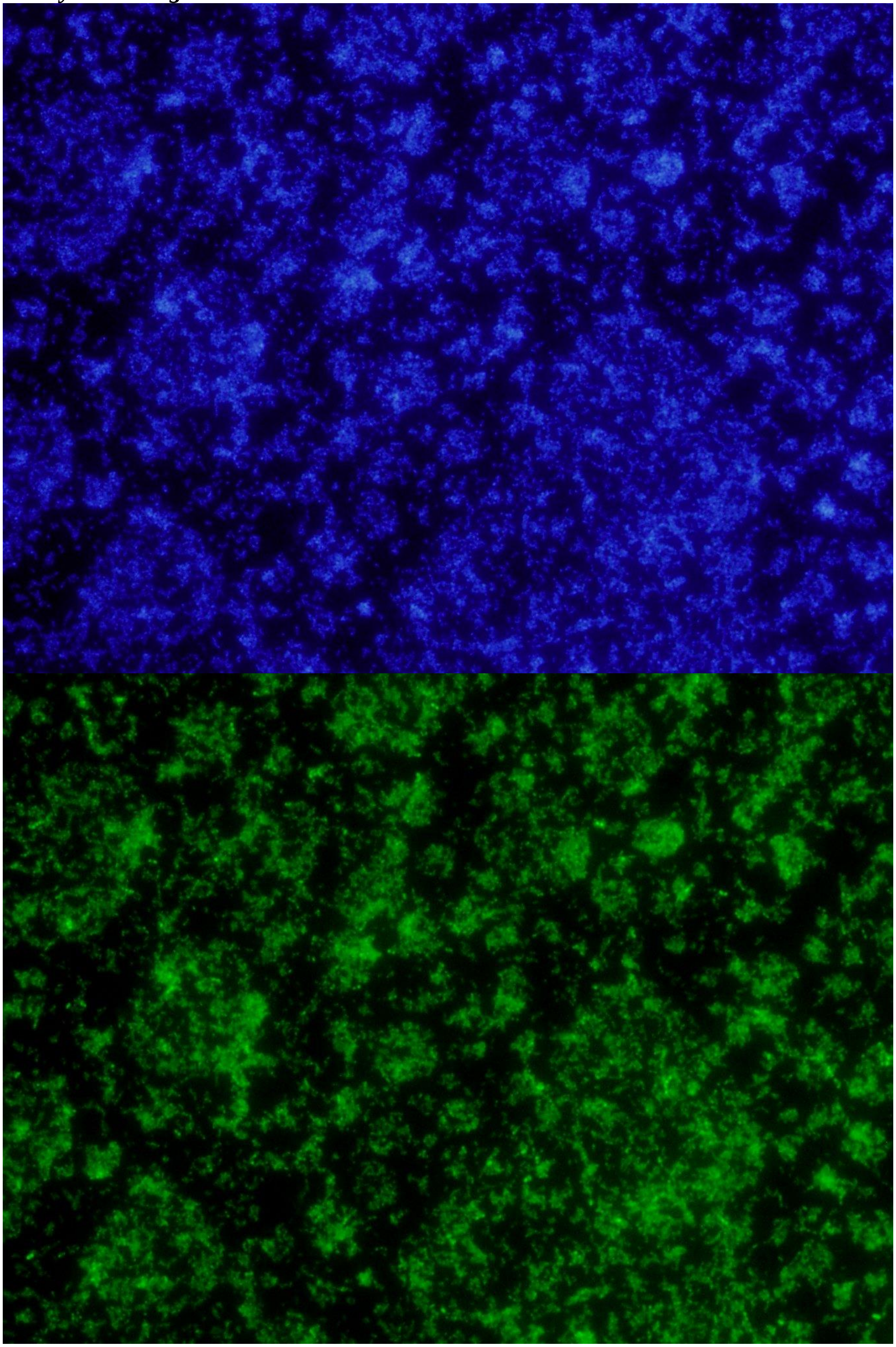

***Fannyhessea vaginae* FB158-CNA-2C**

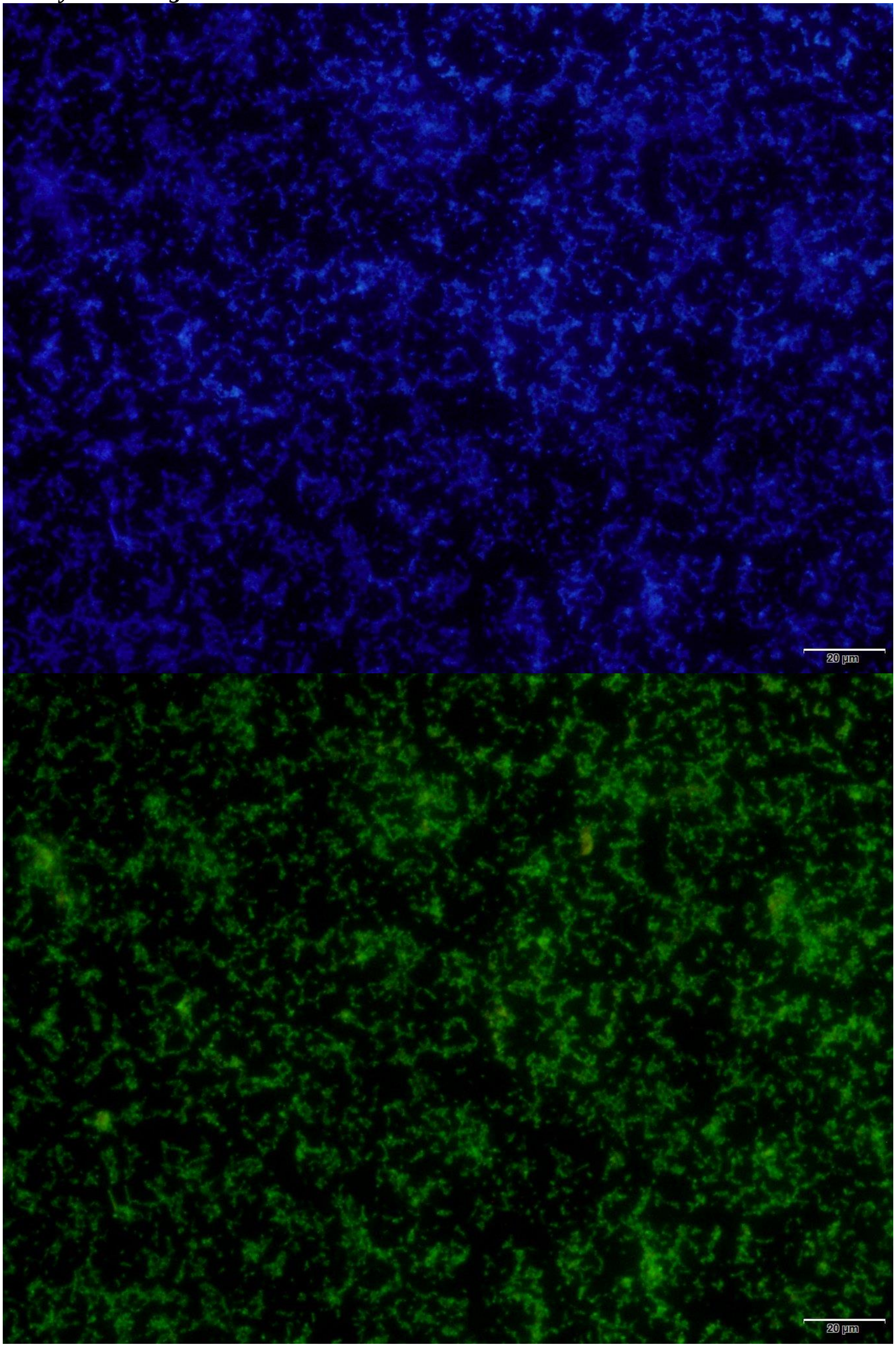

***Fannyhessea vaginae* FB160-CNAB-7**

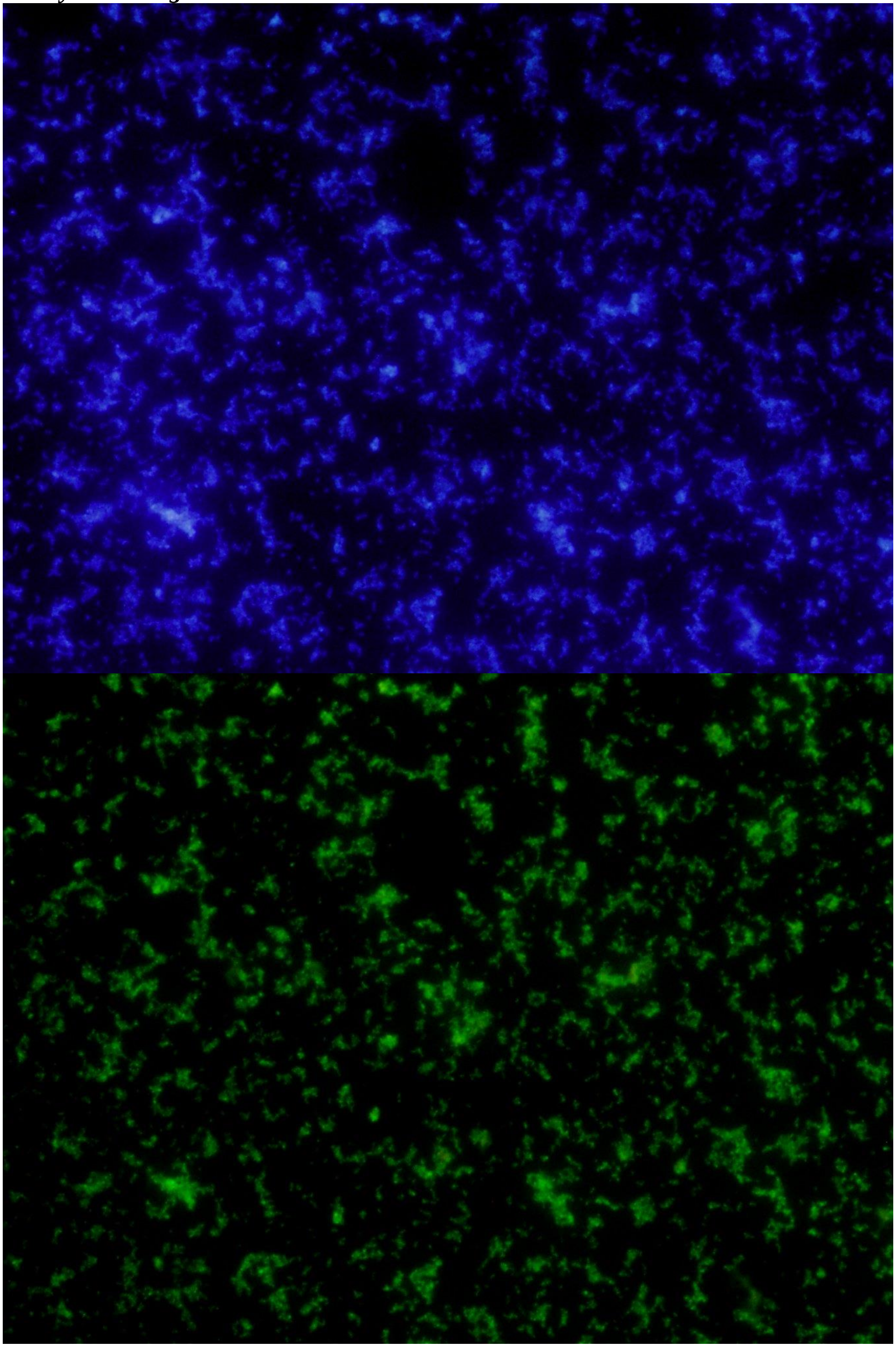

***Fannyhessea vaginae* FB160-CNAB-7A**

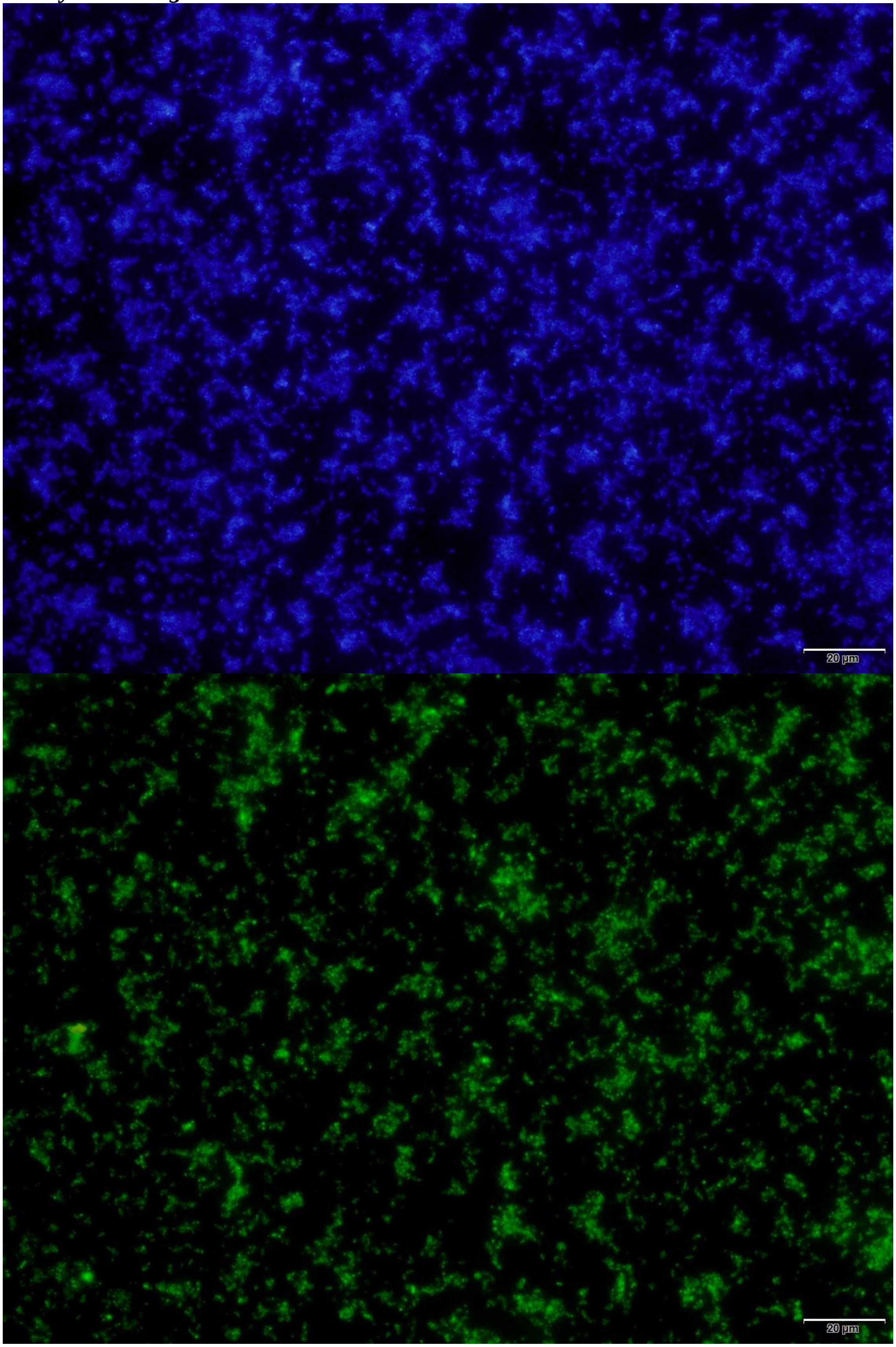

***Fannyhessea vaginae* PB2003/009-T1-4**

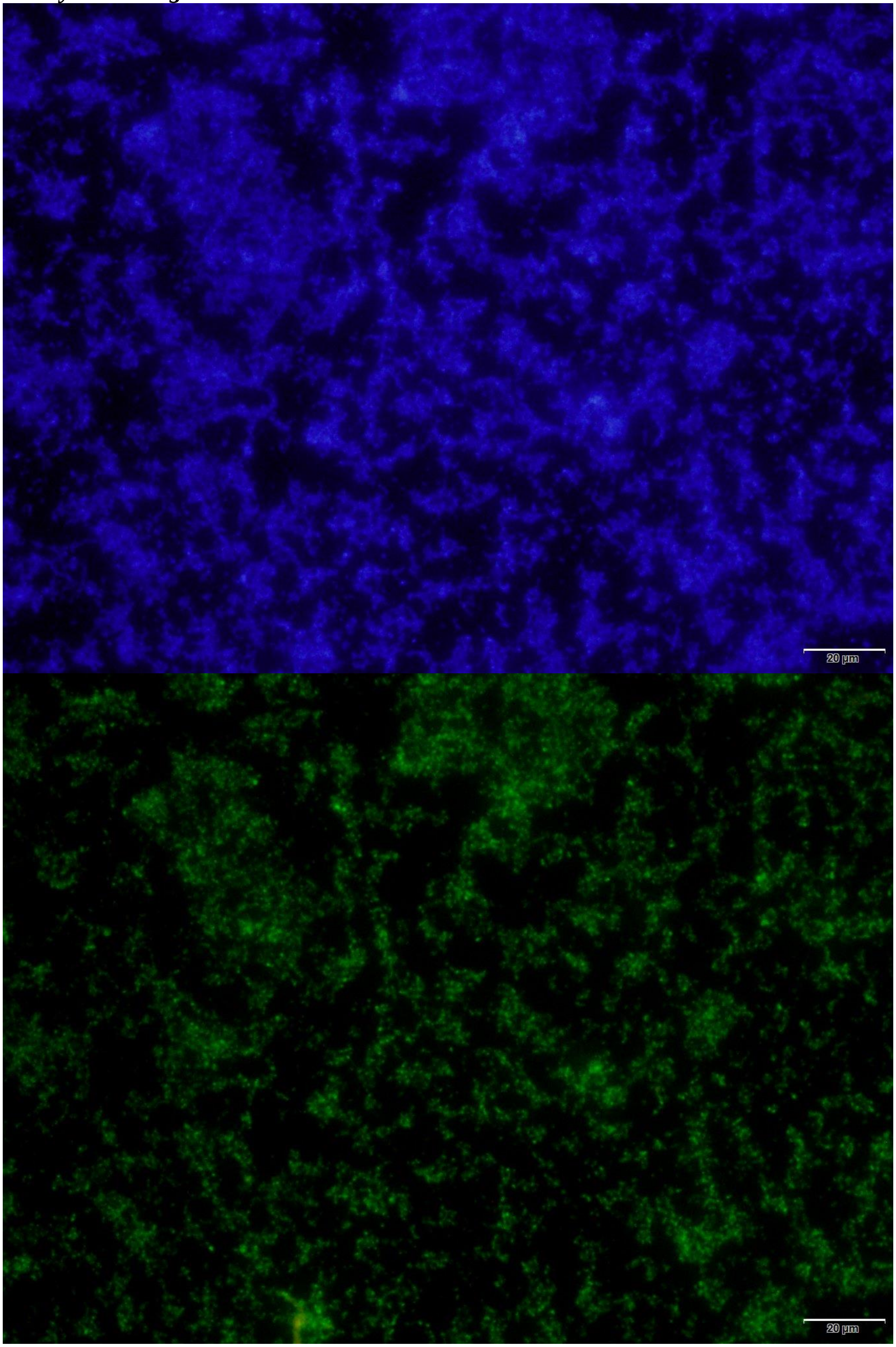

***Fannyhessea vaginae* PB2003/017-T1-2**

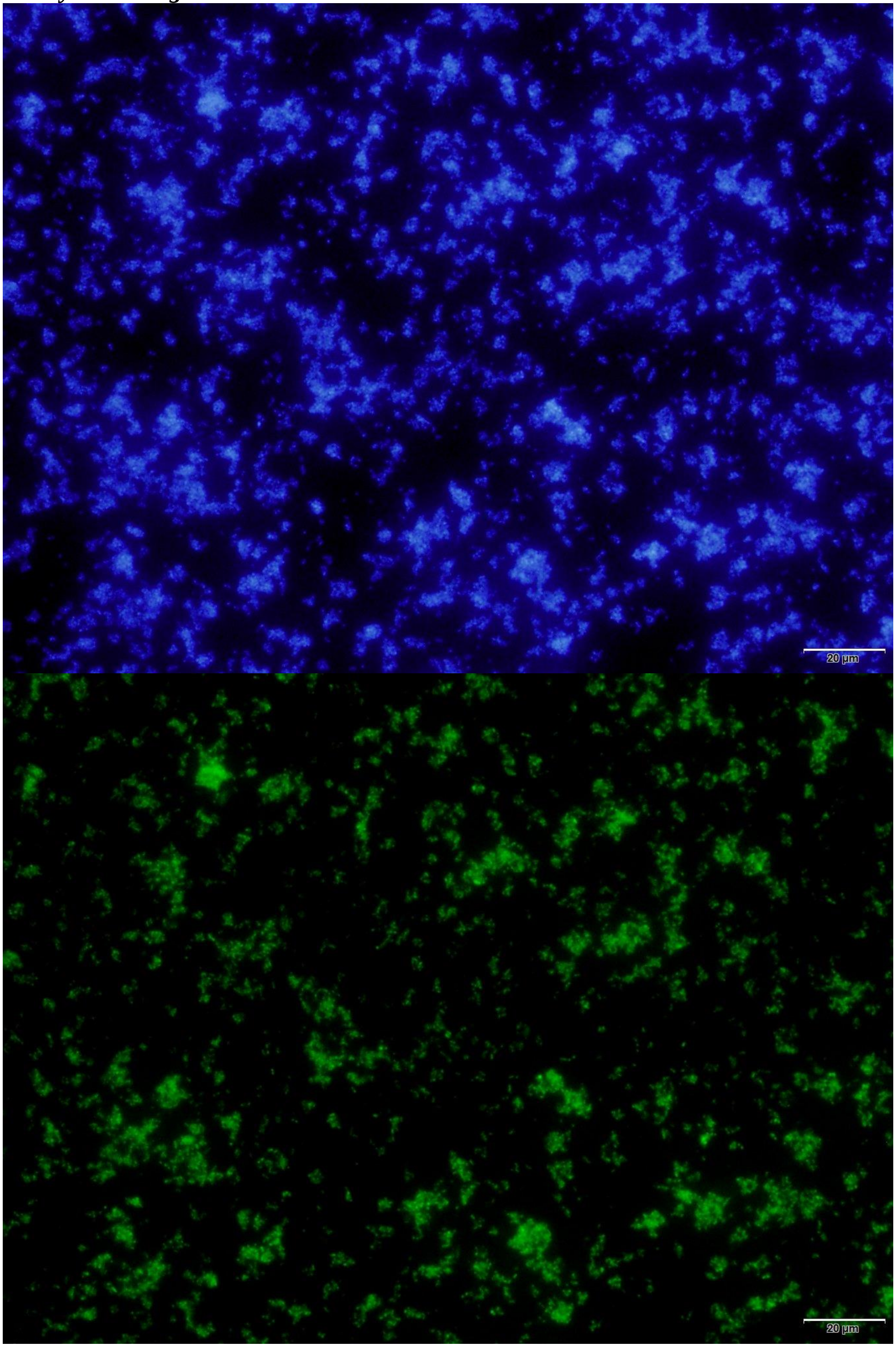

Fannyhessea vaginae **VMF0907COL23**

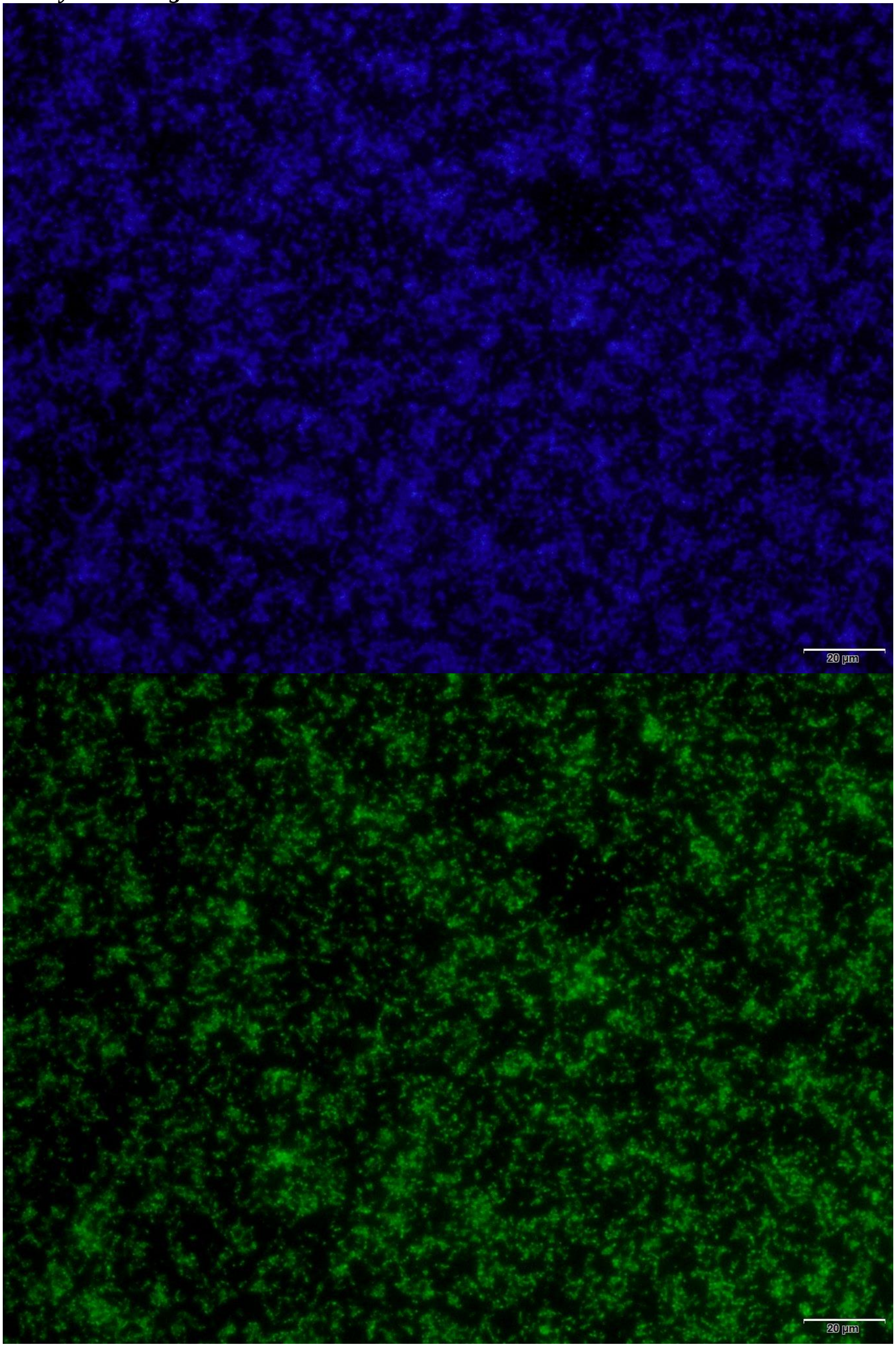

Fannyhessea vaginae **VMF0914COL13**

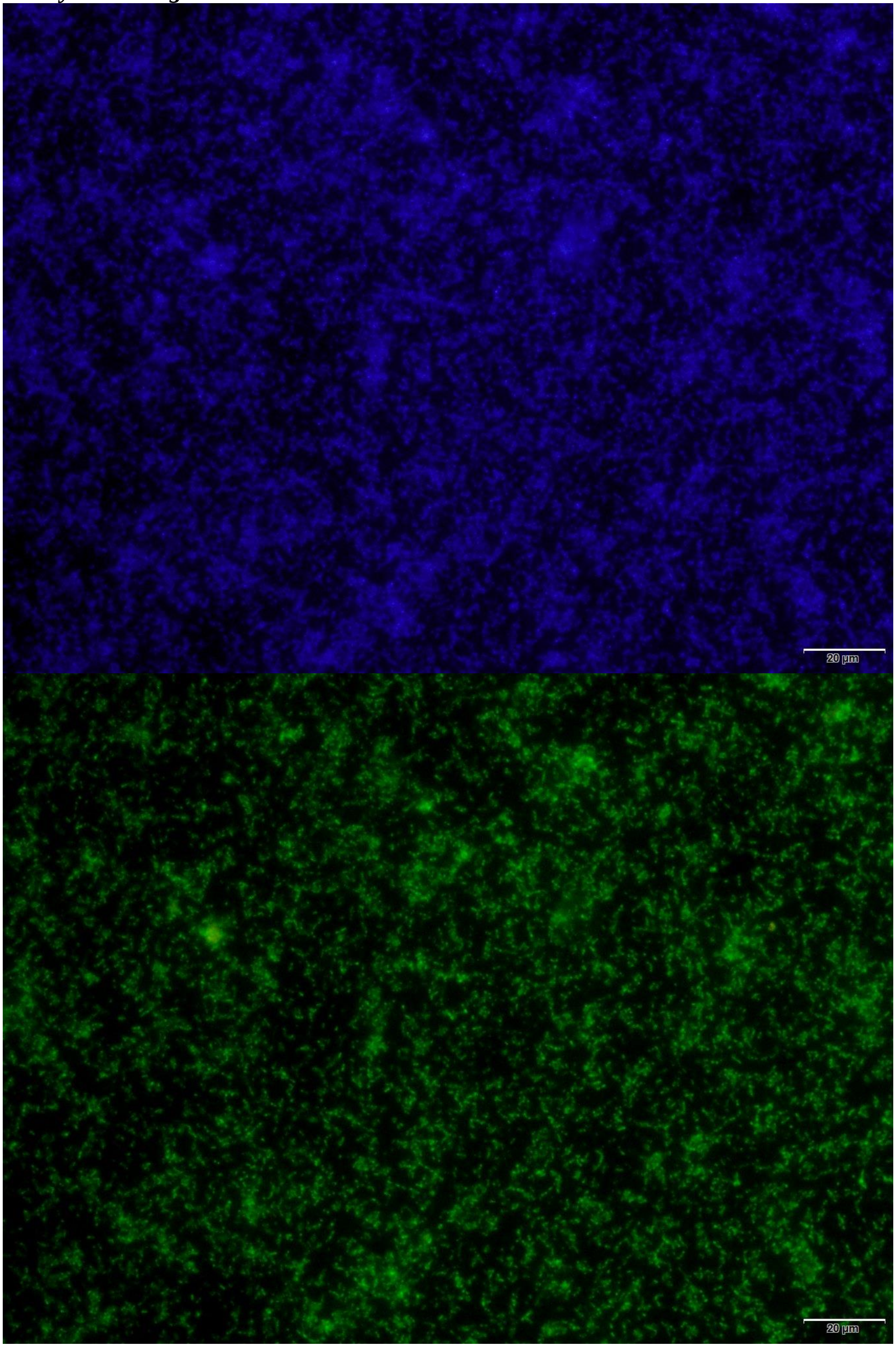

***Fannyhessea vaginae* VMF0914COL43**

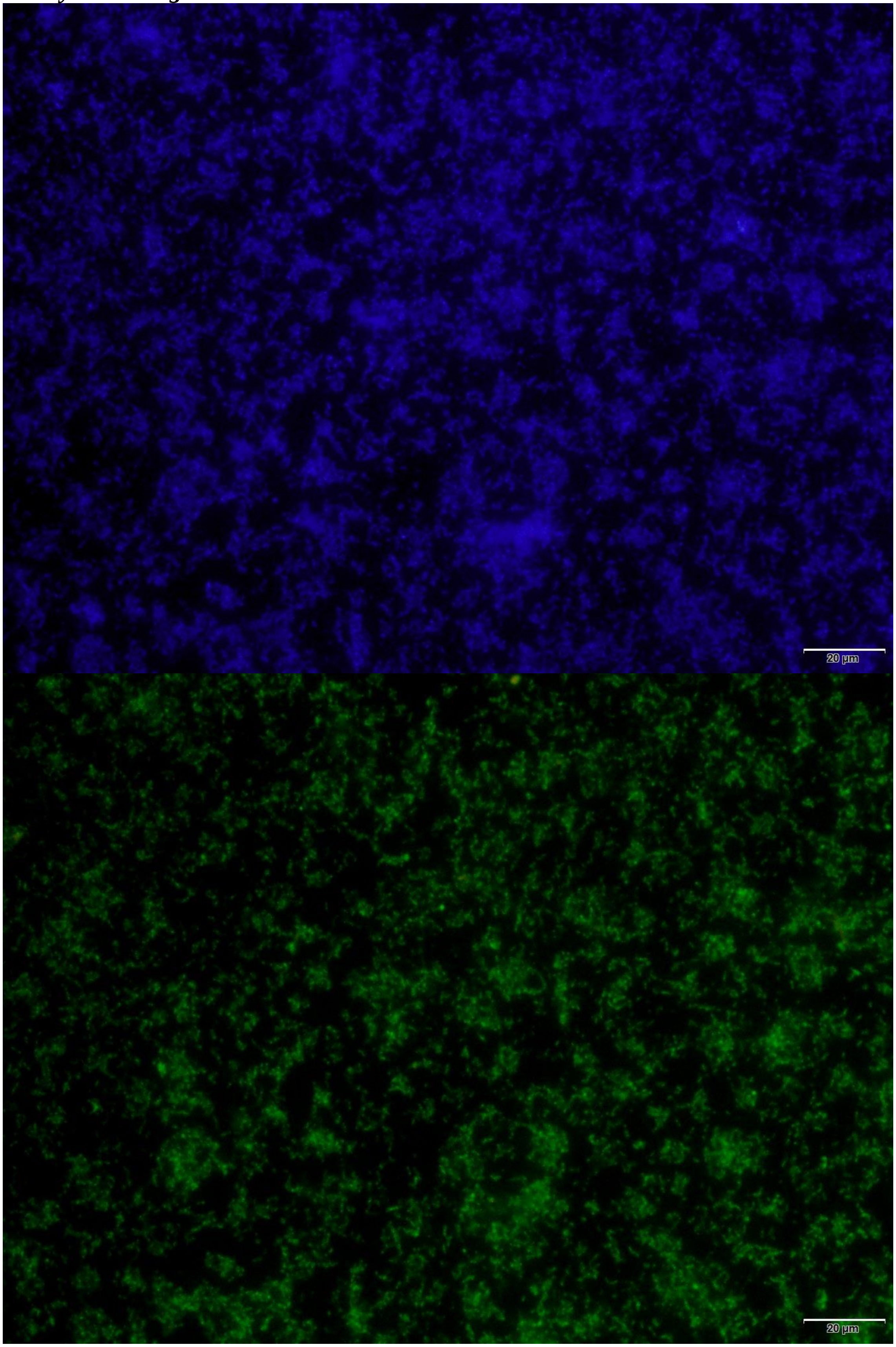

***Acinetobacter baumanii* CCUG 59798**

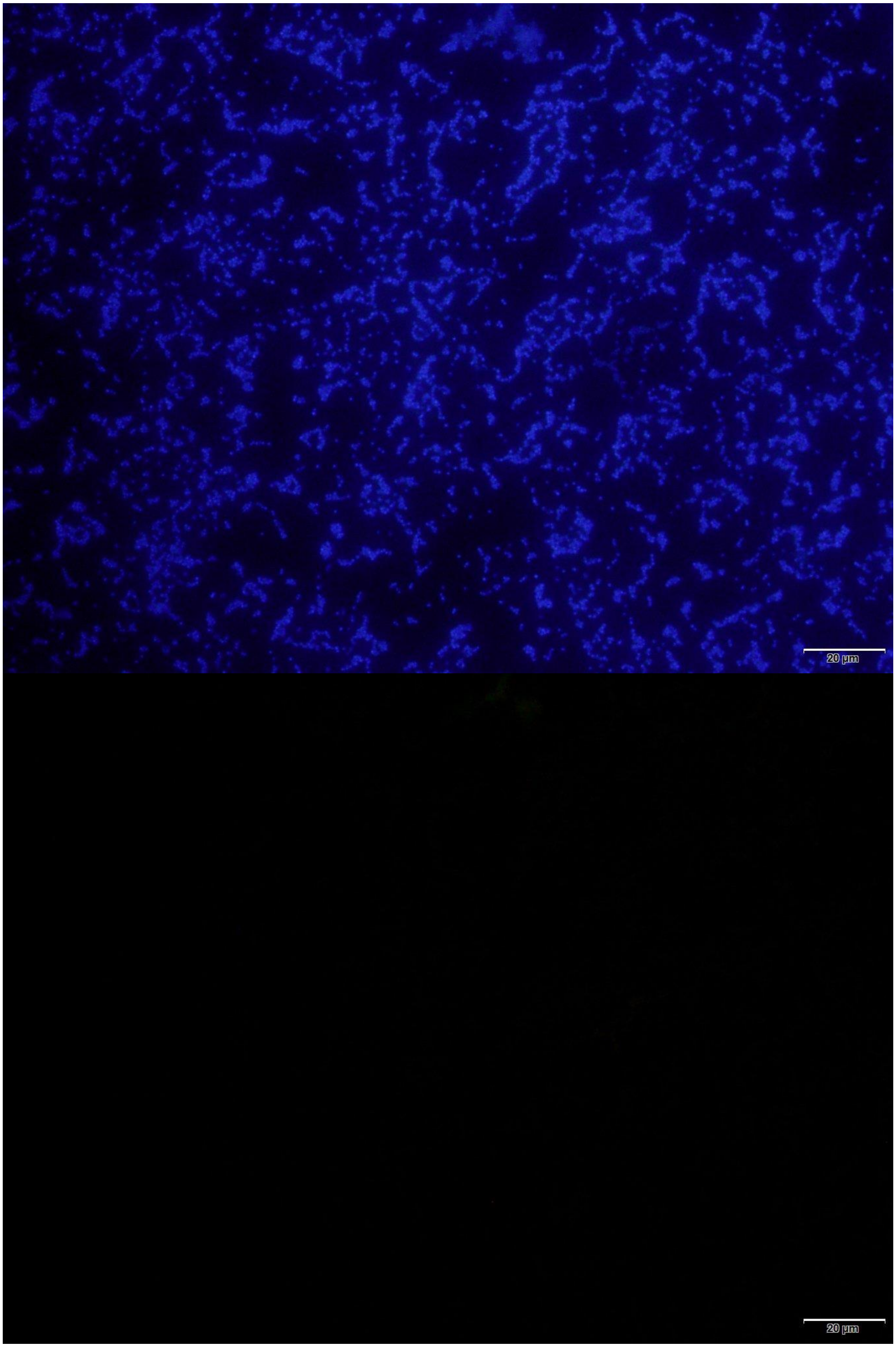

***Actinomyces neuii* UM067**

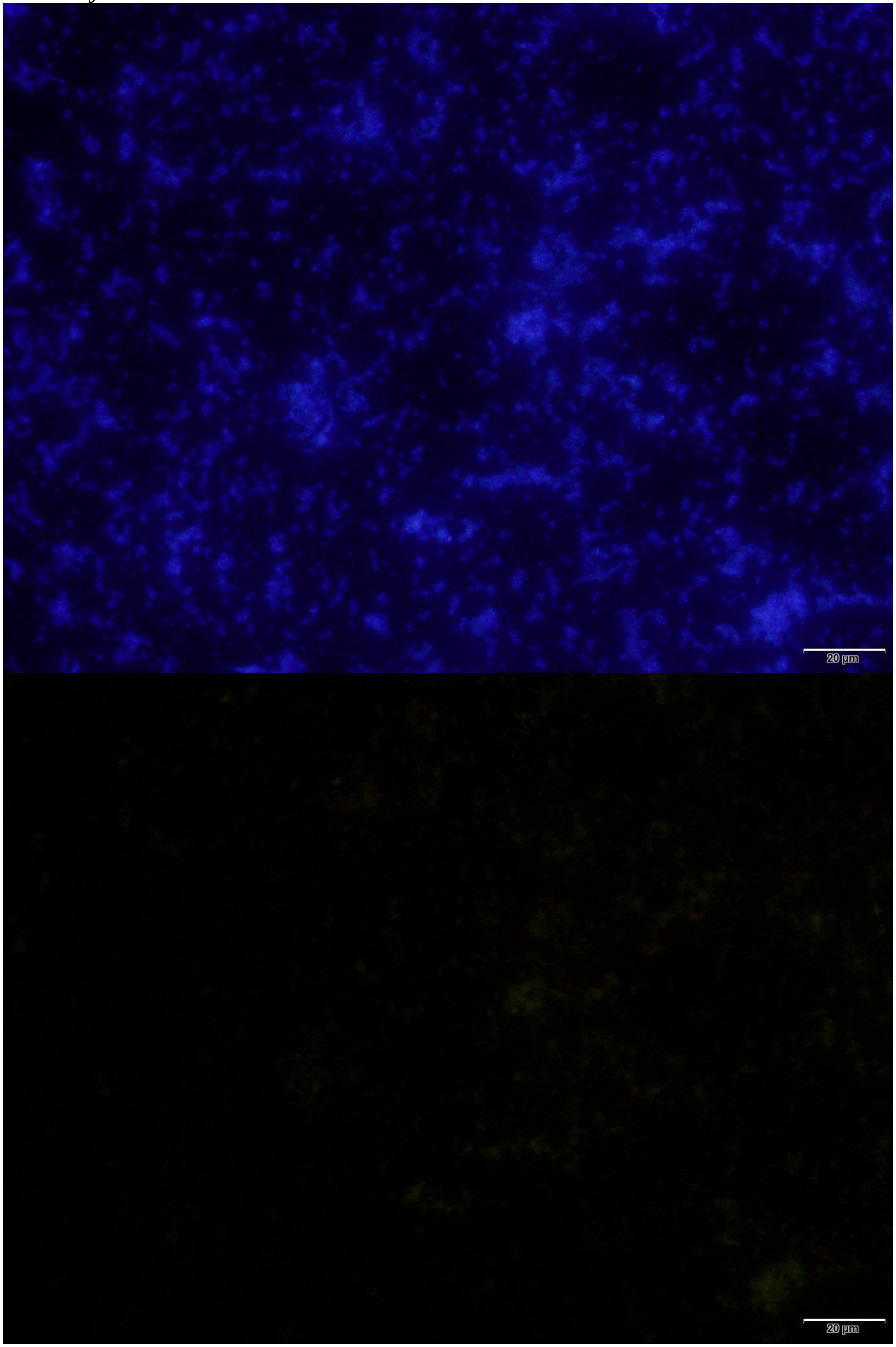

***Actinomyces urogenitalis* CCUG44038**

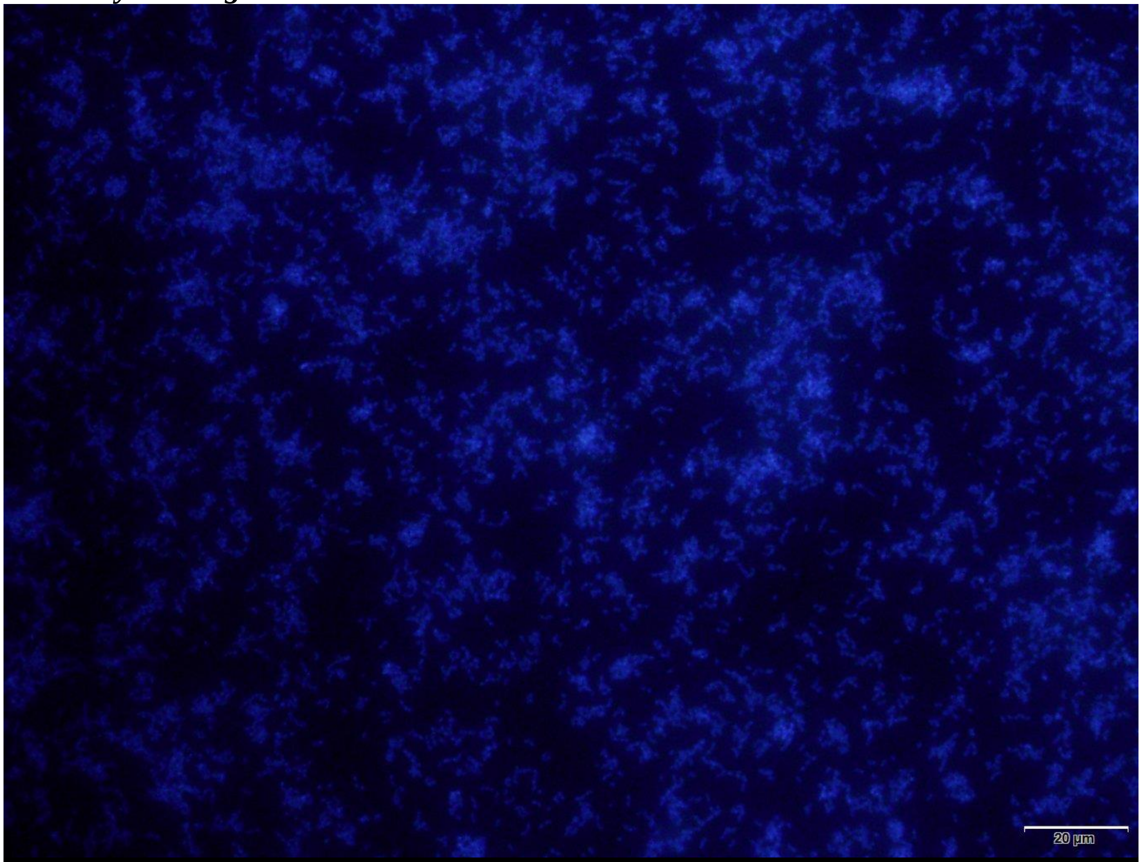

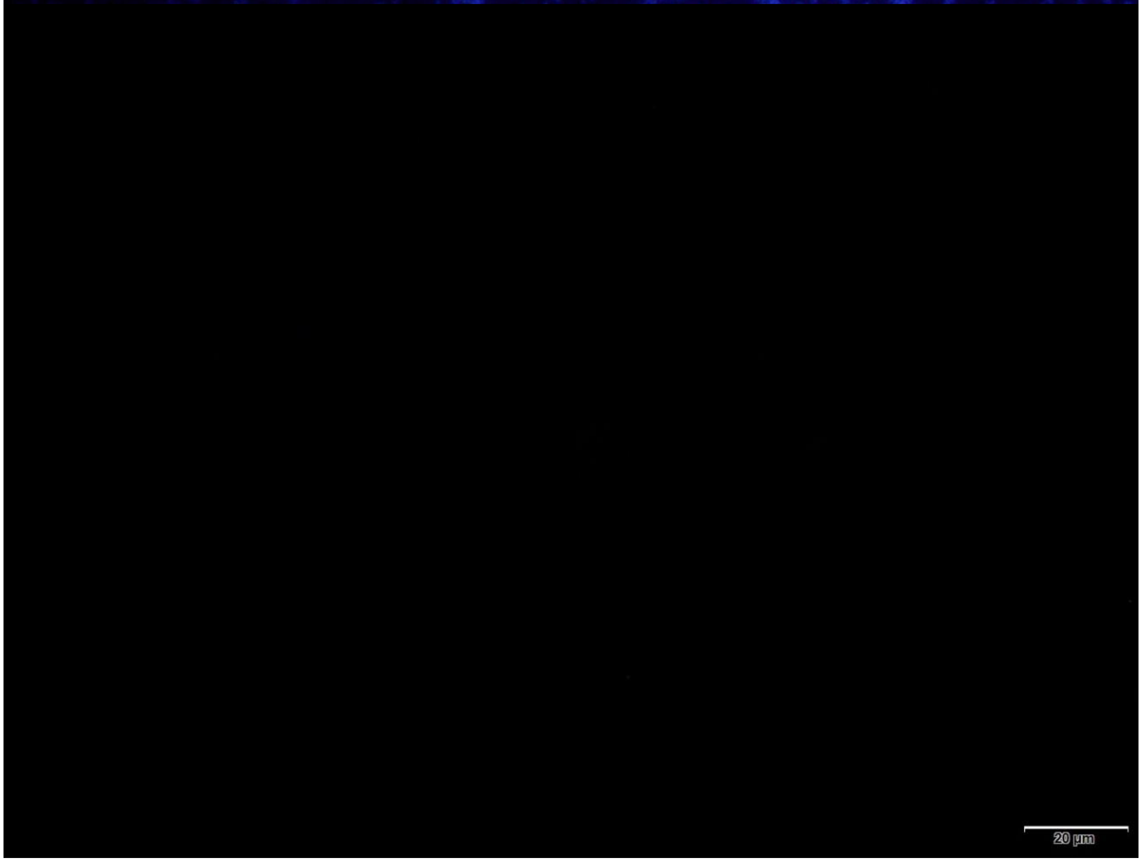

***Aerococcus christensenii* CCUG28826**

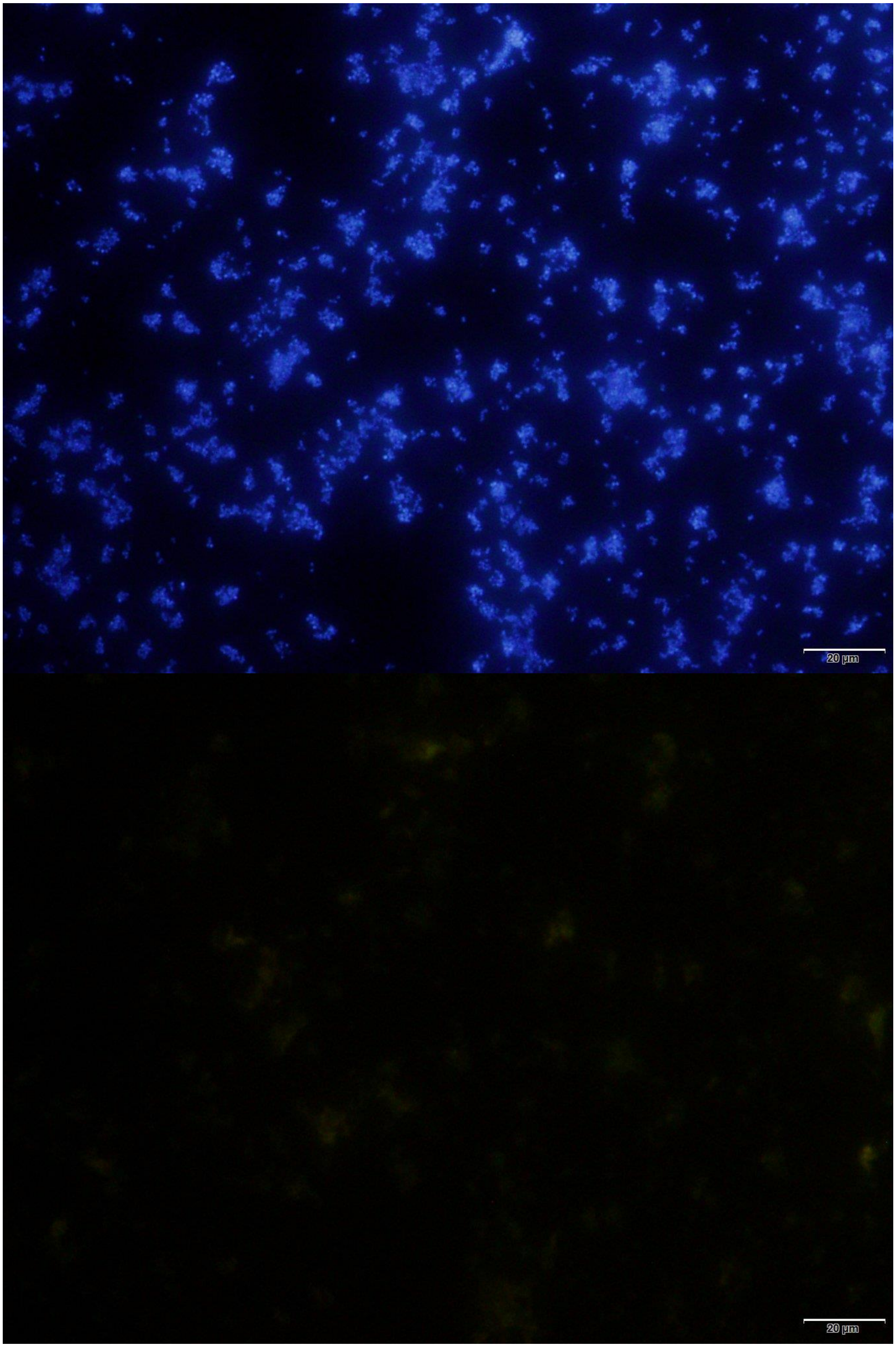

***Bacillus firmus* UM034**

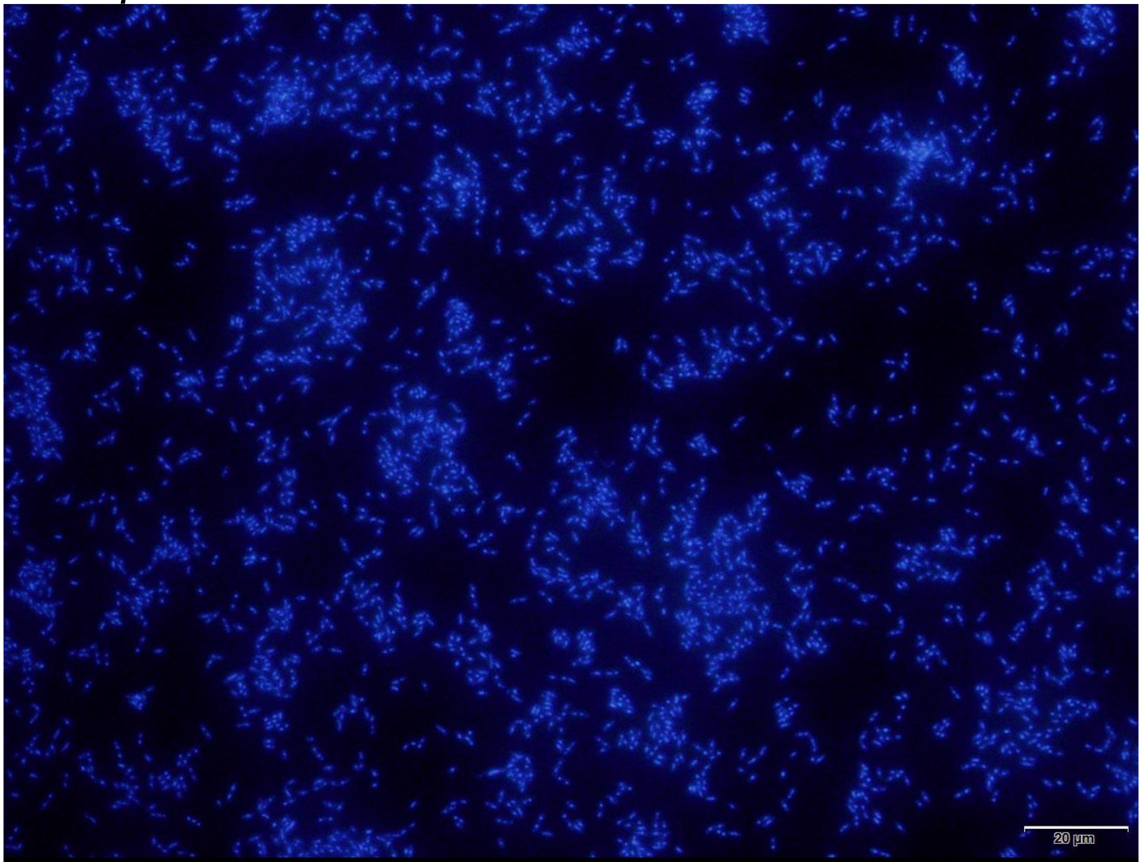

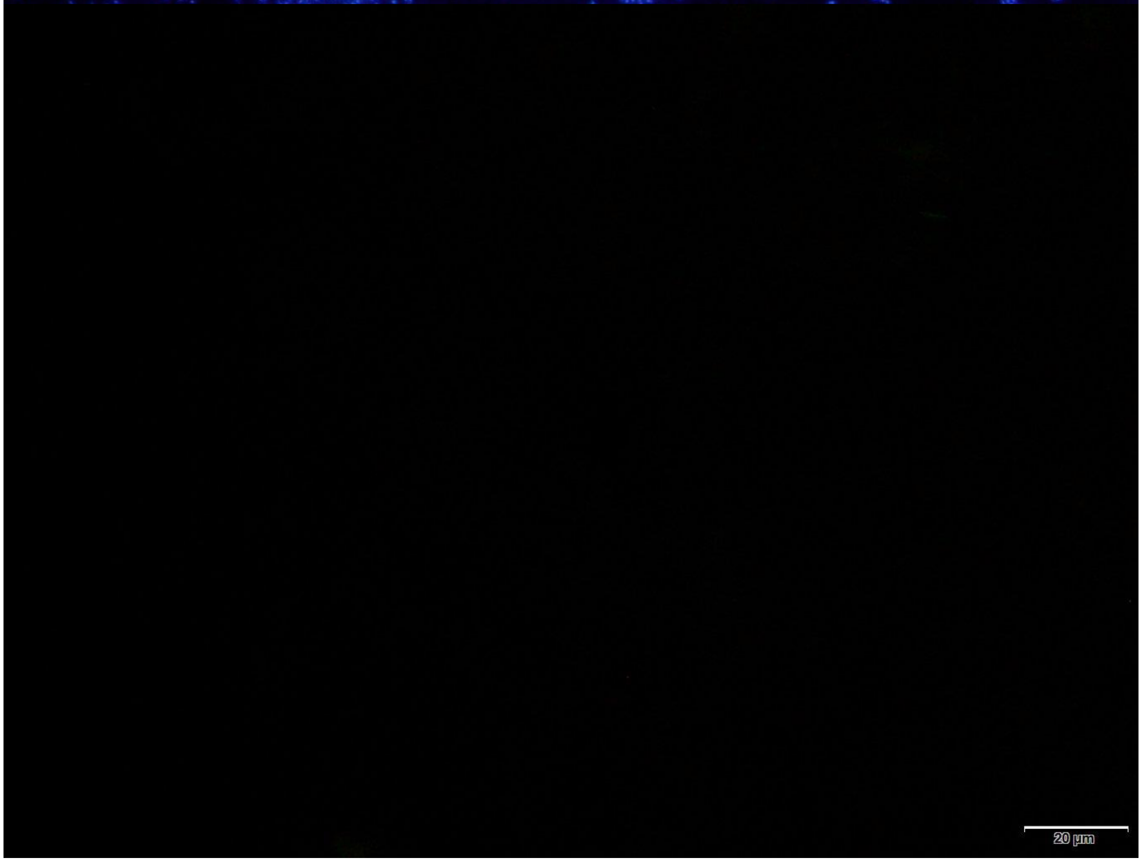

***Bifidobacterium bifidum* CCUG59492**

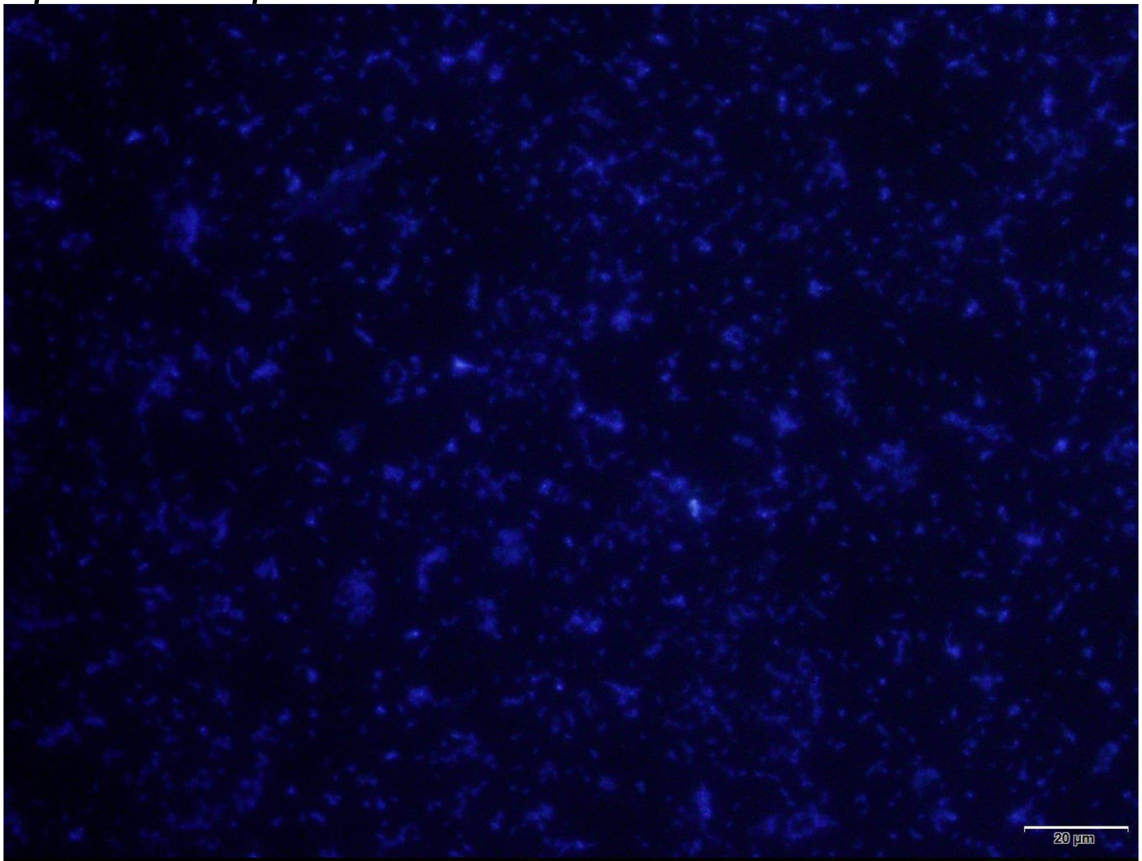

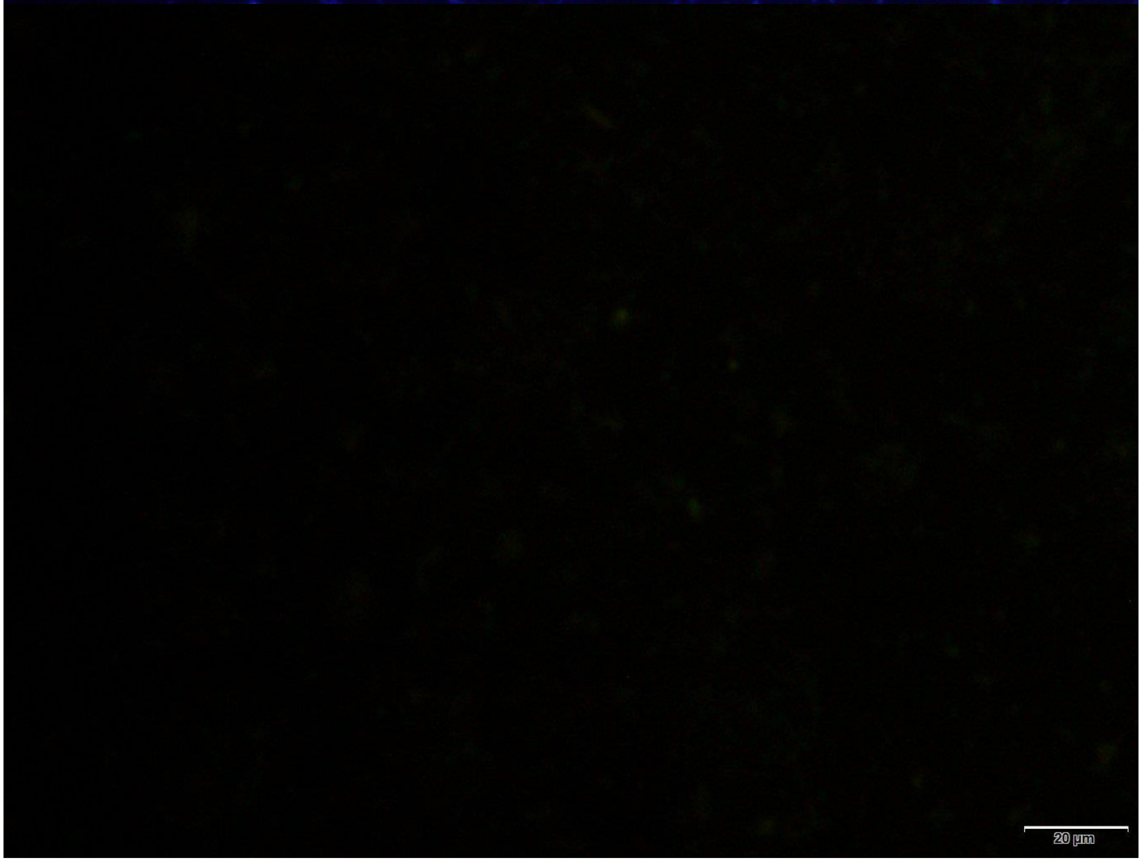

***Brevibacterium ravenspurgense* CCUG42923**

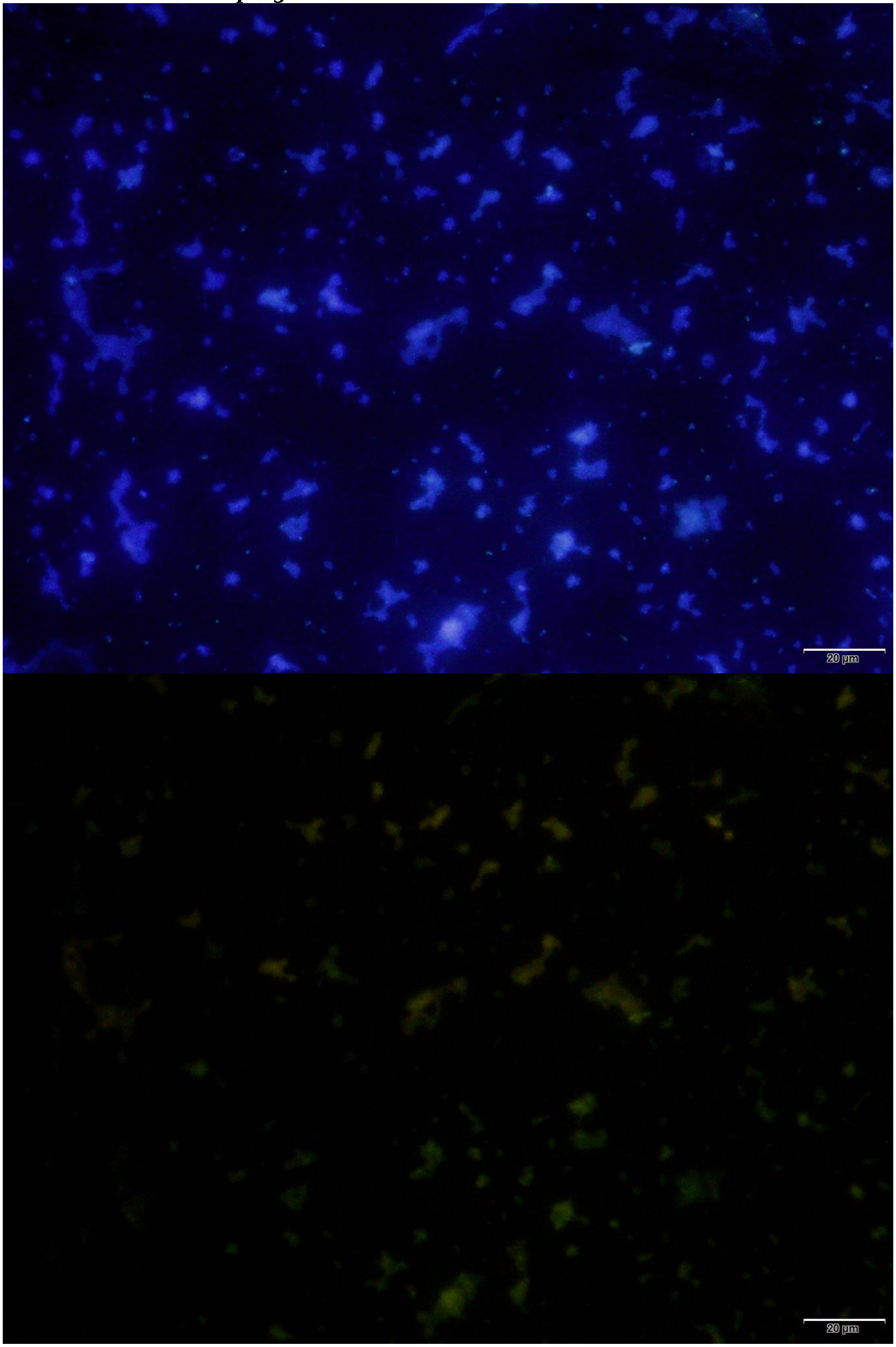

***Campylobacter ureolyticus* CCUG 44295**

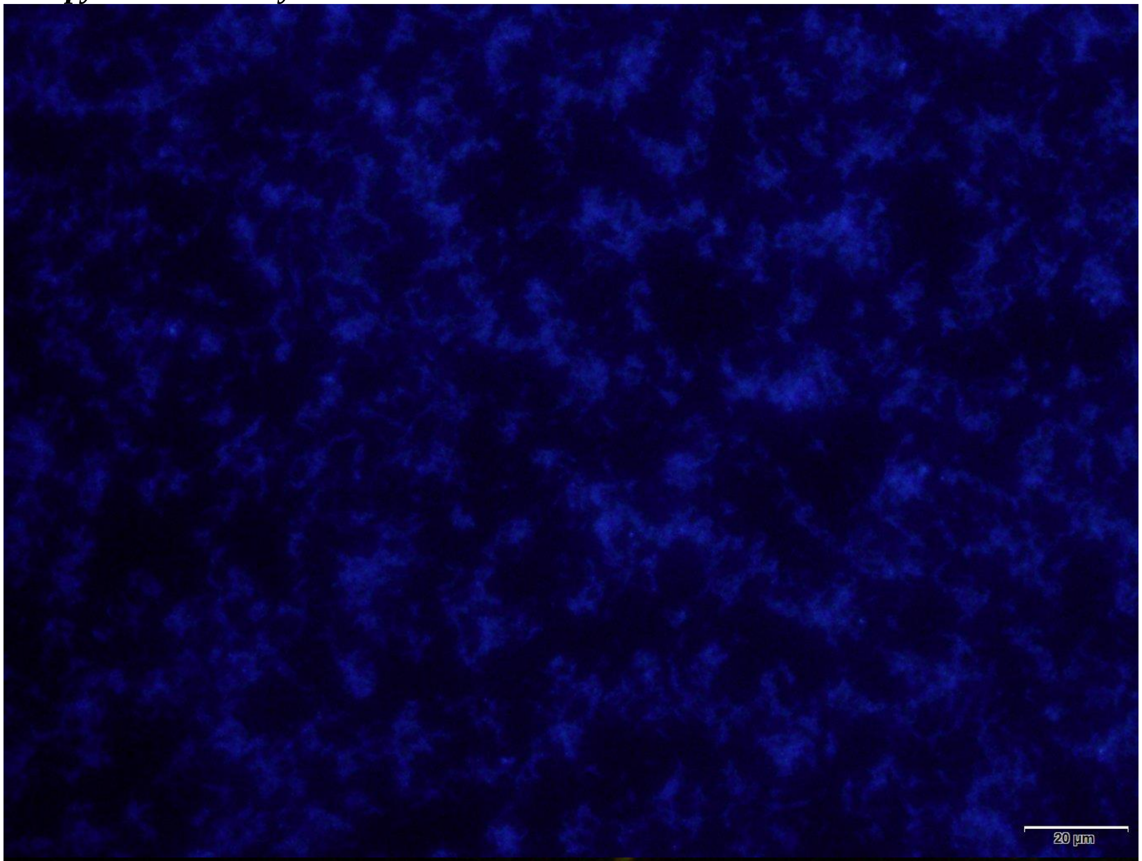

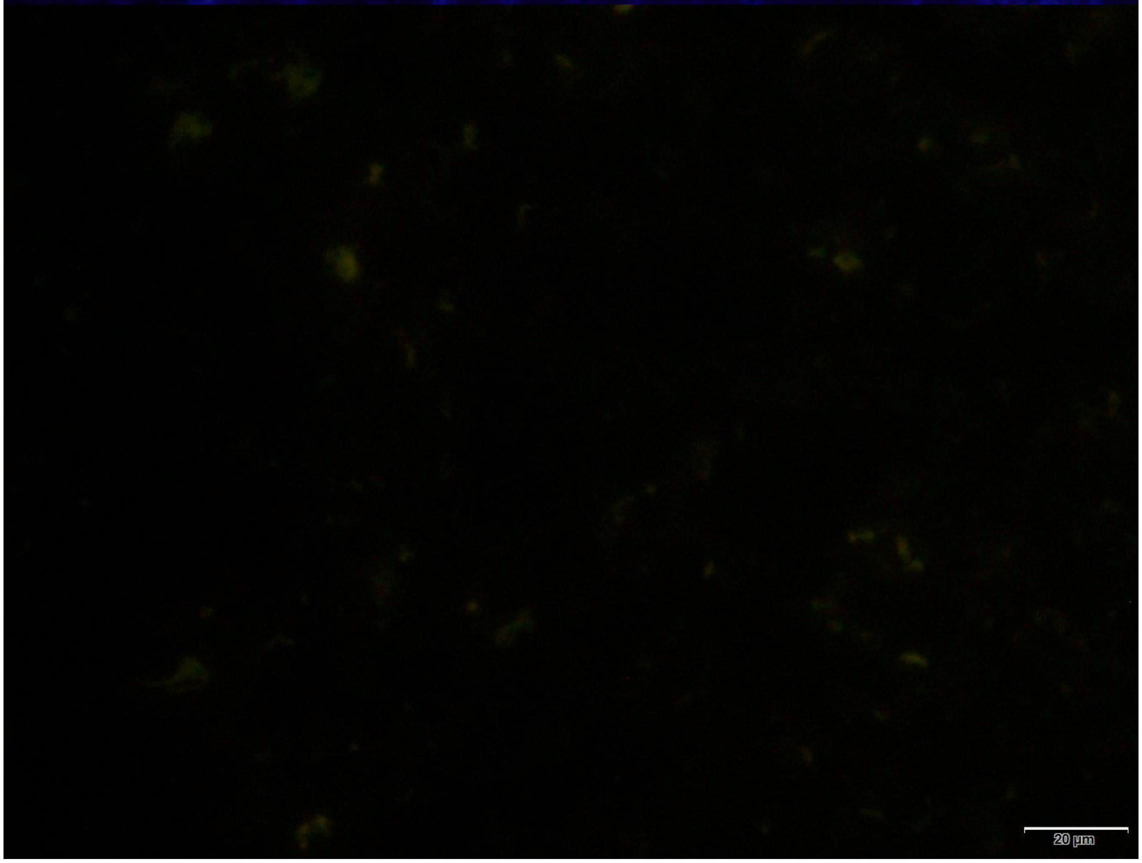

***Corynebacterium tuscaniense* UM137**

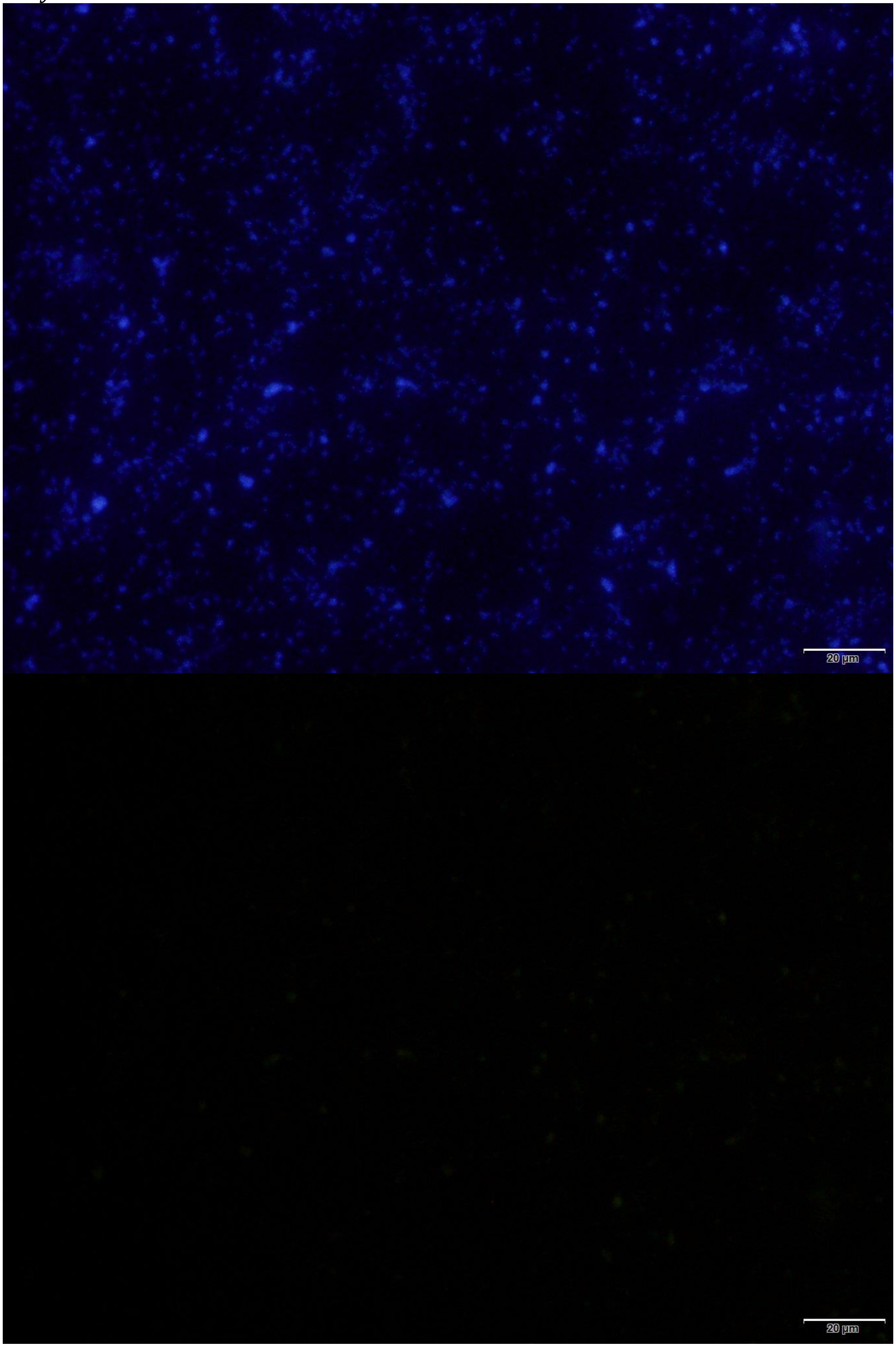

***Enterococcus faecalis* UM035**

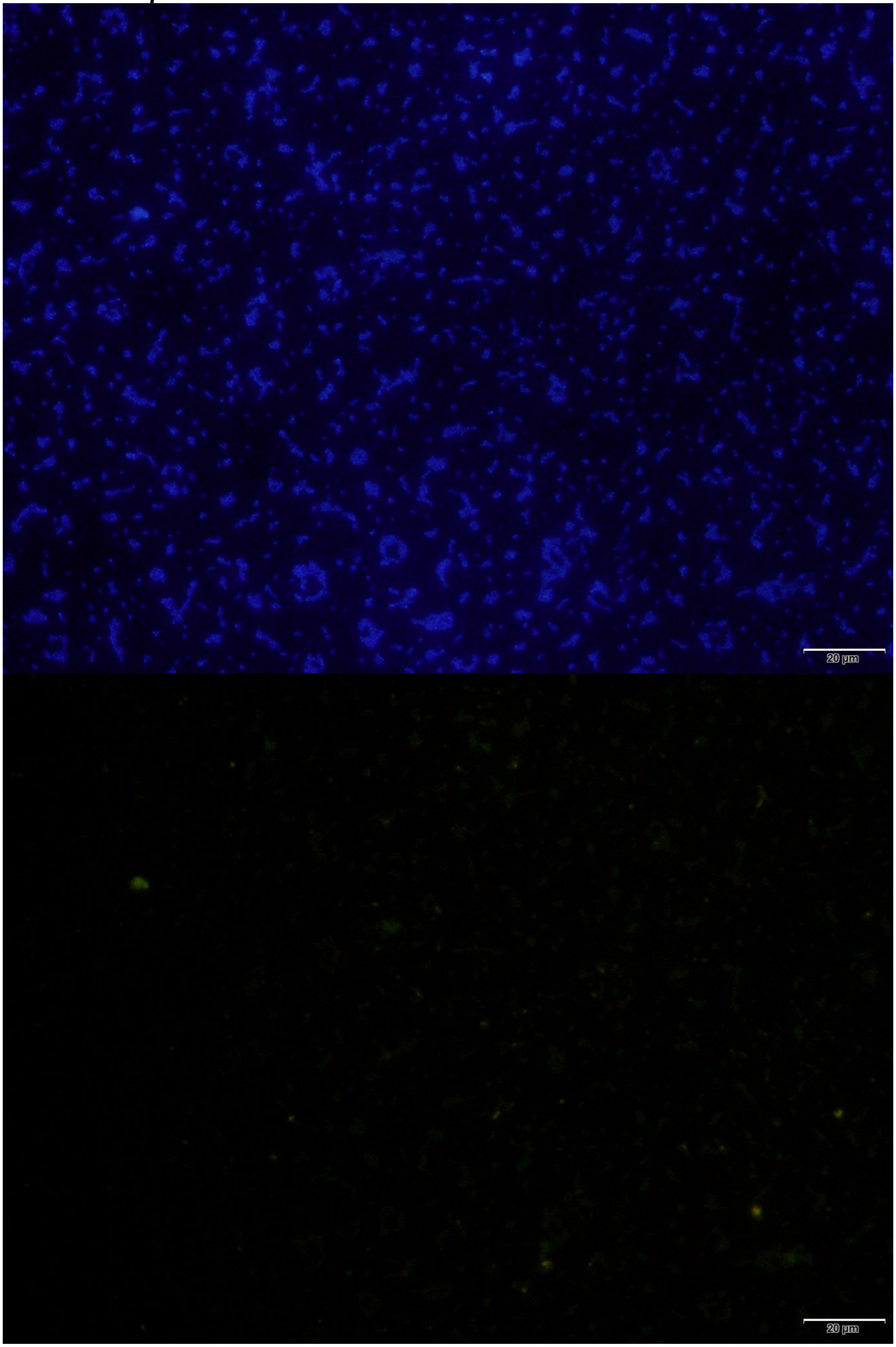

***Escherichia coli* UM056**

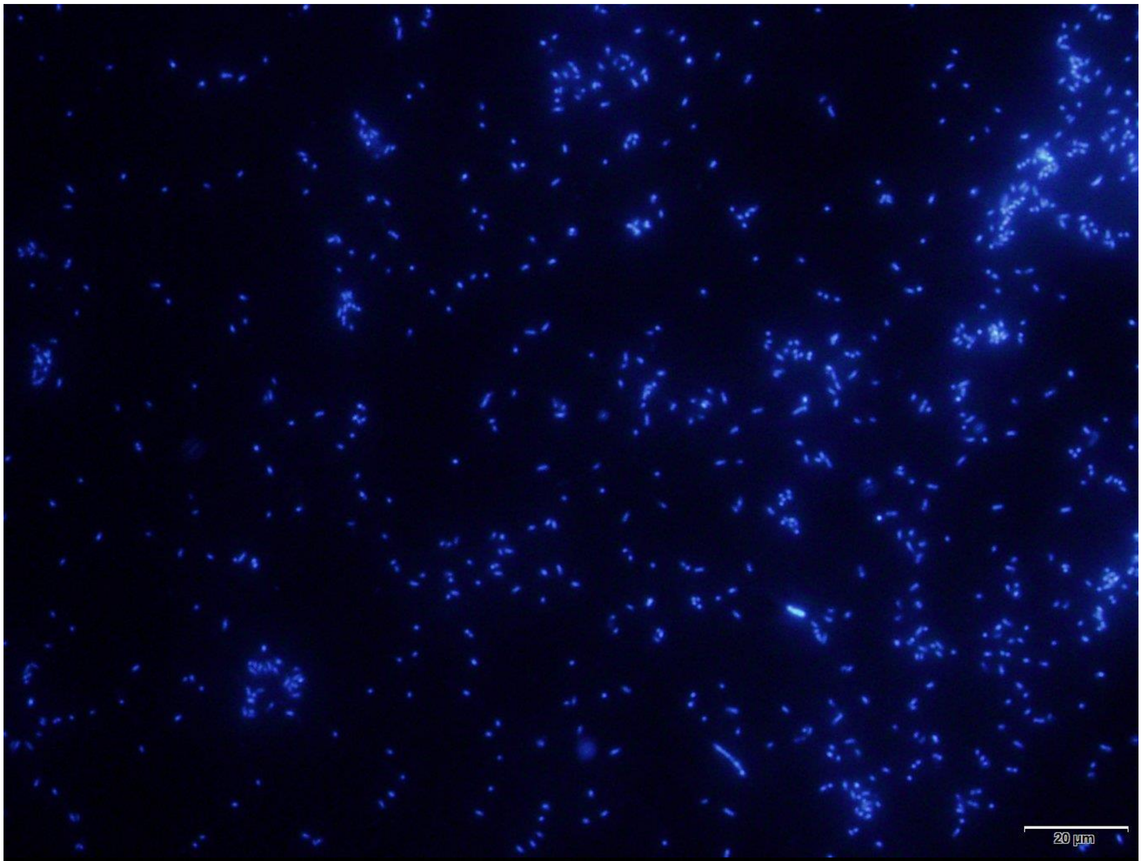

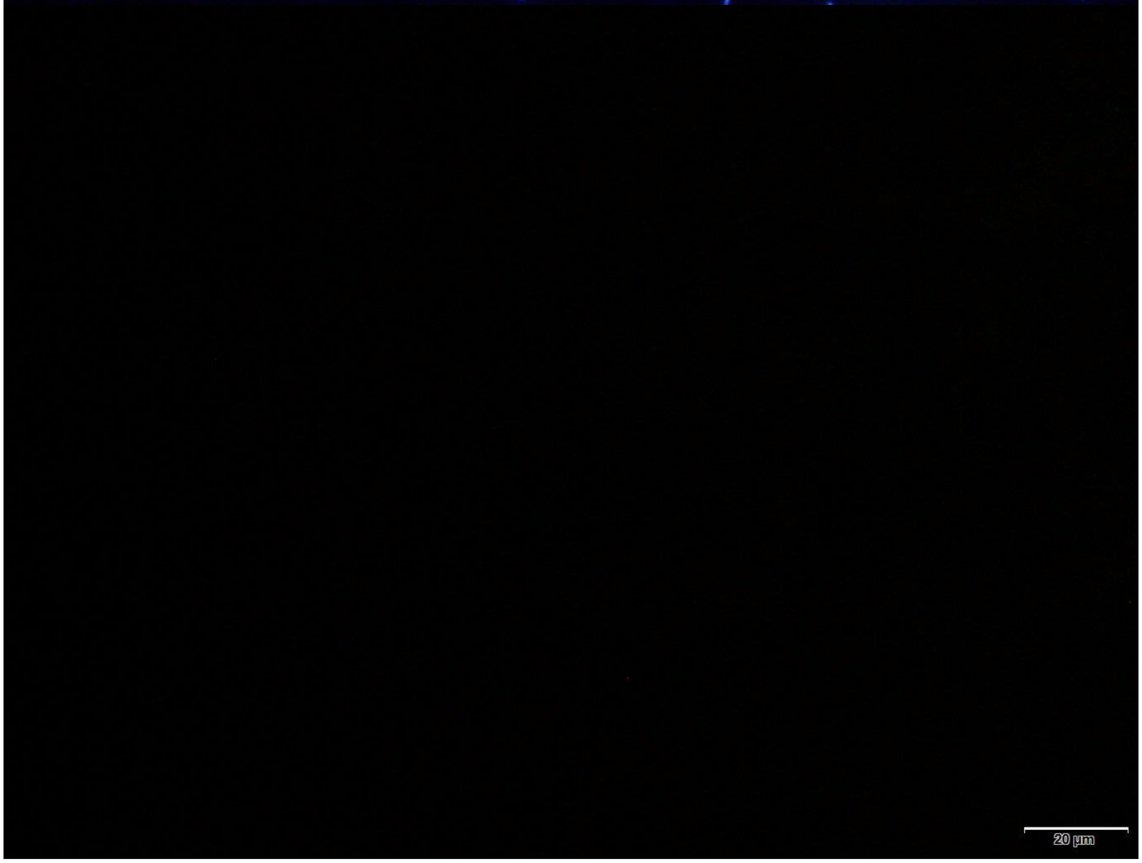

***Gardnerella leopoldii* UM034**

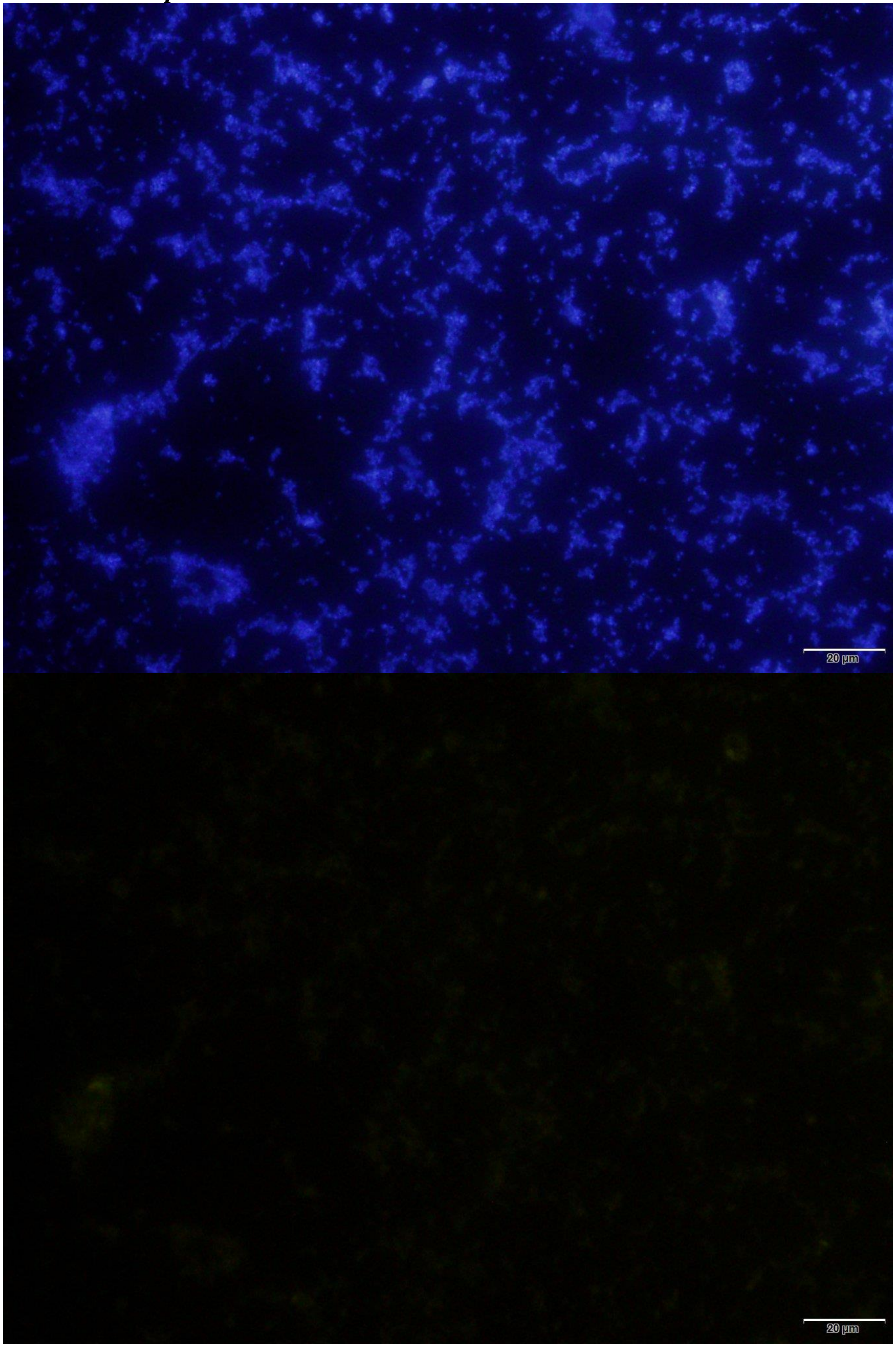

***Gardnerella piotti* UM035**

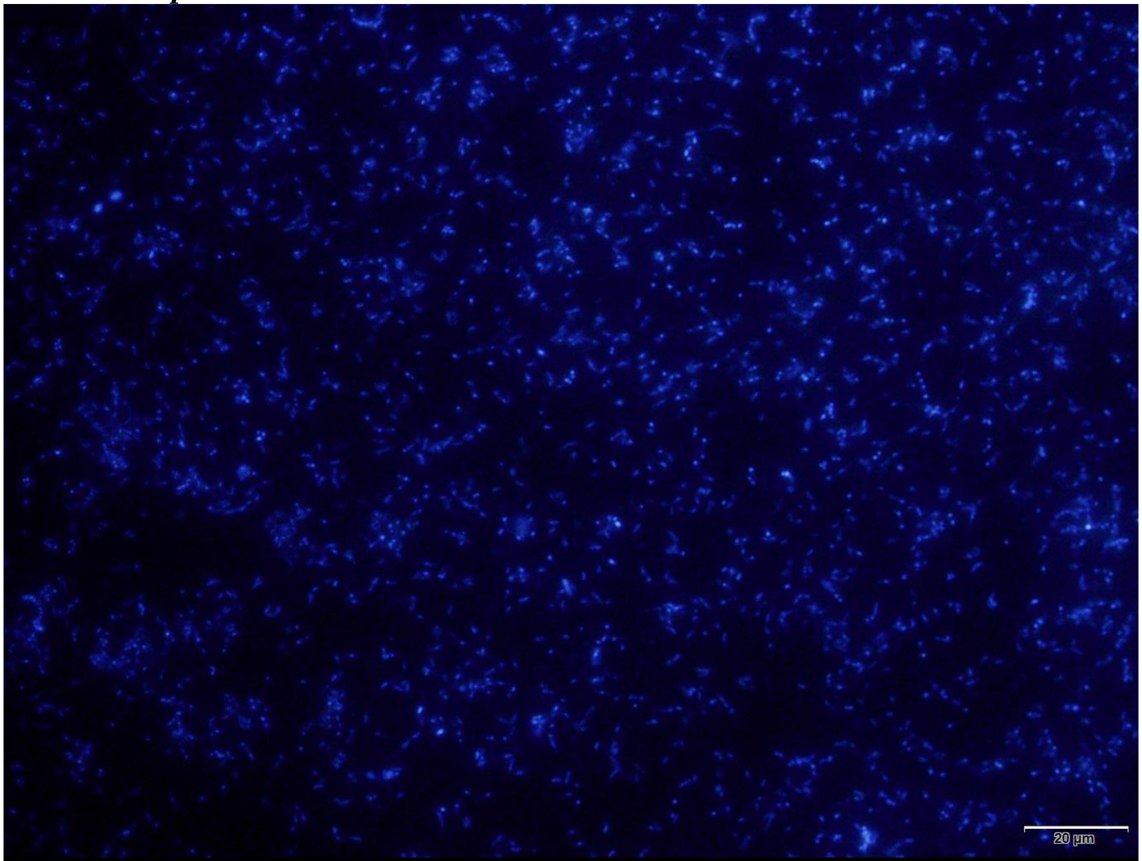

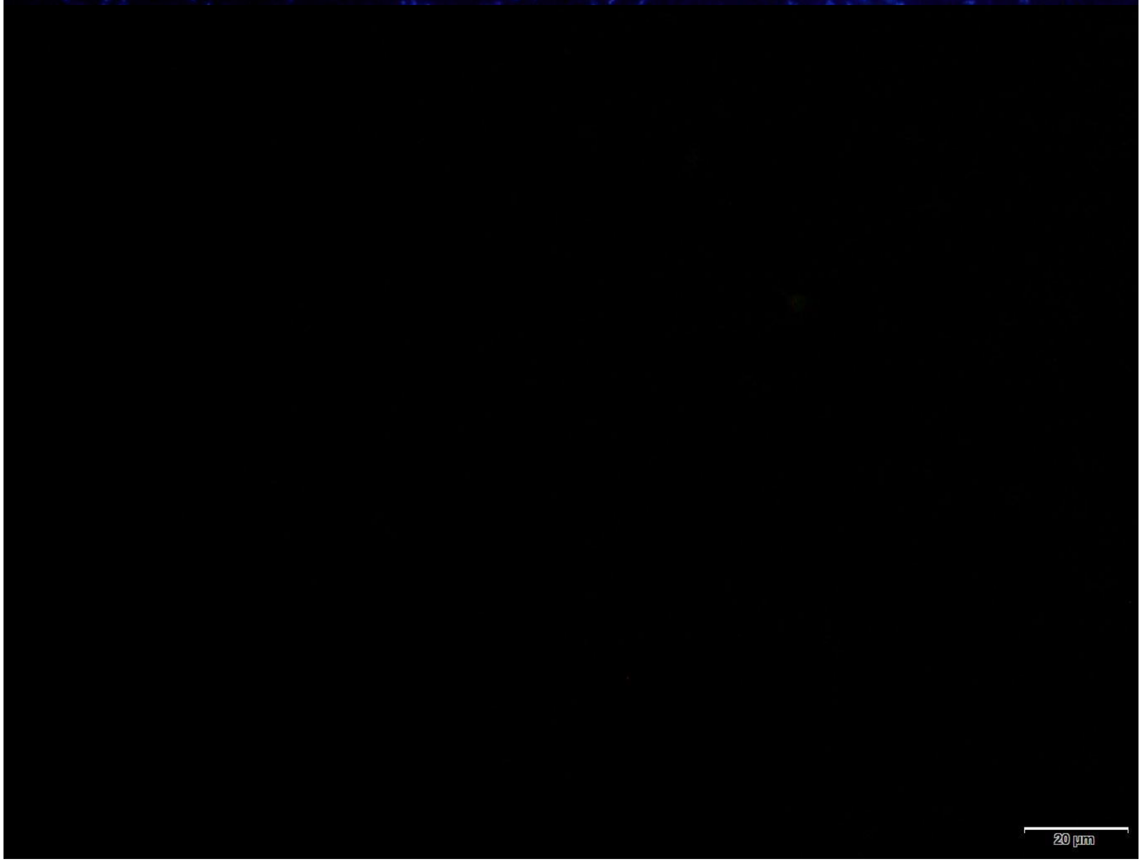

***Gardnerella swidsinskii* UM094**

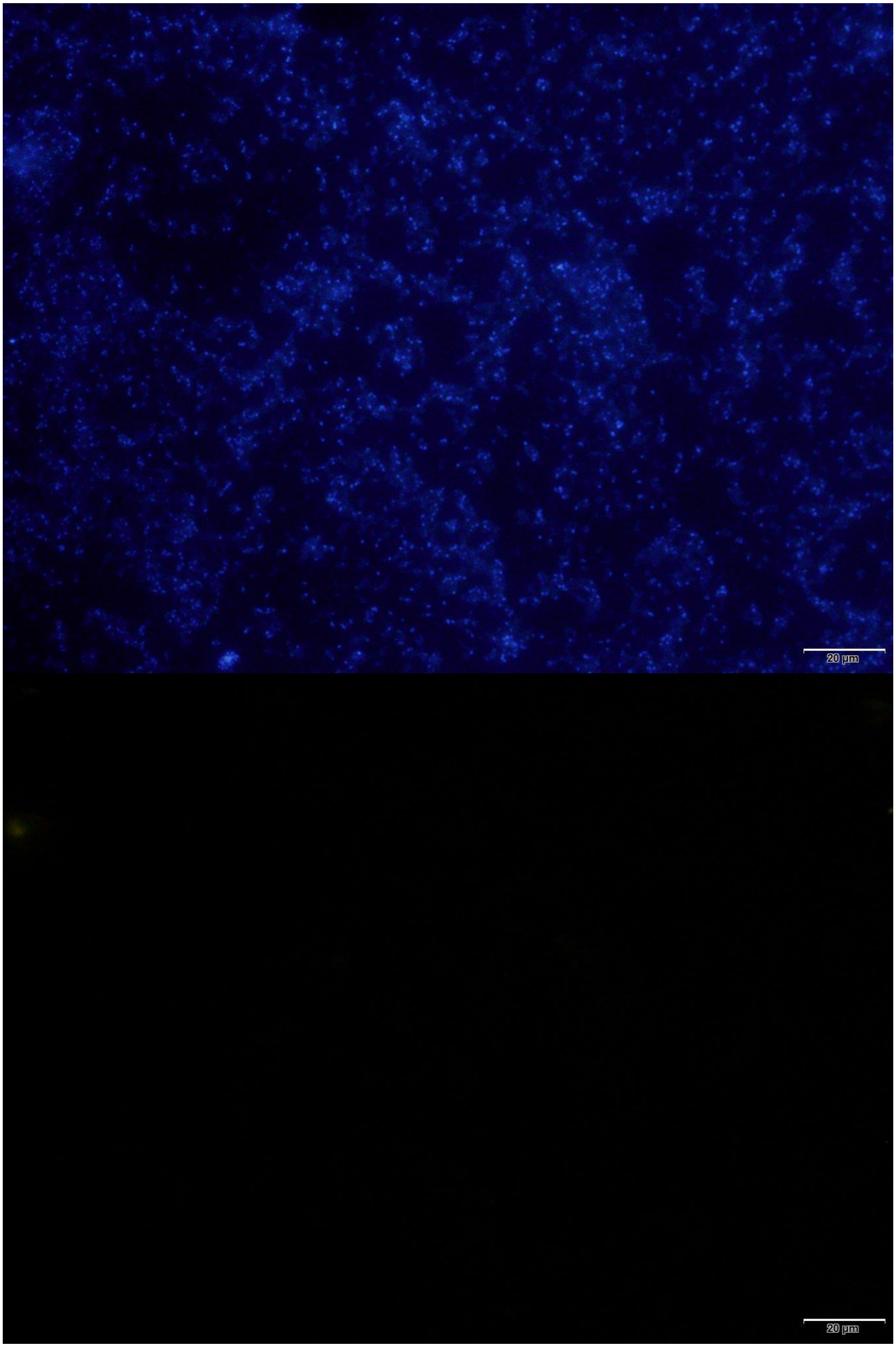

***Gemella haemolysans* UM034**

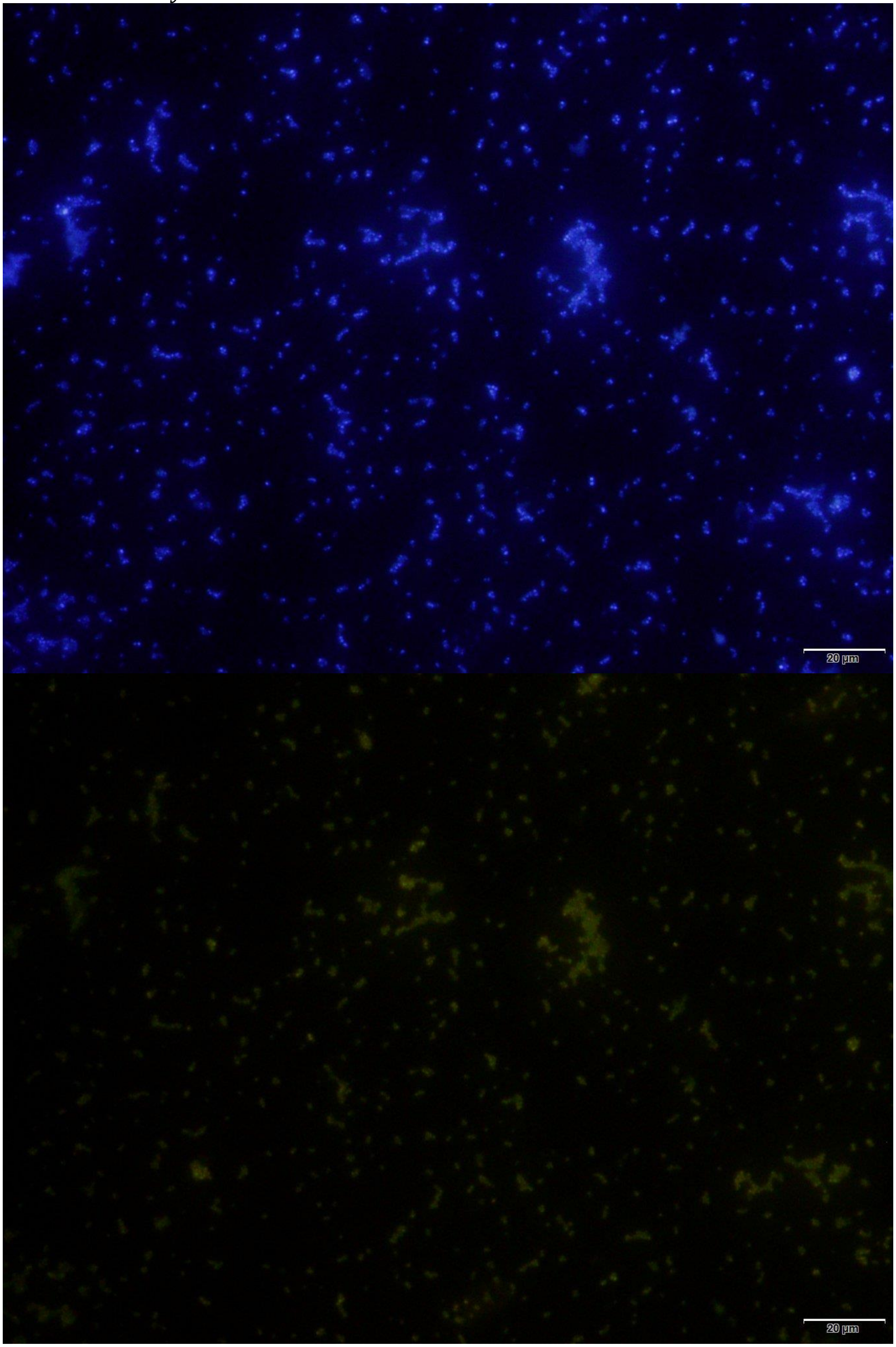

***Lactobacillus gasseri* ATCC9857**

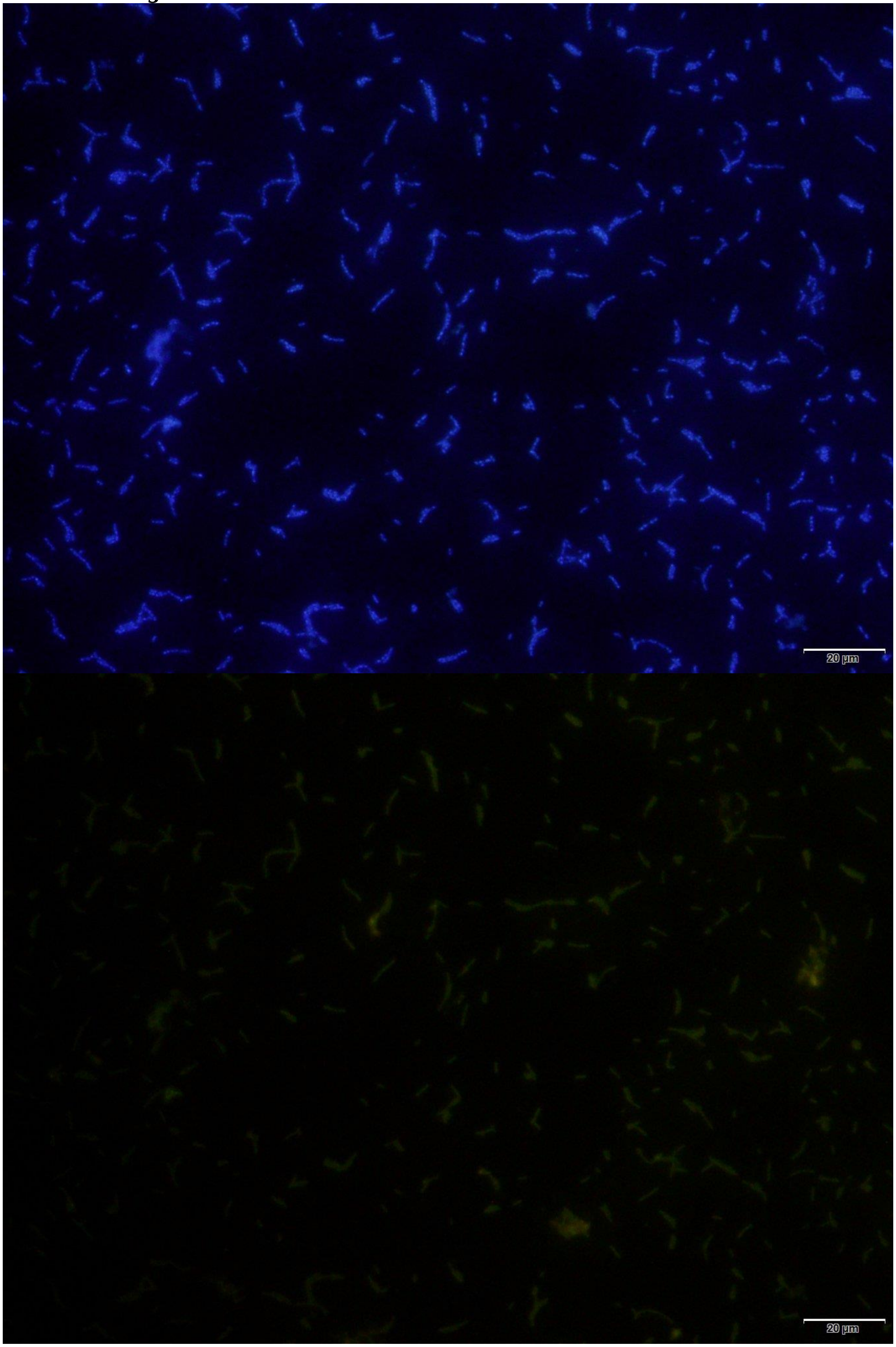

***Lactobacillus rhamnosus* CECT 288**

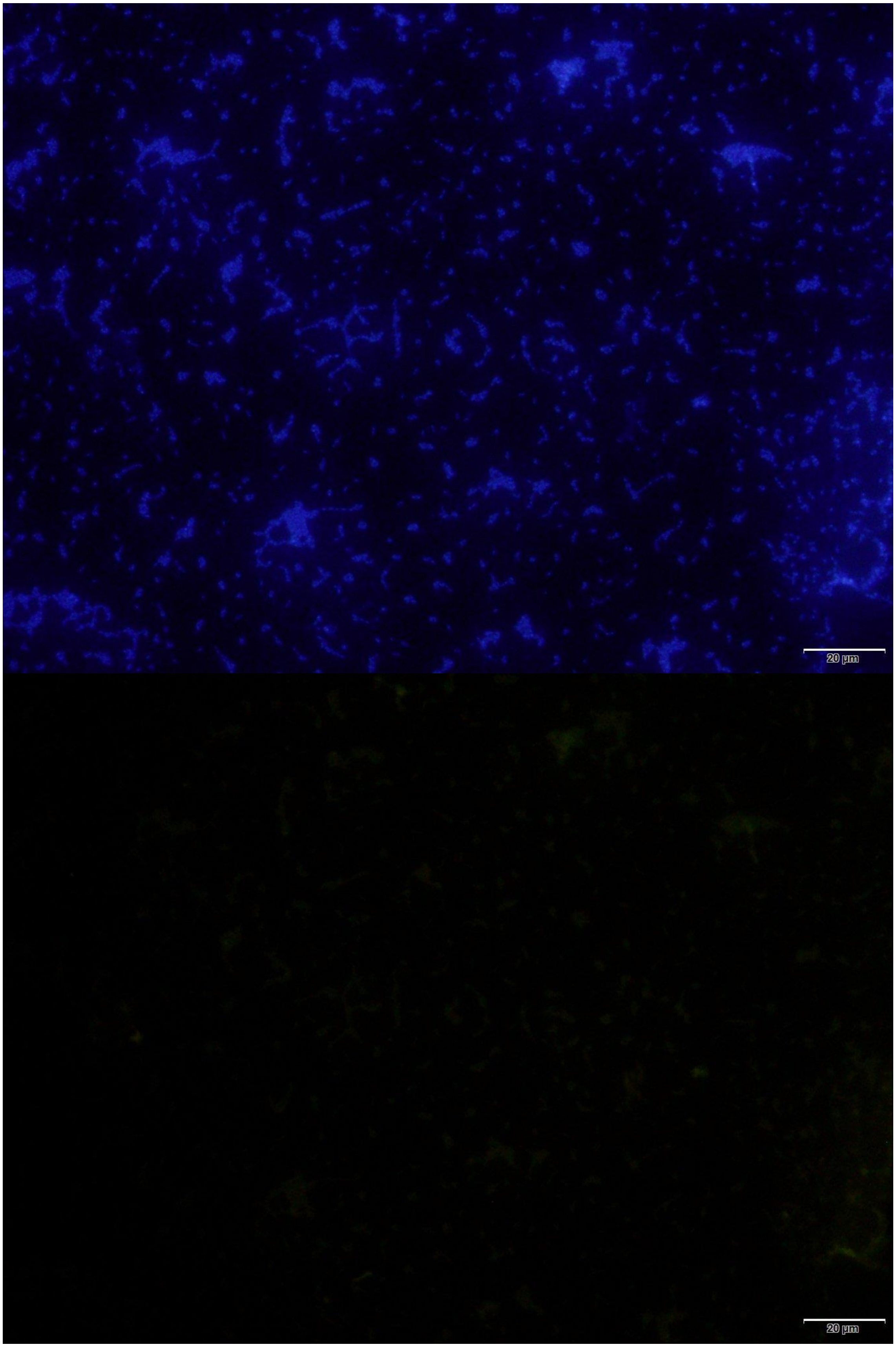

***Lactobacillus vaginalis* UM062**

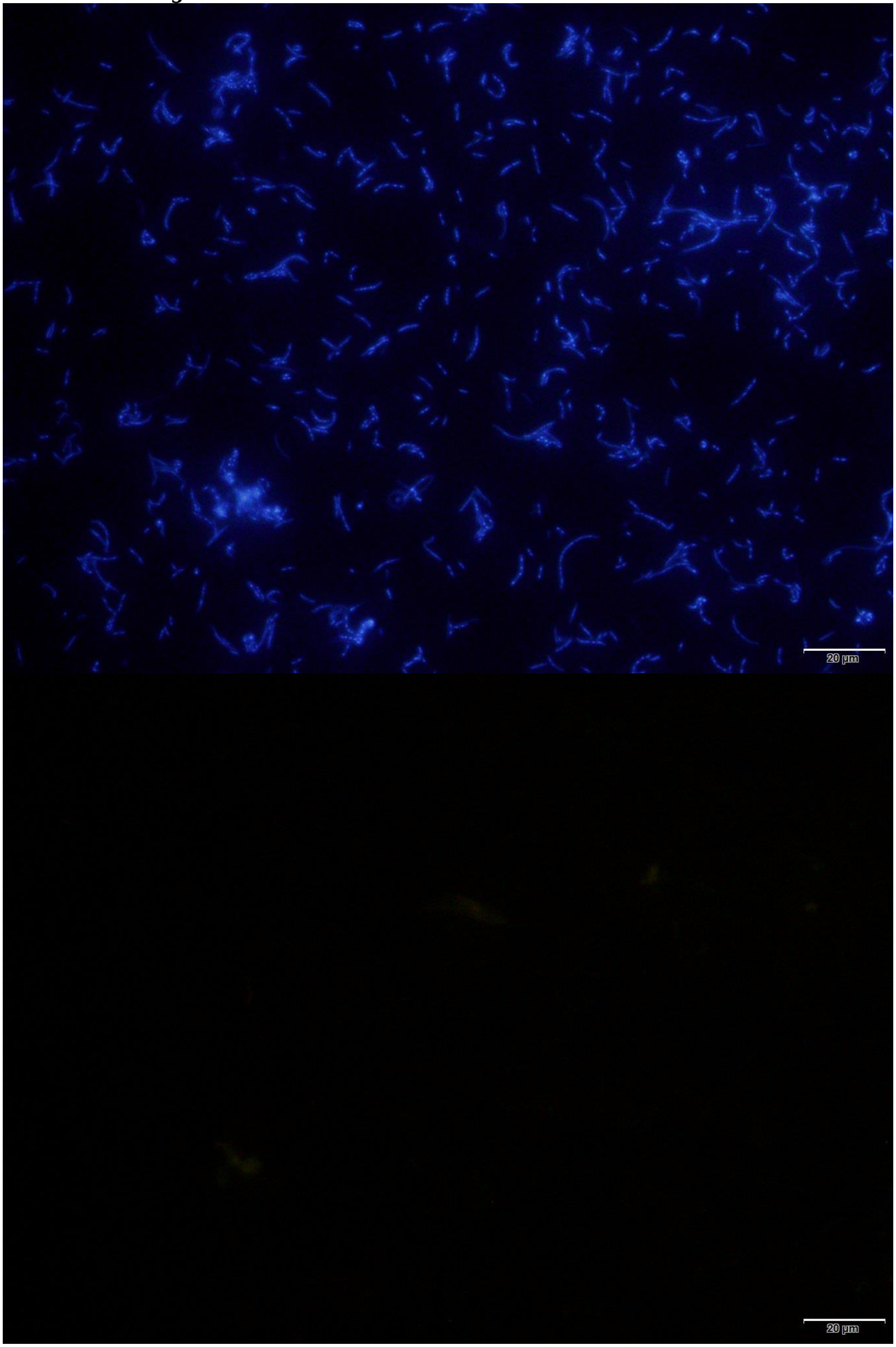

***Megasphaera micronuciformis* CCUG 45952T**

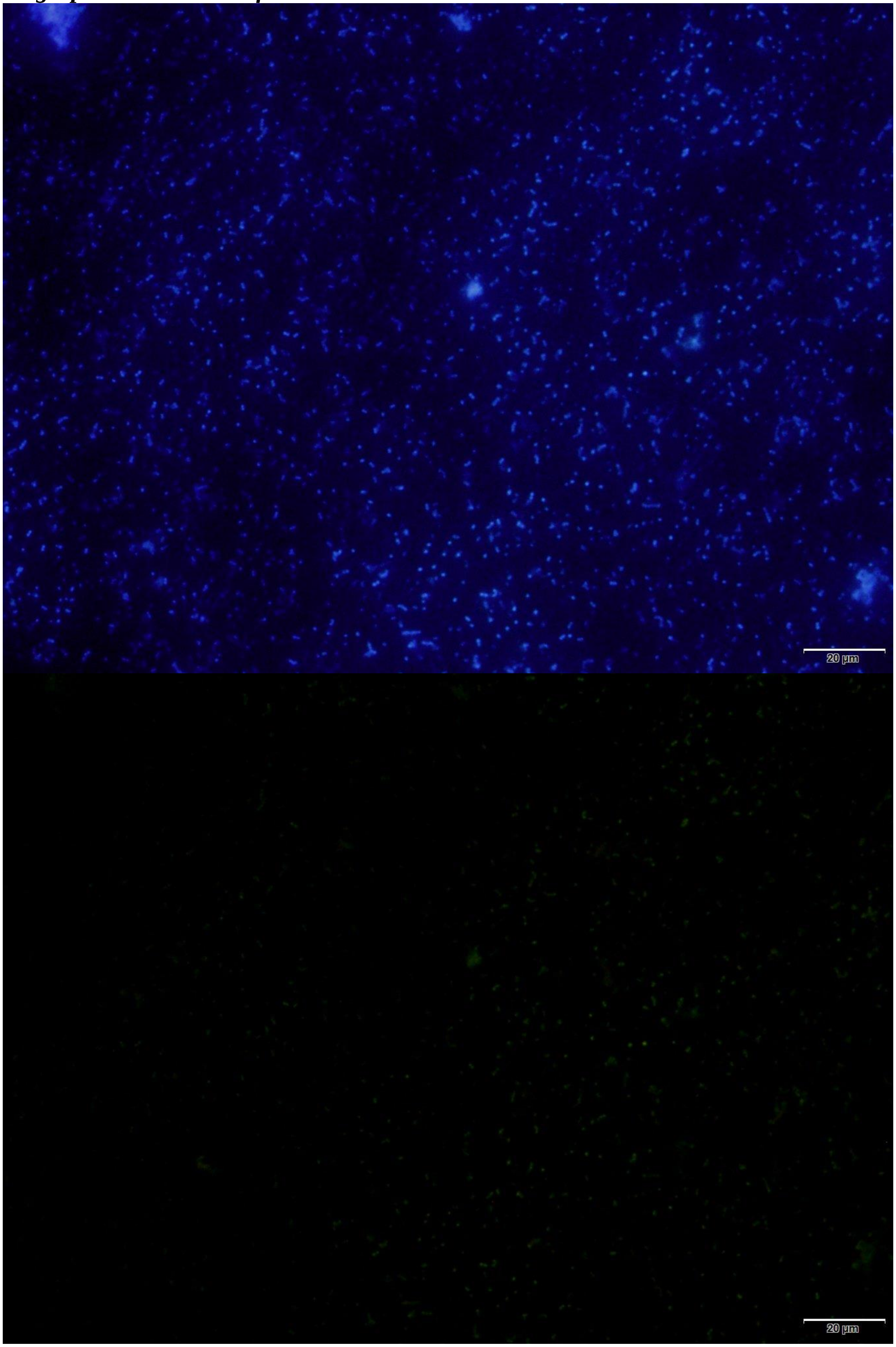

***Mobiluncus mulieris* ATCC 35239**

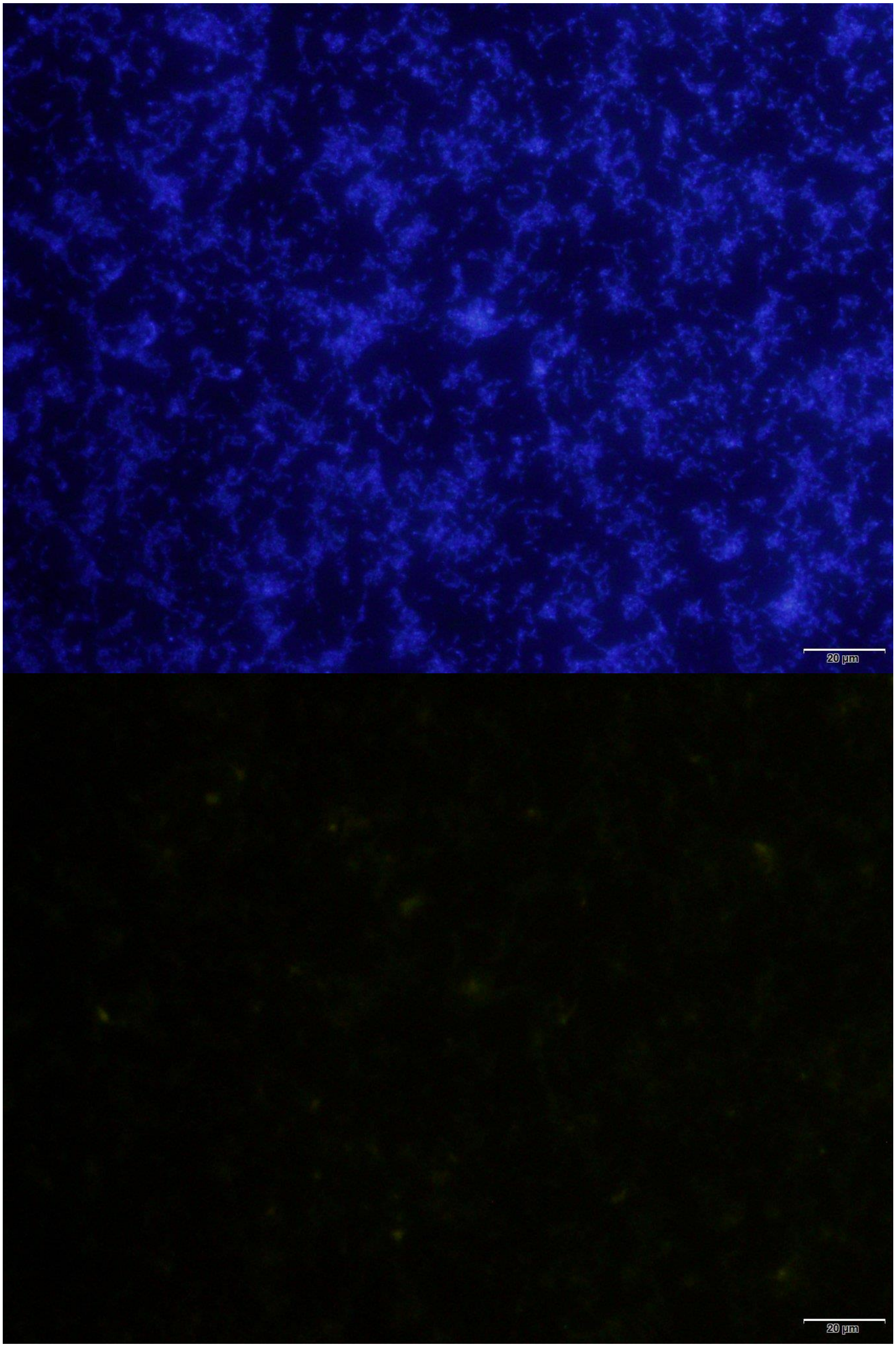

***Mycoplasma hominis* UM054**

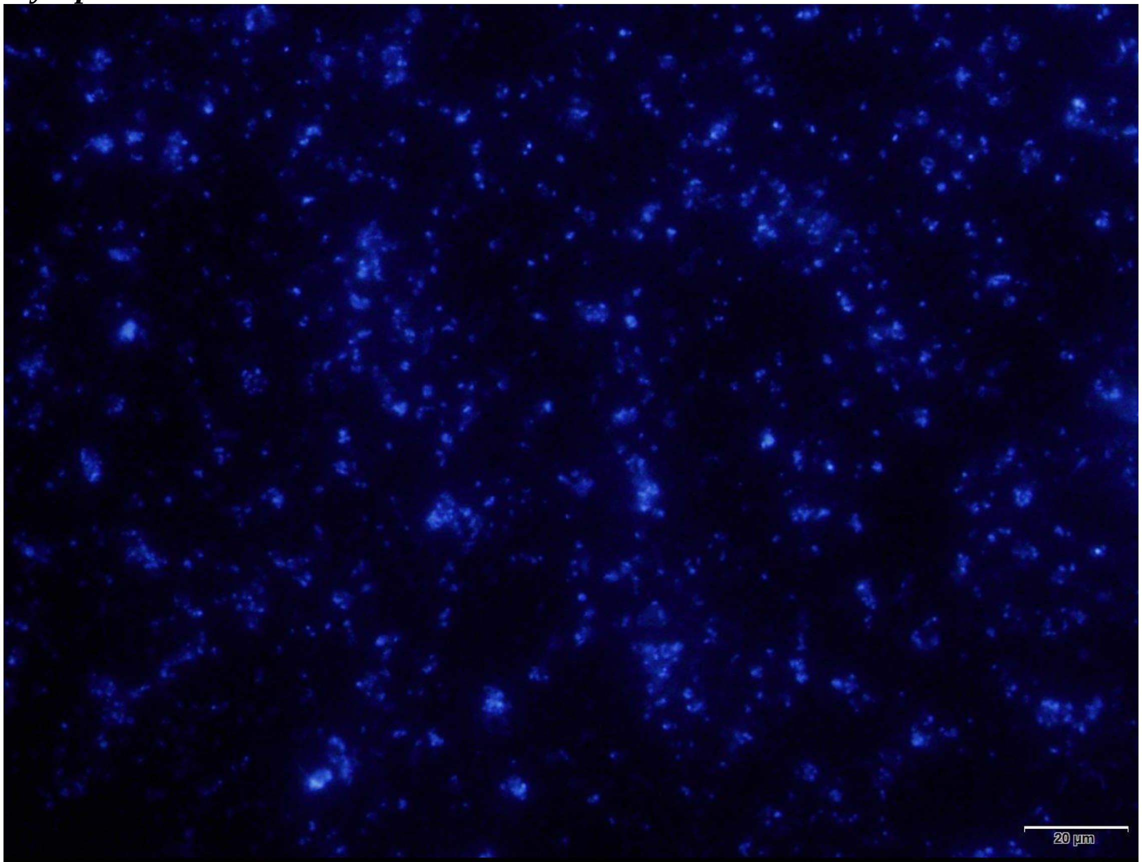

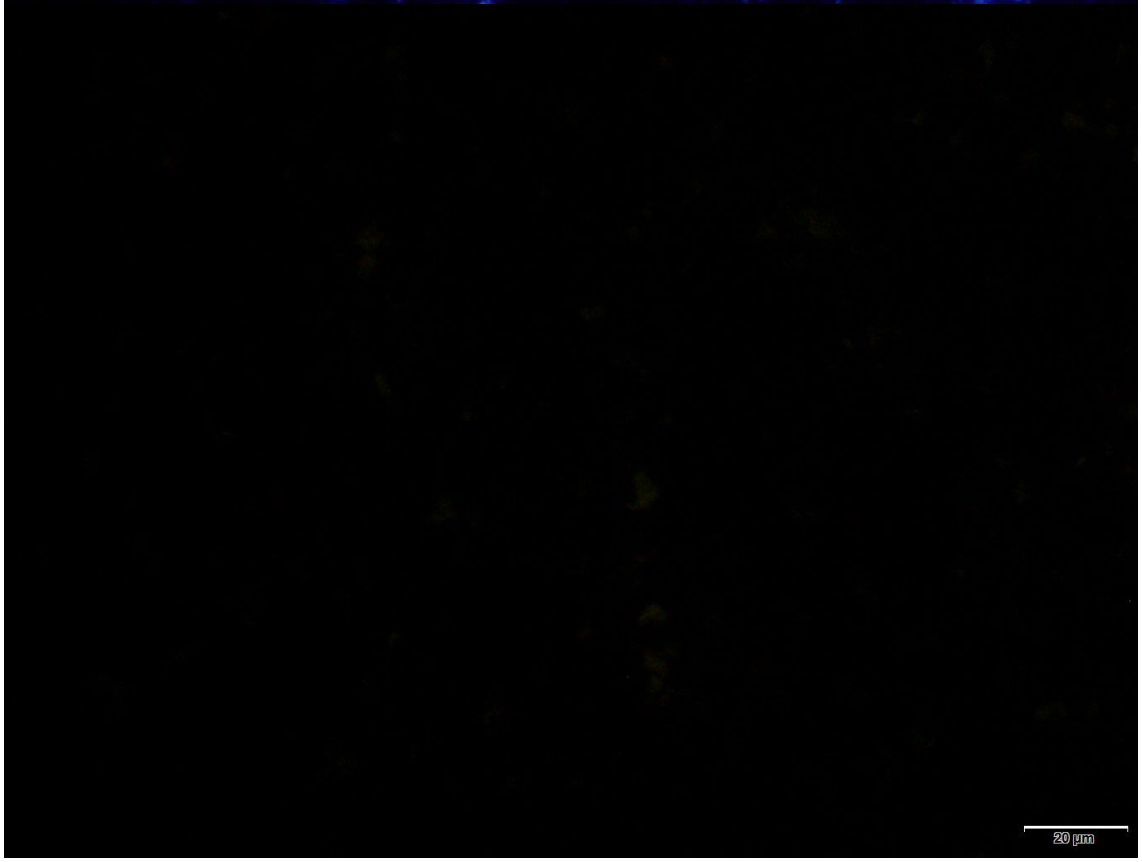

Neisseria gonorrhoeae **CCUG13281**

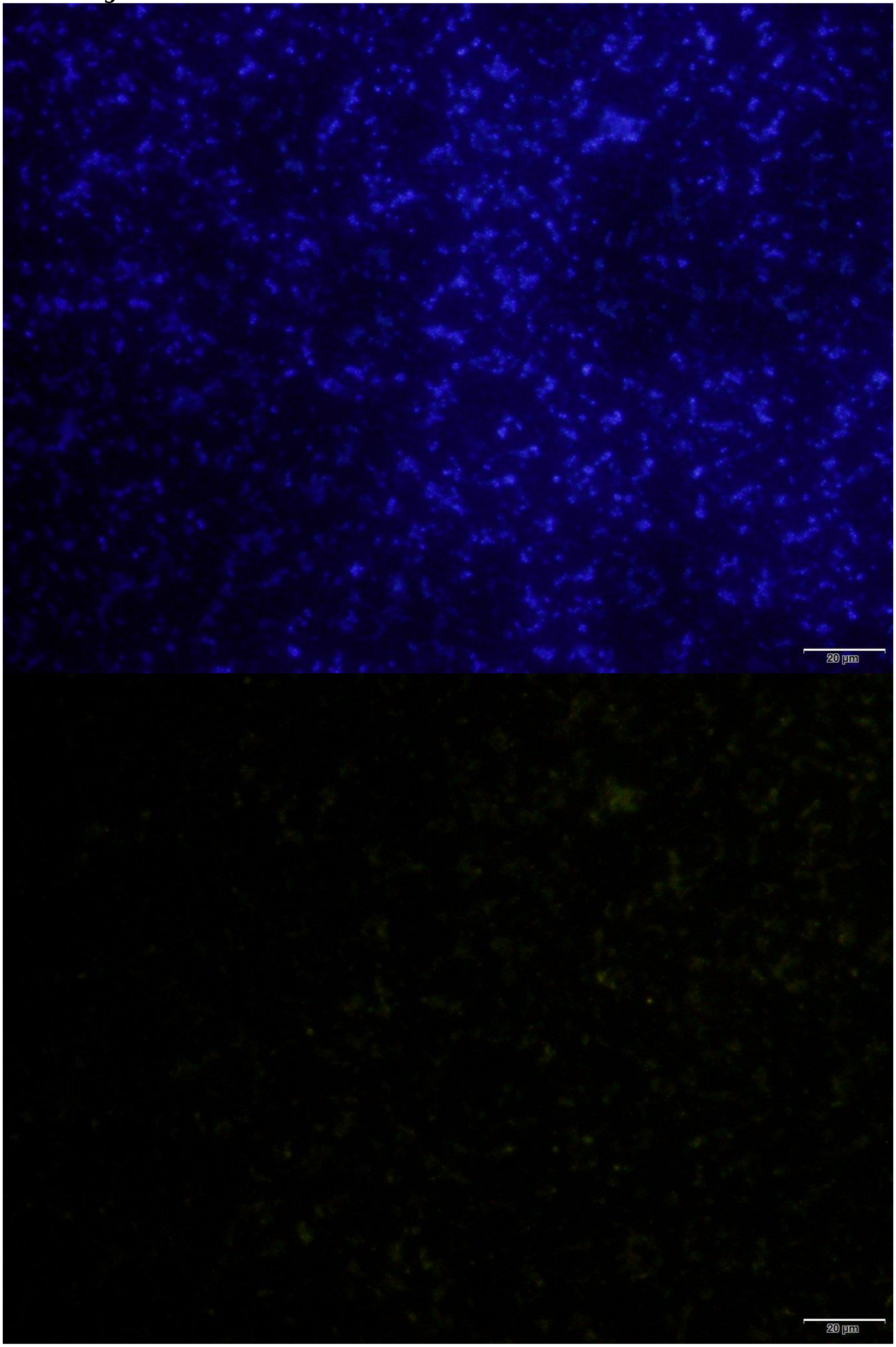

***Nosocomiicoccus ampullae* UM121**

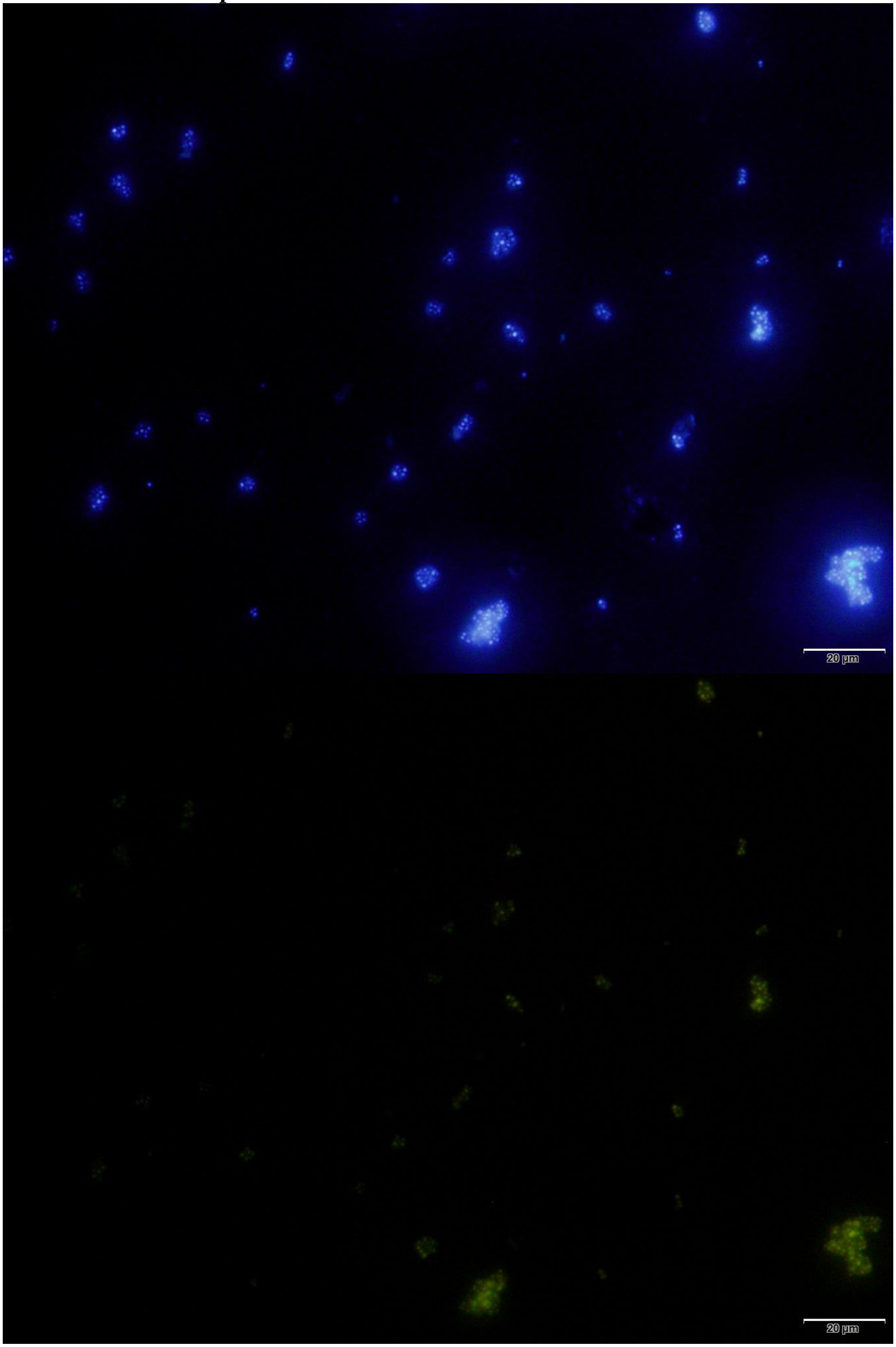

Porphyromonas asaccharolytica **CCUG7834**

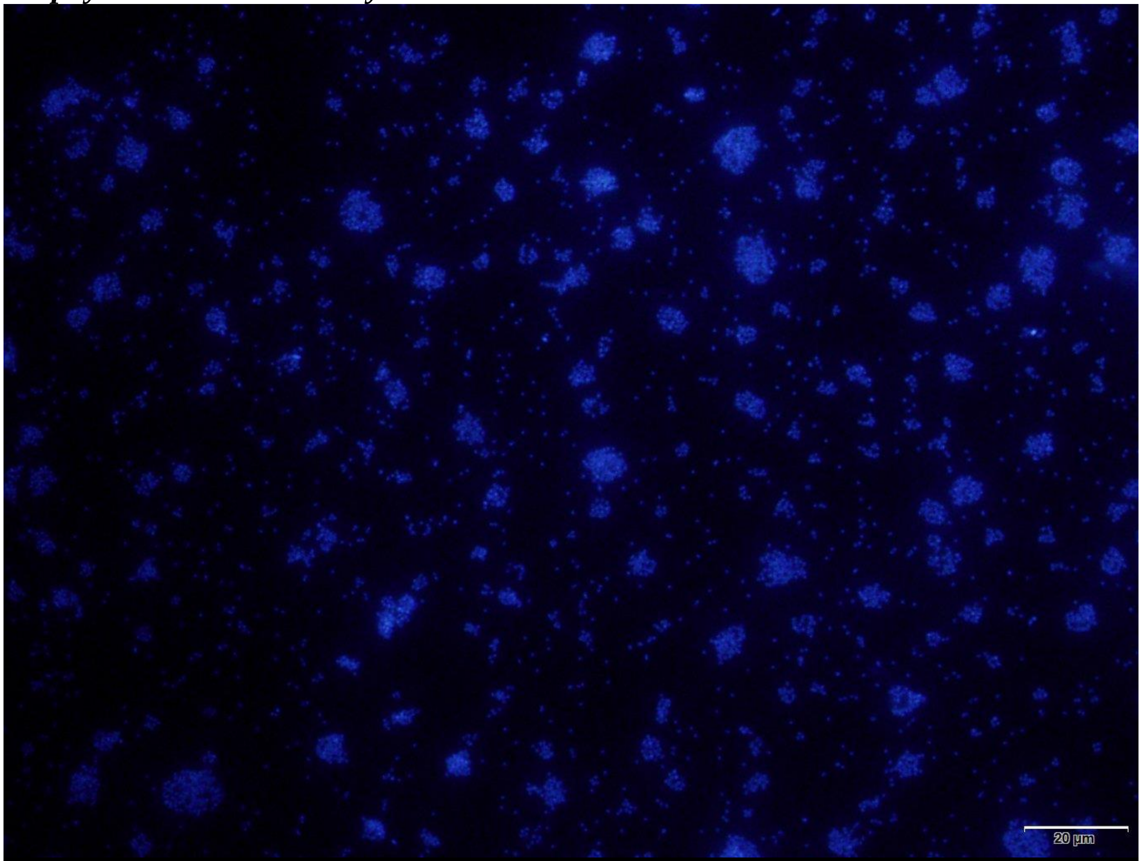

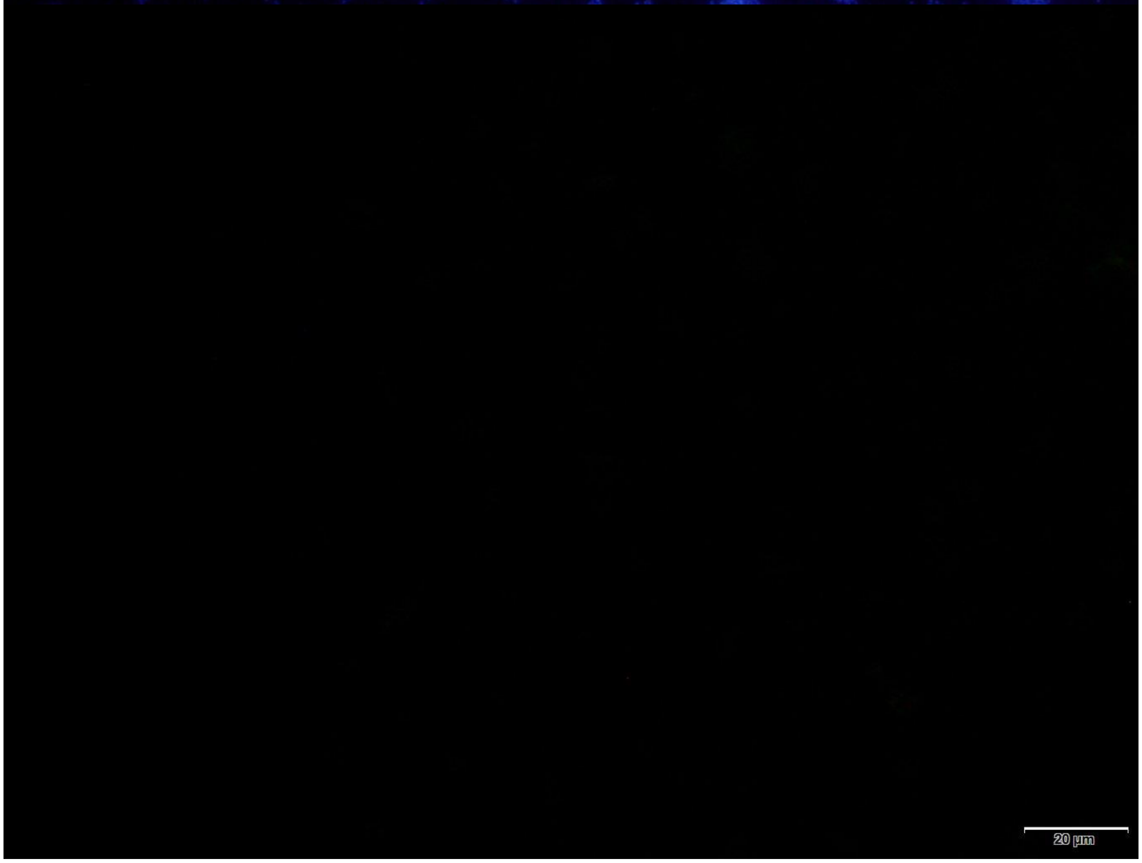

Propionibacterium acnes **UM034**

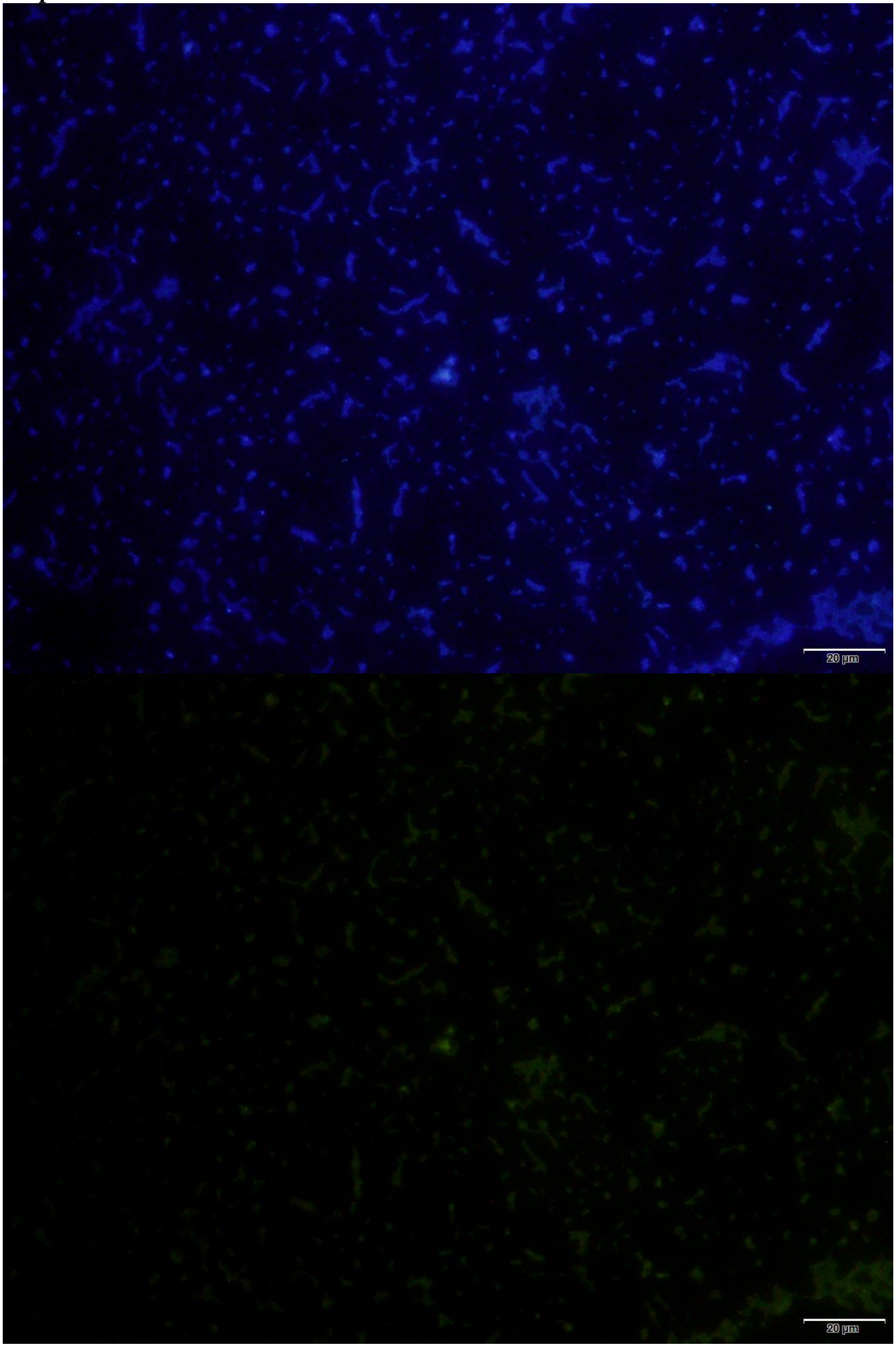

***Shigella* spp. UM137**

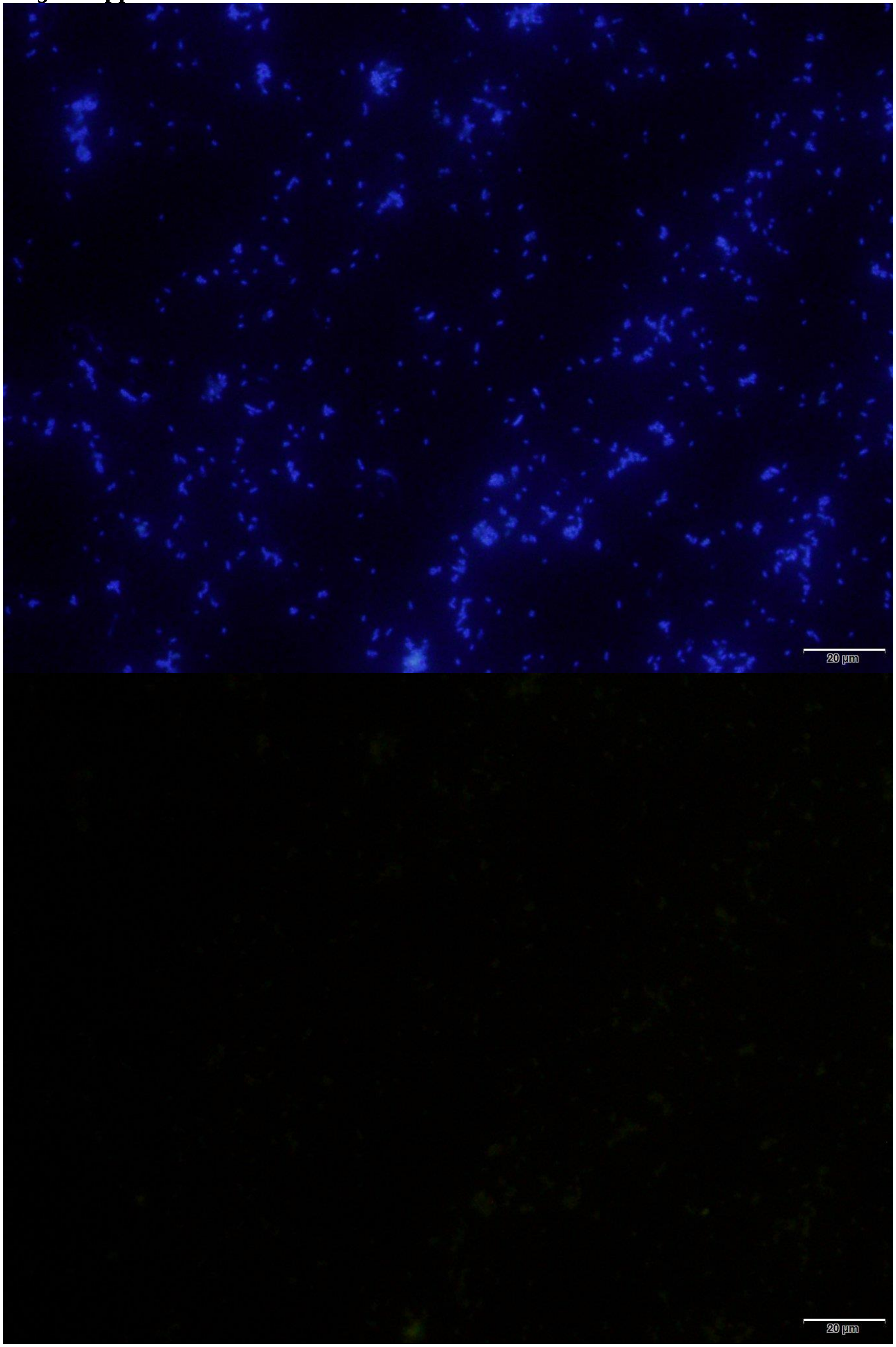

Sneathia sanguinegens **CCUG66076**

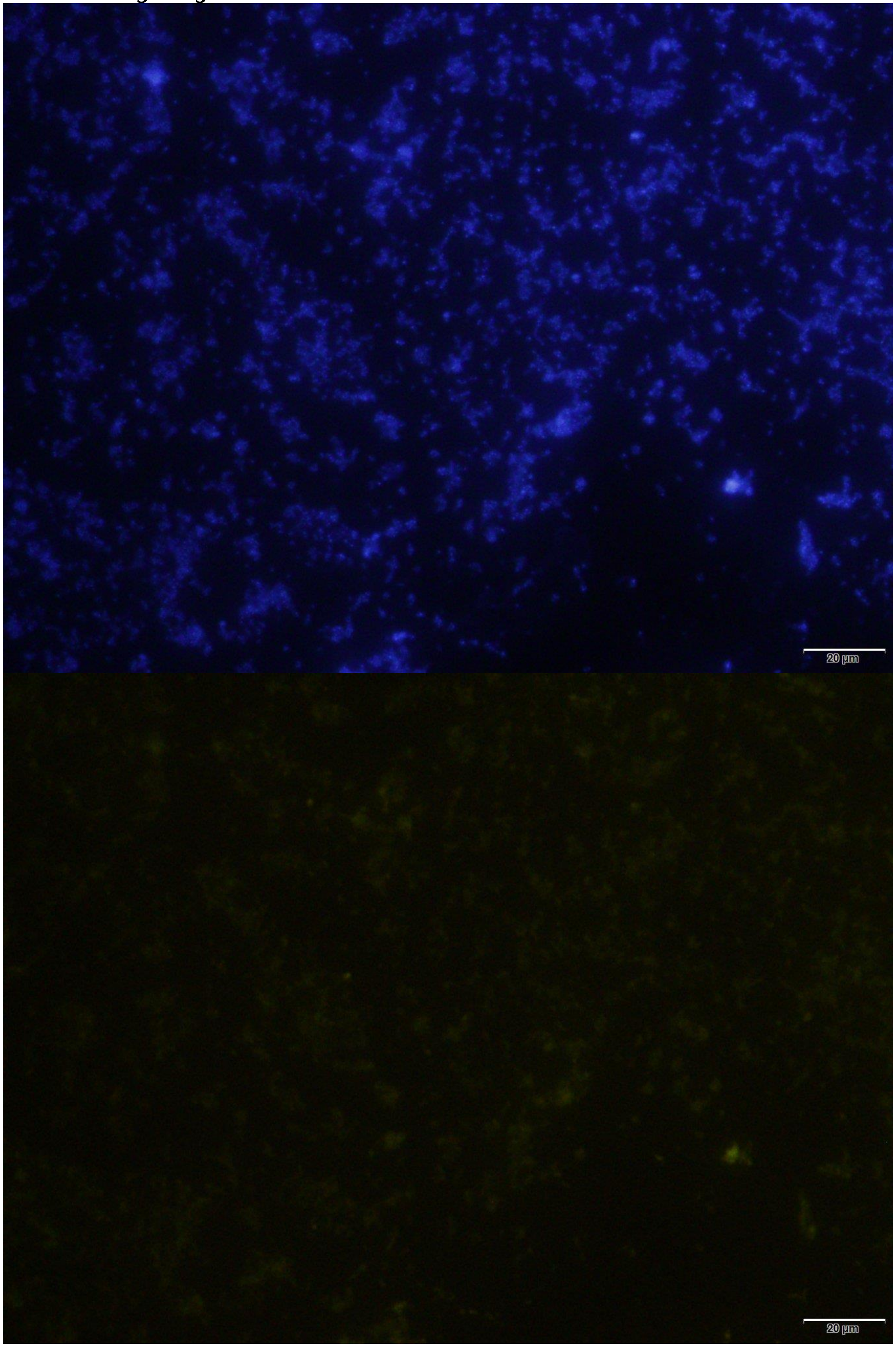

***Staphylococcus epidermidis* UM066**

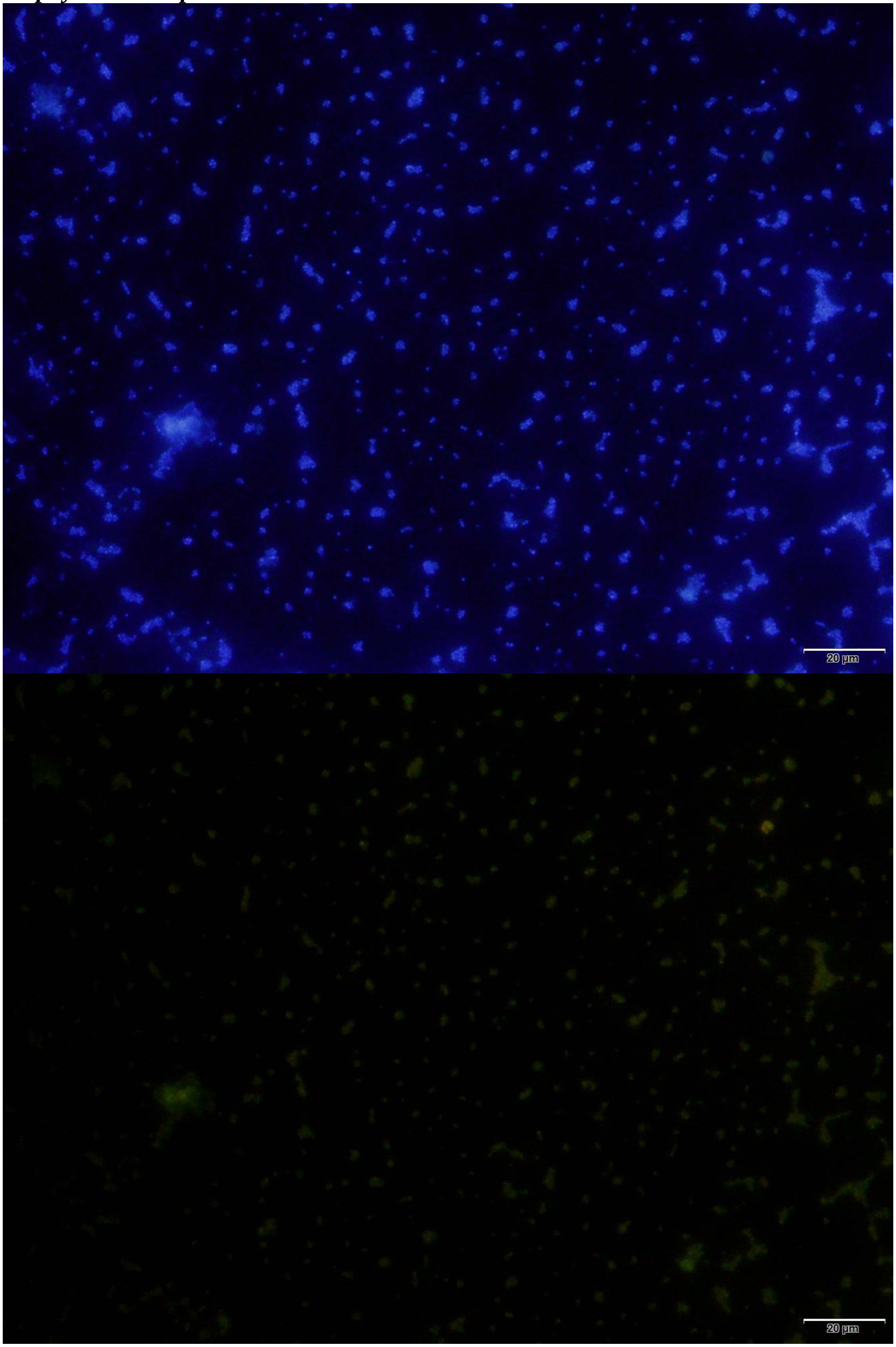

Staphylococcus haemolyticus **UM066**

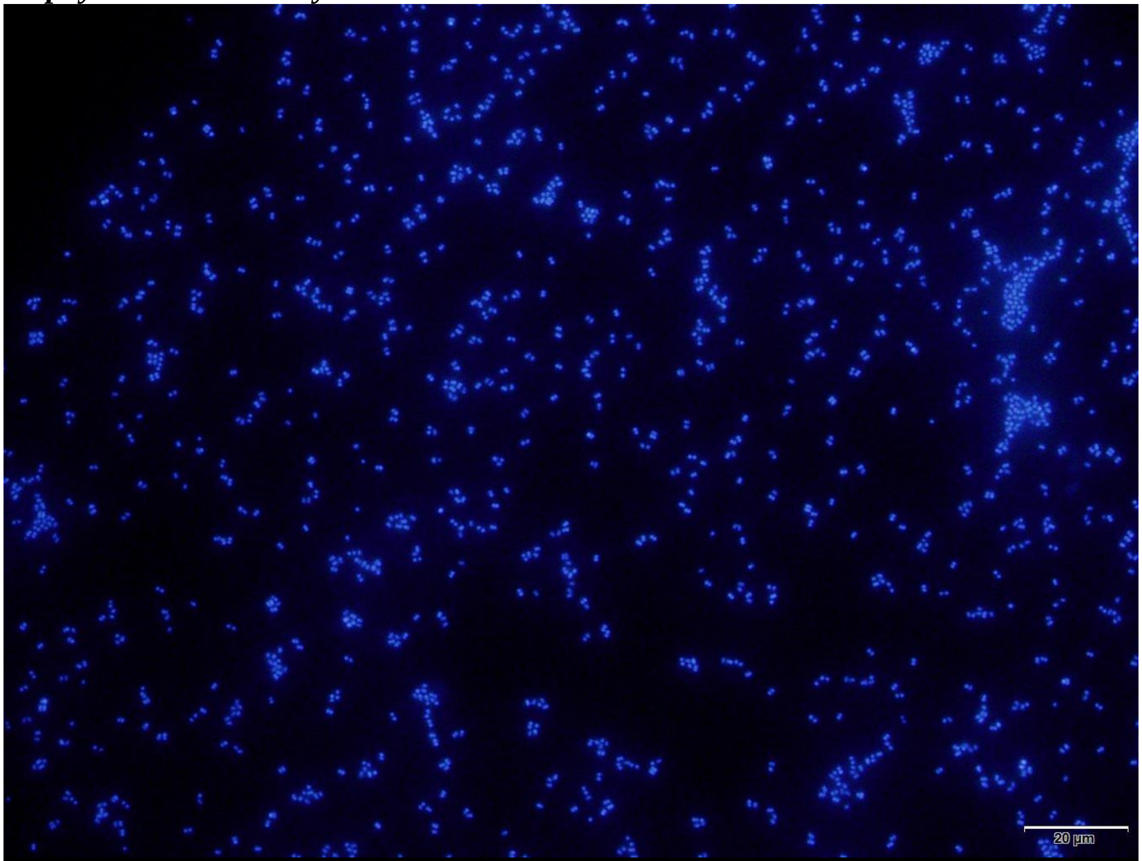

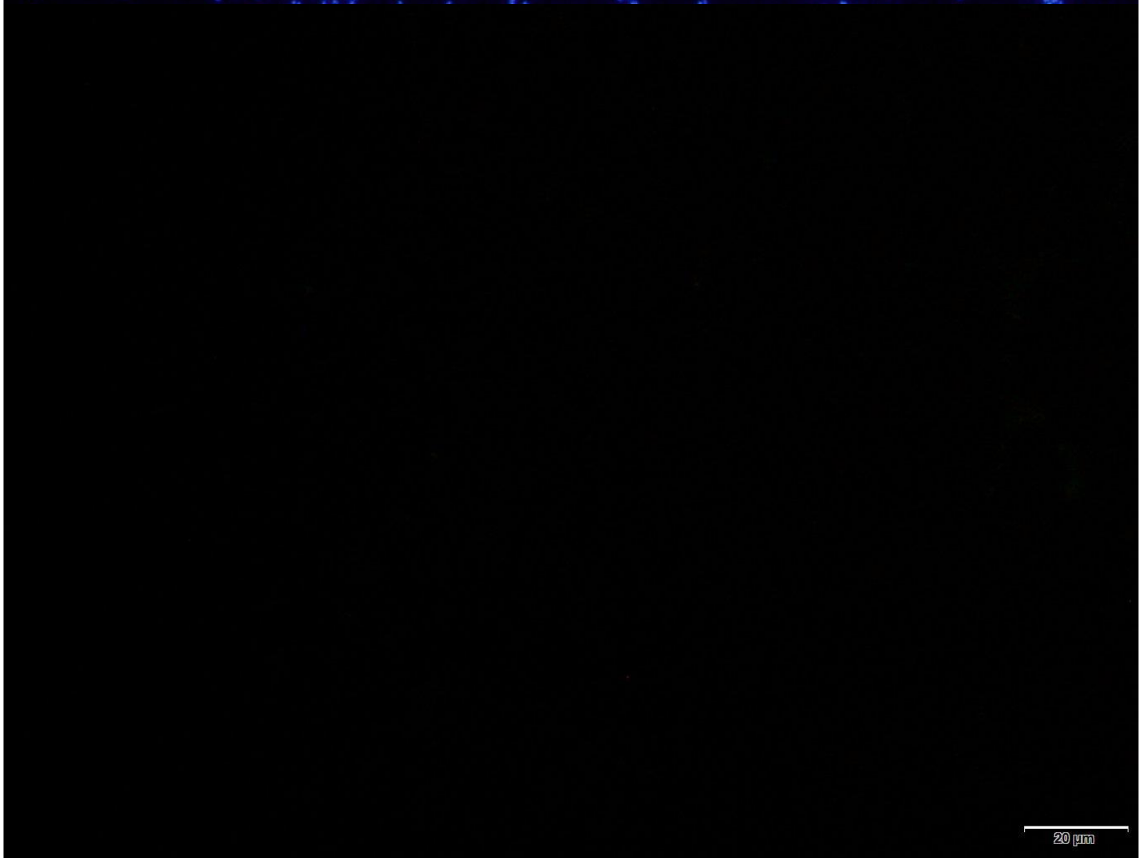

Staphylococcus hominis **UM224**

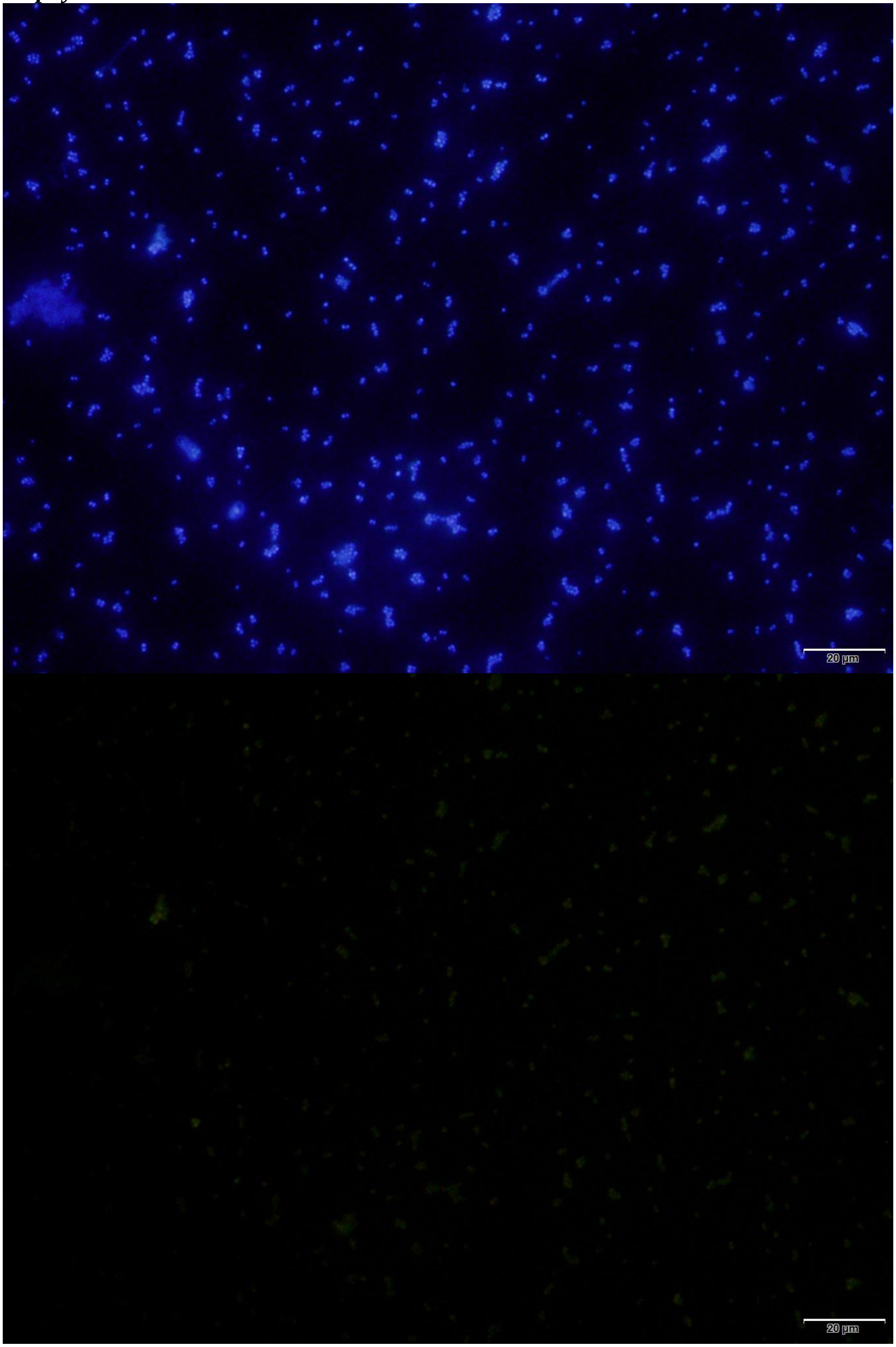

***Staphylococcus saprophyticus* UM121**

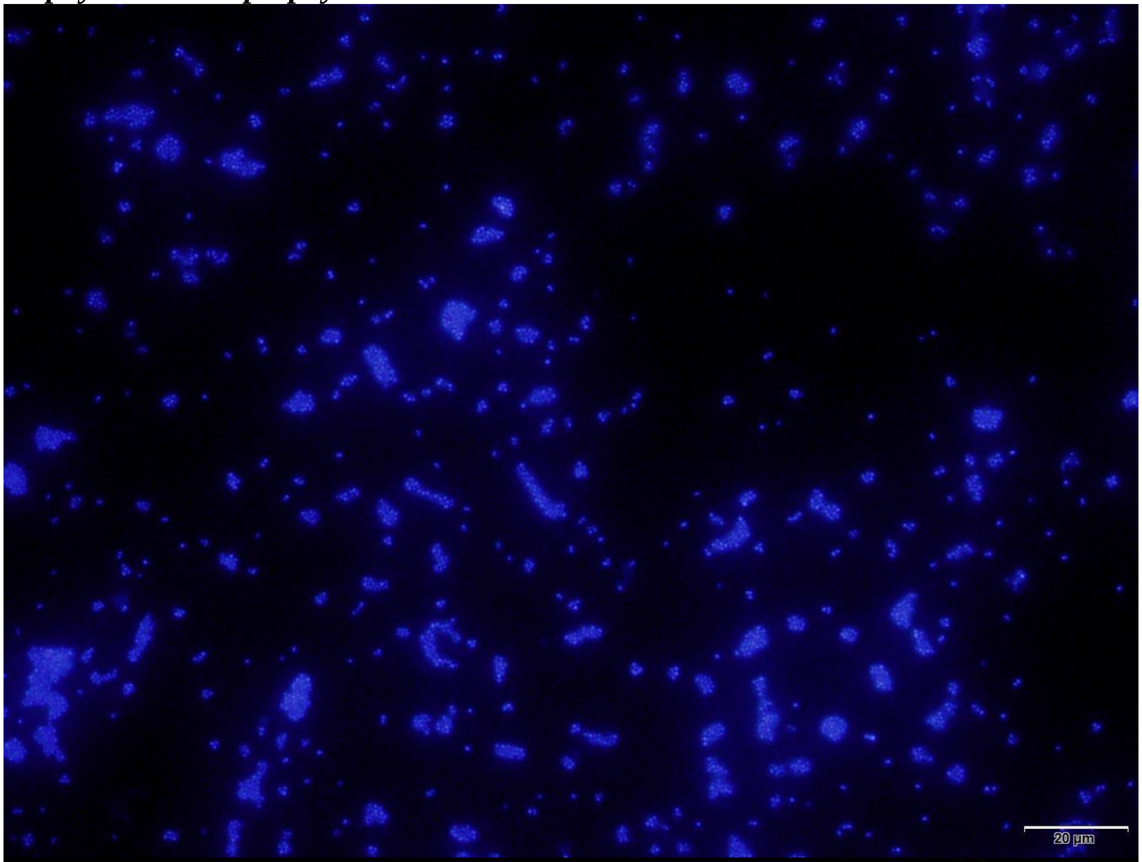

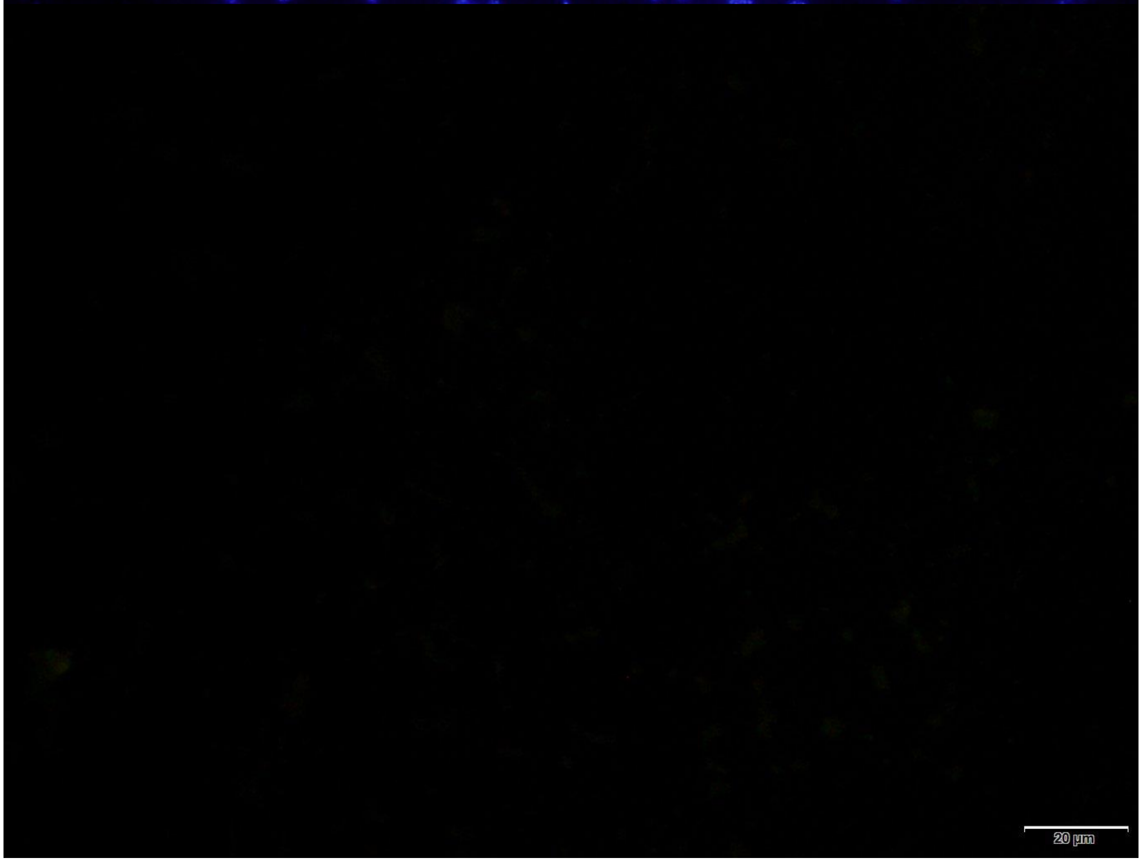

Staphylococcus simulans **UM059**

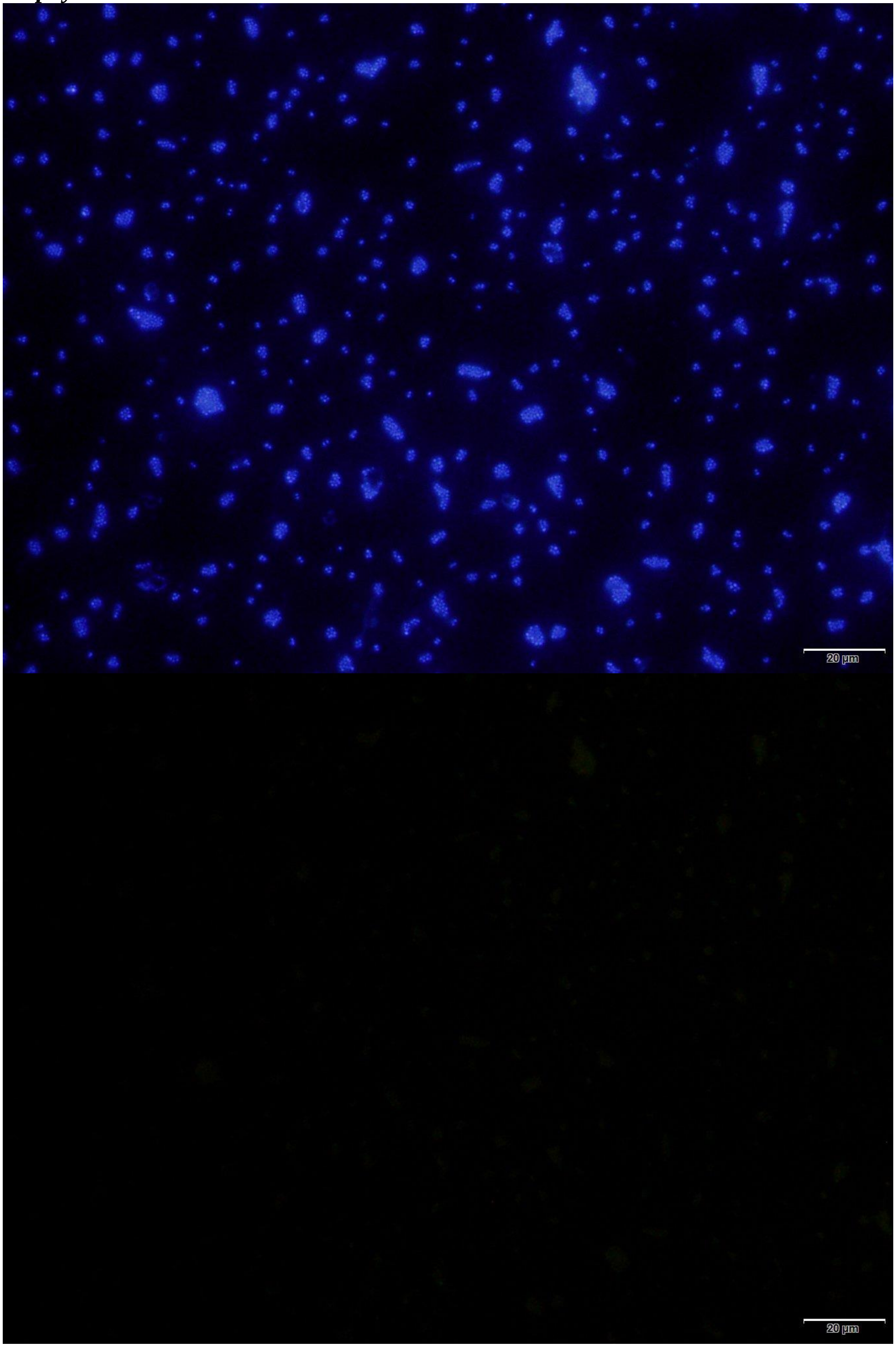

Streptococcus agalactiae **UM035**

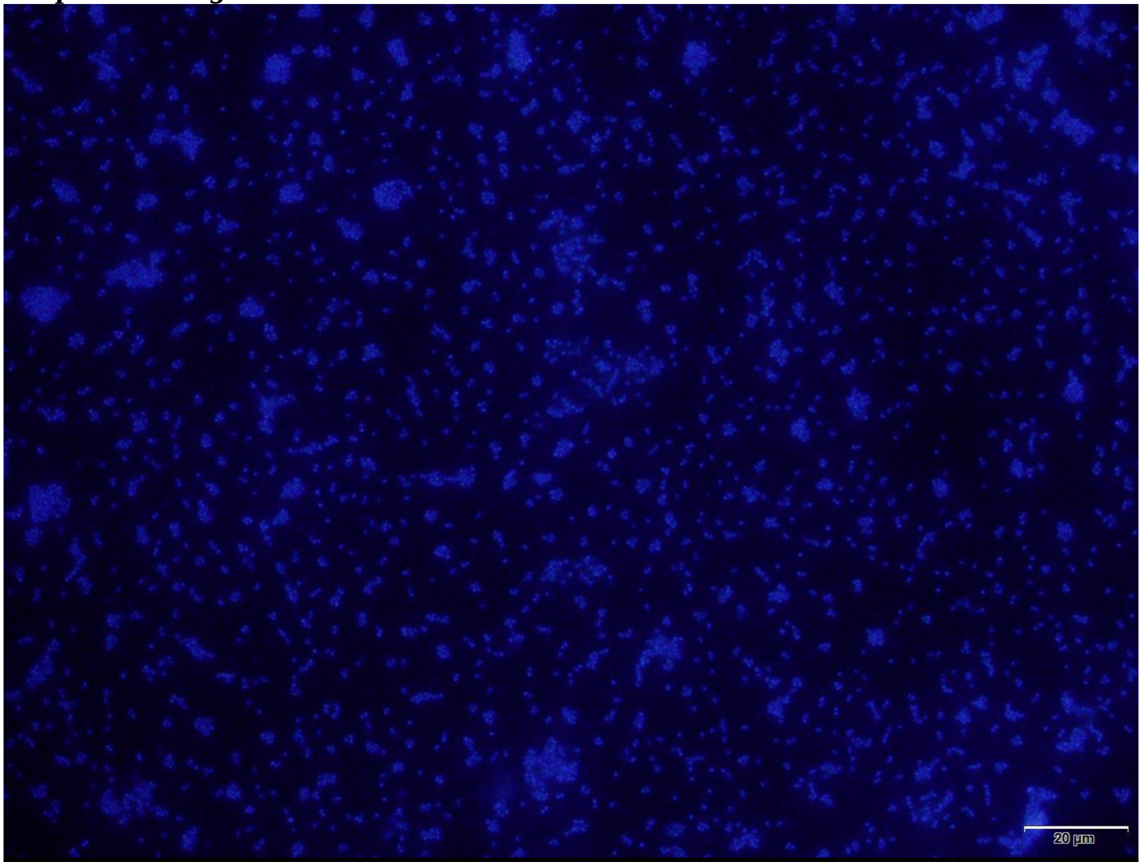

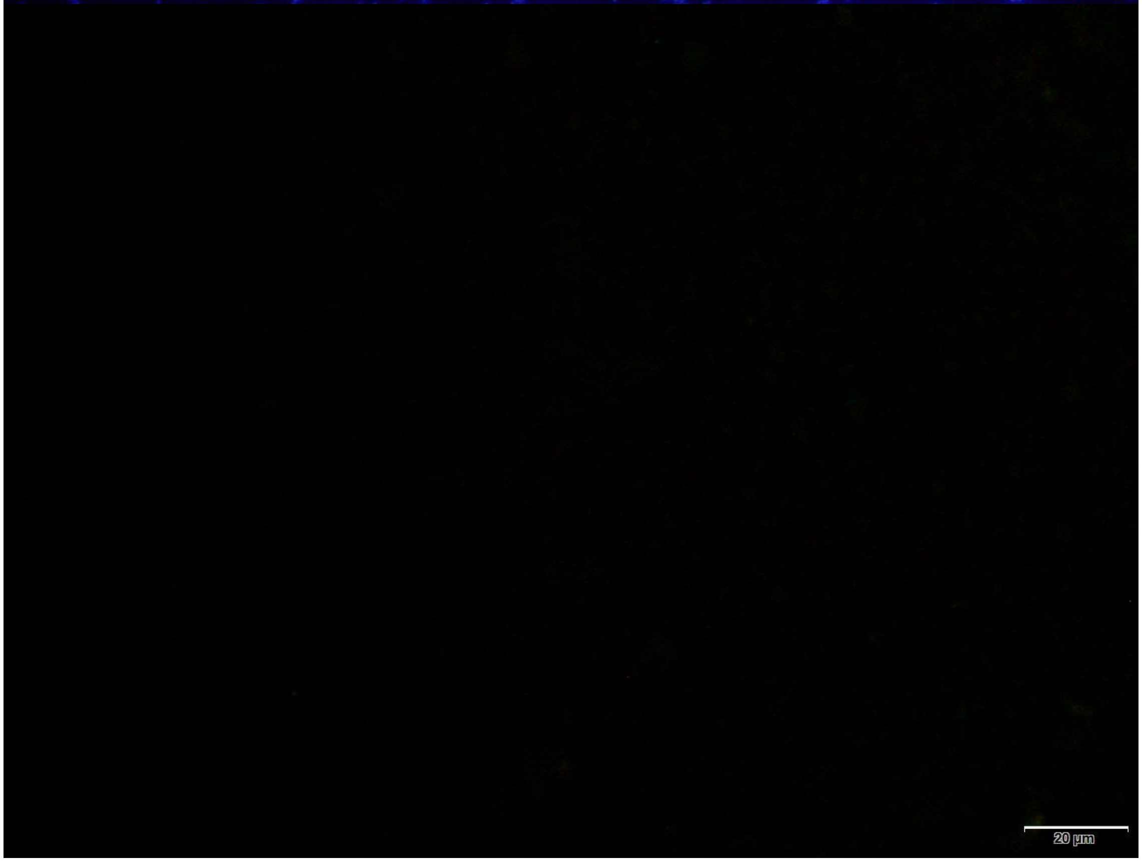

Veillonella parvula **CCUG 59474**

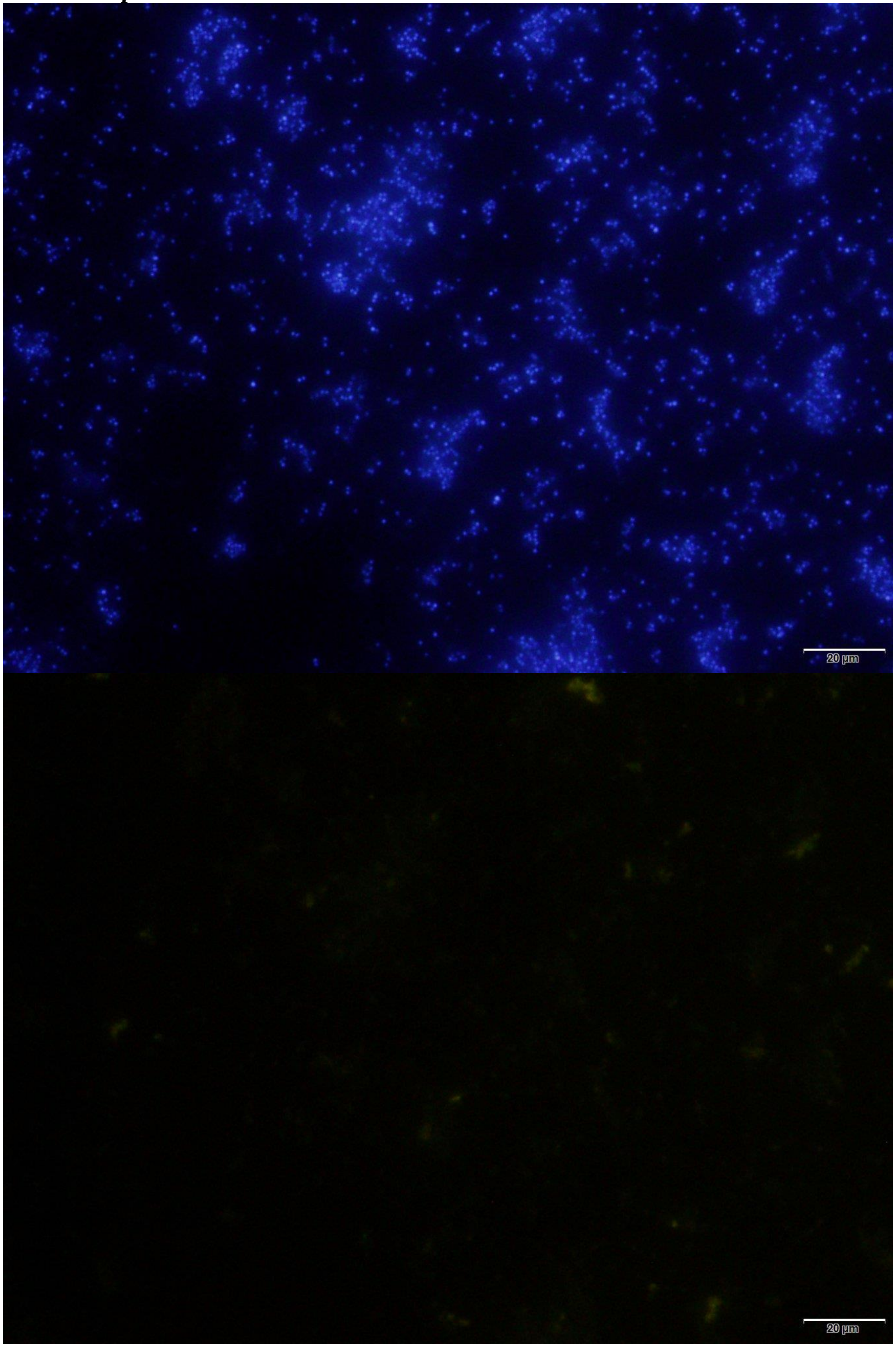

Supplement: Supplementary file 1 [file DataSheet_1.pdf]
